# The Diaryliodonium(III) Salts Reaction With Free-Radicals Enables One-Pot Double Arylation of Naphthols

**DOI:** 10.3389/fchem.2020.563470

**Published:** 2020-10-15

**Authors:** Yuvraj Satkar, Kazimierz Wrobel, Daniel E. Trujillo-González, Rafael Ortiz-Alvarado, J. Oscar C. Jiménez-Halla, César R. Solorio-Alvarado

**Affiliations:** ^1^División de Ciencias Naturales y Exactas, Departamento de Química, Universidad de Guanajuato, Guanajuato, Mexico; ^2^Facultad de Químico Farmacobiología, Universidad Michoacana de San Nicolás de Hidalgo, Morelia, Mexico

**Keywords:** diaryliodonium(III) salts, reaction with free-radicals, double arylation, electron-deficient hypervalent bond, one-pot double arylation

## Abstract

The chemoselective reaction of the *C*- followed by the *O-*centered naphthyl radicals with the more electron-deficient hypervalent bond of the diaryliodonium(III) salts is described. This discovered reactivity constitutes a new activation mode of the diaryliodonium(III) salts which enabled a one-pot doubly arylation of naphthols through the sequential ${\text{C}}_{sp}^{2}$-${\text{C}}_{sp}^{2}$/O-${\text{C}}_{sp}^{2}$ bond formation. The naphthyl radicals were generated in the reaction by the tetramethylpiperidinyl radical (TMP·) which resulted from the homolytic fragmentation of the precursor TMP_2_O. Experimental and DFT calculations provided a complete panorama of the reaction mechanism.

## Introduction

The chemistry of radicals is a powerful tool in organic synthesis allowing chemical transformations with high activation energy profiles via HAT (Capaldo and Ravelli, 2017), SET (Kita et al., 1994, 1996; Rosen and Percec, 2009) or SOMO (Beesson et al., 2007). Both *C*- and *O*-centered radical formation on the naphthol moiety are known processes carried out by metals such as Cu (Nakajima et al., 1999; Li et al., 2001), Ru (Irie et al., 2000), Fe (Egami and Katsuki, 2009; Narute et al., 2016), Cr (Nieves-Quinones et al., 2019), or V (Brodwel and Cheng, 1991; Hon et al., 2001; Lee et al., 2014; Kang et al., 2017). These radicals can also be generated electrochemically (Elsler et al., 2014) or through the radical anion sulfate (${\text{SO}}_{4}^{\cdot -}$) (More and Jeganmohan, 2015).

On the other hand, diaryliodonium(III) salts (Ar_2_IX) have emerged as an excellent source of aryl groups (Merrit and Olofsson, 2009) which successfully transfer one arene (Beringer and Mausner, 1958; Beringer and Chang, 1971; Wang et al., 2010; Malmgren et al., 2013; Stuart, 2017) with concomitant ArI release by reductive elimination at the iodine atom (III → I).

Both radical and Ar_2_IX strategies (Moteki et al., 2013; Wang and Studer, 2017; Ye et al., 2018) have been used in the preparation of aryl phenols which are important synthetic targets due to their relevance as biologically active molecules (Zofou et al., 2013; Ramadoss et al., 2016, 2018a,b, 2019; Gutierrez-Cano et al., 2017), reagents (Grzybowski et al., 2013), building blocks (Dreher et al., 2000), and organocatalyst scaffolds (Parmar et al., 2014).

In this regard, the seminal work of Barton using Bi(V) (Barton et al., 1981, 1982, 1987) is the first precedent describing the arylation of phenols. More recently, the ionic *O*-arylation of phenols using Ar_2_IOTf has been mainly documented by Olofsson (Jalalian et al., 2011a,b; Lindstedt et al., 2013, 2016; Merrit et al., 2018; Nahide and Solorio-Alvarado, 2017; Reitti et al., 2018), while the also ionic path for the *C*-arylation has been much less explored. In this case, only few examples are known. Quideau (Ozanne-Beaudenon and Quideau, 2005), described *C*- and *O*-arylation mixtures and Kalek (Ghosh et al., 2019) reported on the selective *C*-arylation of naphthols using fluorinated Ar_2_IOTf. In all these protocols, the stoichiometric use of a base is needed for the reaction giving rise to the monoarylation of naphthols by transferring one aryl group from Ar_2_IX via an ionic pathway (Oh et al., 1999).

**Scheme 1:**
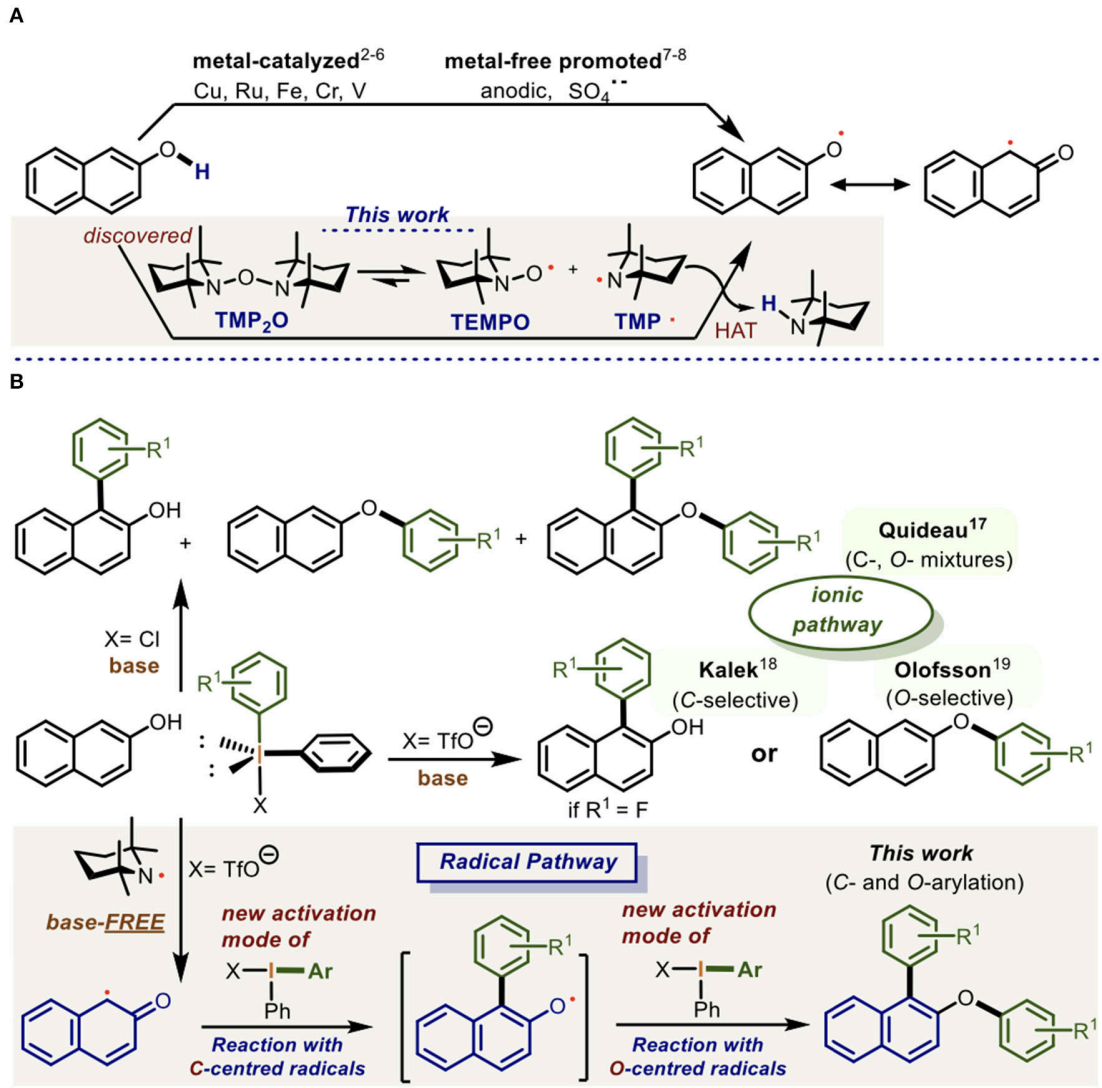
Procedures for the *O*-/*C*-centered radical formation in the 2-naphthol and its arylation using diaryliodonium salts. **(A)** Procedures fot the O-/C-centred radical formation at 2-napthol. **(B)** Procedures for arylation of naphthols mediated by diaryliodonium(III) salts.

In this context, we present for the first time, the direct reaction of Ar_2_IX at its more electron-deficient hypervalent bond (Lecroq et al., 2018) with naphthyl radicals (Np·) (Liu et al., 2012; Huang et al., 2014; Vaillant et al., 2015, 2016; Wang et al., 2015; Zhou et al., 2015) under base-free conditions. In this scenario, the Ar_2_IX behaves as a donor synthon of aryl radicals in the reaction with the *C*- and *O*-centered naphthyl radicals. This reactivity constitutes a new activation mode of the diaryliodonium(III) salts (Scheme 1).

Considering the synthetic importance of aryl phenols, we focused on this target as a part of our research in the development of new iodine(III)-based reactions (Nahide et al., 2018; Satkar et al., 2018, 2019; Juárez-Ornelas et al., 2019; Segura-Quezada et al., 2019). Herein, we report the recent advances of our approach using Ar_2_IX.

In the course of this work, we fortuitously discovered and later synthesized the new radical precursor TMP_2_O [1,1′-oxybis(2,2,6,6-tetramethylpiperidine)] which, according to our DFT calculations, spontaneously undergoes homolytic fragmentation leading to the formation of the TEMPO and tetramethylpiperidinyl (TMP^·^) radicals. In orthogonal fashion, the TMP^·^ radical reacts with 2-naphthol derivatives to produce an *O*-centered radical via HAT. This is in resonance with its *C*-centered radical (Scheme 1A) which, in a new activation mode, consecutively reacts with two equivalents of Ar_2_IX to generate a doubly arylated naphthol in a one-pot radical process (Scheme 1B).

## Materials and Methods

### General Information

All moisture- and oxygen-sensitive reactions were carried out in flame-dried round-bottom flasks under an inert atmosphere of nitrogen. Unless otherwise specified, all commercial materials were used as received without further purification. Anhydrous solvents were purchased from Sigma-Aldrich in Sure Seal bottles. Column chromatography was performed using silica gel of sizes 100–200 and 230–400 mesh (Sigma-Aldrich). Thin layer chromatography was performed with TLC silica gel 60 F256 plates, and visualization was done with short wavelength UV light (254 nm). Compounds were characterized using ^1^H and ^13^C NMR. (^1^H and ^13^C NMR spectra are provided for all the compounds in the SI.) Data of known compounds were compared with existing literature characterization data, and the references are given. ^1^H and ^13^C NMR spectra were recorded with 500 MHz and Bruker advance 400 MHz instruments using deuterated solvents purchased from Sigma-Aldrich like CDCl_3_. ^1^H spectra were referenced with tetramethyl silane (TMS, 0.0 ppm) or chloroform (CDCl_3_, 7.26 ppm) and are reported as follows: chemical shift, multiplicity (s = singlet, d = doublet, t = triplet, q = quartet, m = multiplet), coupling constant (Hz), and integration. Chemical shifts of the ^13^C NMR spectra were measured relative to CDCl_3_ (δ = 77.16 ppm). All the starting materials were synthesized according to reported procedures in the literature. High-resolution masses (HRMS) analyses were obtained under the following procedure: Samples were introduced by direct infusion at 3 μL min^−1^ to the electrospray ionization (ESI) source of a quadrupole time-of-flight mass spectrometer (Bruker Daltonics ESI-QTOF-MS maXis impact), equipped with Data Analysis 4.1. ESI was operated in positive mode with ion spray voltage 4 500 V, nitrogen dry gas 4 L min^−1^, drying temperature 180°C, and gas pressure 0.4 bar. Mass calibration was accomplished based on sodium formate clusters. Chemical nomenclature was generated using Chemdraw. Infrared (IR) spectra were recorded using PerkinElmer system 2000 FT-IR spectrometer. Melting points of solids were measured using a Fisher-Johns melting point apparatus.

The following boronic acids were purchased from Sigma Aldrich and used without additional purification: *p*-tolylboronic acid, phenylboronic acid, naphtylboronic acid, 4-fluorophenylboronic acid, 4-chlorophenylbronic acid, and (3-choloro-4-fluorophenyl) boronic acid.

### General Procedure for Suzuki-Miyaura Cross-Coupling

The starting materials of the examples **4, 6–10**, and **17–19** were synthesized by Suzuki-Miyaura cross-coupling according to the following procedure:

A 50 mL round bottom flask with a stir bar was fitted with a rubber septum and flame dried under high vacuum. The flask was purged with argon and charged with Pd(PPh_3_)_4_ (155.5 mg, 0.1 mmol), K_2_CO_3_ (580.46 mg, 4.2 mmol), 6-bromonaphthalen-2-ol (443.9 mg, 2.0 mmol), boronic acid (4.0 mmol), 10.0 mL 1,4-dioxane, and 2 mL of distilled water. The reaction mixture was then heated at 80°C for 8 h. After the reaction was cooled down to room temperature, the organic layer was separated, and the aqueous layer was extracted with ethyl acetate (3 × 10 mL), and the combined organic layer was dried over Na_2_SO_4_ and concentrated. The crude products were purified by flash chromatography on silica gel.

### General Procedure for the Double Arylation Using the System TMP_2_O/Ar_2_IX

A 25 mL round bottom flask with a stir bar was fitted with a rubber septum and flame dried under high vacuum. The flask was purged with argon and charged with the corresponding naphthols (0.25 mmol, 1 equiv), anhydrous diethyl ether and cyclohexane (1:1) (5 mL, 0.1 *M*) at 25°C. The corresponding amount of the solids mixture (1.575 g) containing TMP_2_O (126 mg, 0.425 mmol, 1.7 equiv) was added and stirred for 15 min obtaining a homogeneous mixture. Then, the diaryliodonium salt (0.625 mmol, 2.5 equiv) was added and stirred at 25°C until fully consumption of the starting material (usually 3 h). The reaction was quenched with a saturated solution of ammonium chloride. The organic layer was separated, and the aqueous layer was extracted with ethyl acetate (3 × 10 mL), the combined organic layers were dried over Na_2_SO_4_ and concentrated. The crude products were purified by flash chromatography on silica gel (10% EtOAc/Hexane) to afford the corresponding double arylated naphthol.

### Synthesis of the Radical Precursor TMP_2_O

A 250 mL round bottom flask equipped with a stir bar was fitted with a rubber septum and flame dried under high vacuum. The flask was purged with argon and charged with TMP-H (5.57 mL, 33.0 mmol, 1 equiv). Anhydrous *n*-hexane (66 mL, 0.5 *M*) was added, and mixture was cooled to −78°C (dry ice-acetone) for 5 min. Then *n*-BuLi (2.5 *M* in hexanes, 14.4 mL, 36 mmol, 1.1 equiv) was added dropwise and reaction mixture was stirred for 30 min at −78°C, then warmed up to room temperature and stirred overnight. The reaction mixture was directly evaporated without inert atmosphere keeping the water bath does not raise more than 20°C. Then, the concentrated reaction mixture was dried at high pressure to afford 2.2 g “*a brown, not pyrophoric and air stable solid*” product which corresponds to a 9:1 mixture of TMP-O-^*n*^Bu (1-butoxy-2,2,6,6-tetramethylpiperidine) (27.3%) and TMP_2_O [1,1′-oxybis(2,2,6,6-tetramethylpiperidine)] (3%) which was used without additional purification. m.p. = > 50°C dec.

IR (neat) ν/cm^−1^ = 3,137, 3,123, 3,068, 3,049, 1,330, 1,216, 1,198, 1,181, 1,127, 1,035.

### Diarylphenols of Scheme 2

#### 2-phenoxy-1-phenylnaphthalene (2a)

The following compound was obtained according to the general procedure by using 2-napthol and diphenyliodonium triflate as starting material. The crude material was purified by flash column chromatography over silica gel with the (2% EtOAc/Hexane) system to afford the product **2a** (72 mg, 74%) as a gel solid. R_*f*_ = 0.15 (4% EtOAc/Hexane). IR (neat) ν/cm^−1^ = 2,920, 1,585, 1,486, 1,232, 744. ^1^H NMR (500 MHz, CDCl_3_) δ 7.87 (dd, *J* = 11.8, 8.3 Hz, 2H), 7.63 (d, *J* = 8.4 Hz, 1H), 7.49–7.33 (m, 7H), 7.25–7.21 (m, 3H), 6.99 (t, *J* = 7.4 Hz, 1H), 6.87 (dd, *J* = 8.6, 0.9 Hz, 2H). ^13^C NMR (126 MHz, CDCl_3_) δ 158.6, 150.4, 135.5, 133.9, 131.1, 130.7, 130.3, 129.6, 129.4, 128.3, 128.4, 127.7, 126.6, 126.1, 125.5, 122.9, 120.7, 117.8. HRMS (EI): m/z calculated for C_22_H_17_O [M+H]^+^ = 297.1279, found 297.1299.

**Scheme 2:**
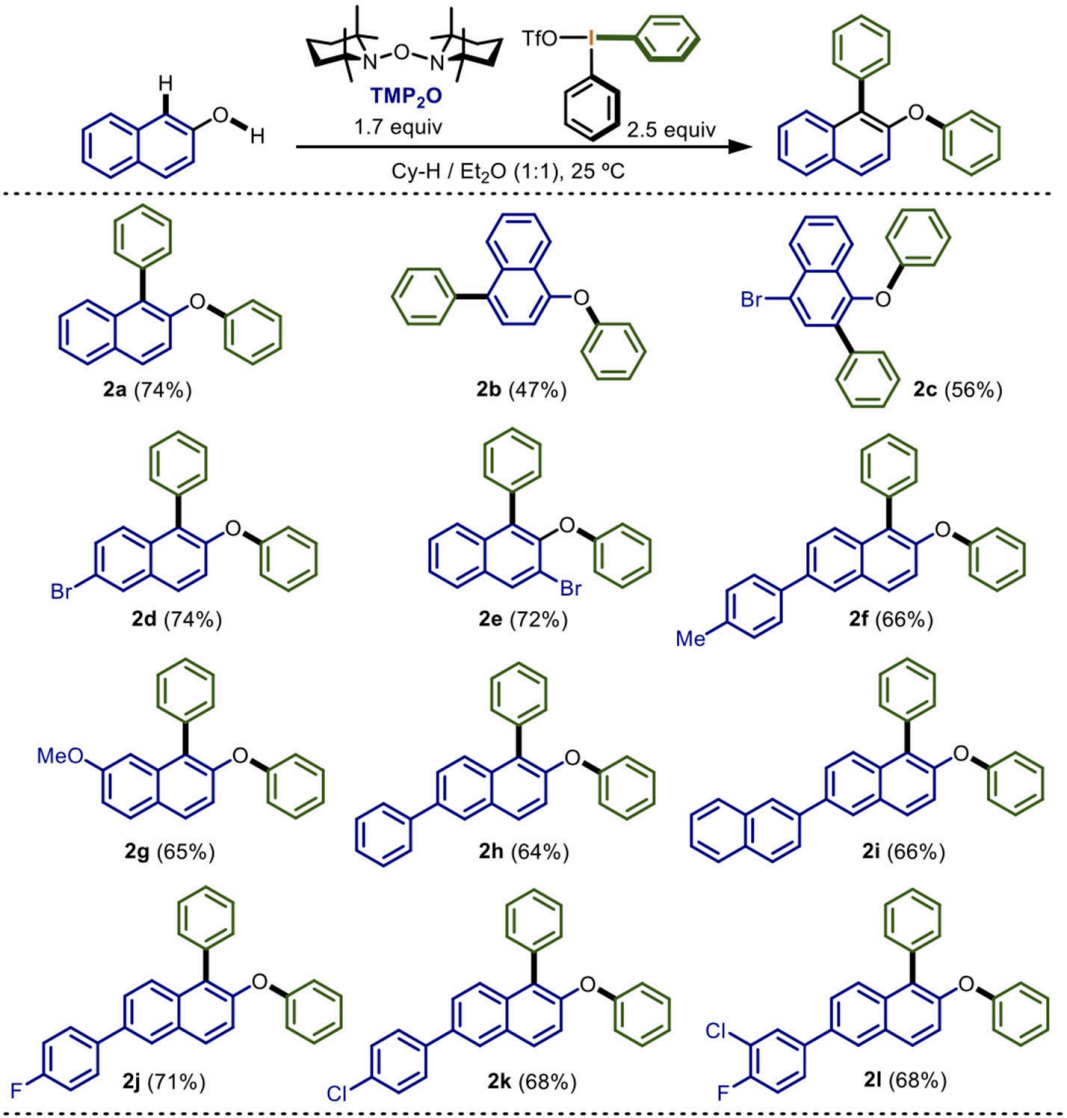
Scope of electronic nature of naphthols in the double arylation mediated by TMP_2_O/Ph_2_IOTf^*a,b*^.

#### 1-phenoxy-4-phenylnaphthalene (2b)

The following compound was obtained according to the general procedure A, by using naphthalen-1-ol and diphenyliodonium triflate as starting material. The crude material was purified by flash column chromatography over silica gel with the (2% EtOAc/Hexane) system to afford the product **2b** (48 mg, 47%) as a white solid. m.p. = 94–96°C. R_*f*_ = 0.15 (5% EtOAc/Hexane). IR (neat) ν/cm^−1^ = 2,910, 1,588, 1,486, 1,232, 748. ^1^H NMR (500 MHz, CDCl_3_) δ 7.90 (d, *J* = 8.4 Hz, 1H), 7.83 (d, *J* = 8.2 Hz, 1H), 7.75 (d, *J* = 8.5 Hz, 1H), 7.52 (d, *J* = 8.5 Hz, 1H), 7.50–7.47 (m, 2H), 7.44–7.40 (m, 1H), 7.36 (ddd, *J* = 8.1, 6.9, 1.2 Hz, 1H), 7.25 (dd, *J* = 10.4, 4.7 Hz, 2H), 7.19–7.15 (m, 1H), 7.06–7.01 (m, 2H), 6.63 (t, *J* = 8.2 Hz, 1H), 6.61(d, *J* = 8.5 Hz, 2H). ^13^C NMR (126 MHz, CDCl_3_) δ 159.6, 146.7, 137.8, 134.4, 131.0, 129.8, 129.3, 128.6, 128.3, 128.4, 127.8, 127.1, 126.7, 126.4, 125.9, 123.1, 121.3, 115.8. HRMS (EI): m/z calculated for C_22_H_17_O [M+H]^+^ = 297.1279, found 297.1312.

#### 4-bromo-1-phenoxy-2-phenylnaphthalene (2c)

The following compound was obtained according to the general procedure A, by using 4-bromonaphthalen-1-ol and diphenyliodonium triflate as starting material. The crude material was purified by flash column chromatography over silica gel with the (3% EtOAc/Hexane) system to afford the product **2c** (47 mg, 56%) as a white solid. m.p. = 102–104°C. R_*f*_ = 0.45 (5% EtOAc/Hexane). IR (neat) ν/cm^−1^ = 3,010, 1,688, 1,566, 1,242, 758. ^1^H NMR (500 MHz, CDCl_3_) δ 8.27 (d, *J* = 8.5 Hz, 1H), 8.00 (d, *J* = 8.4 Hz, 1H), 7.93 (s, 1H), 7.62 (ddd, *J* = 8.3, 6.9, 1.2 Hz, 1H), 7.56–7.48 (m, 3H), 7.35–7.30 (m, 2H), 7.27 (t, *J* = 1.3 Hz, 1H), 7.14–7.08 (m, 2H), 6.87 (dd, *J* = 10.6, 4.1 Hz, 1H), 6.71–6.66 (m, 2H). ^13^C NMR (126 MHz, CDCl_3_) δ 158.8, 146.7, 136.7, 132.7, 132.8, 132.2, 129.8, 129.6, 129.4, 128.4, 127.9, 127.8, 127.55, 127.52, 123.8, 121.7, 119.8, 115.9. HRMS (EI): m/z calculated for C_22_H_16_BrO [M+H]^+^ = 375.0385, found 375.0376.

#### 6-bromo-2-phenoxy-1-phenylnaphthalene (2d)

The following compound was obtained according to the general procedure A, by using 6-bromonaphthalen-2-ol and diphenyliodonium triflate as starting material. The crude material was purified by flash column chromatography over silica gel with the (3% EtOAc/Hexane) system to afford the product **2d** (62 mg, 74%) as a white solid. m.p. = 94–96°C. R_*f*_ = 0.55 (5% EtOAc/Hexane). IR (neat) ν/cm^−1^ = 3,034, 1,581, 1,484, 1,226, 825. ^1^H NMR (500 MHz, CDCl_3_) δ 8.02 (d, *J* = 1.9 Hz, 1 H), 7.75 (d, *J* = 8.9 Hz, 1H), 7.49 (d, *J* = 9.1 Hz, 1H), 7.46 (d, *J* = 2.0 Hz, 1H), 7.45–7.33 (m, 6H), 7.25–7.22 (m, 2H), 7.01 (t, *J* = 7.4 Hz, 1H), 6.86 (dd, *J* = 8.7, 0.9 Hz, 2H). ^13^C NMR (126 MHz, CDCl_3_) δ 158.3, 150.8, 134.9, 132.6, 132.3, 130.6, 130.9, 130.3, 129.8, 129.9, 128.4, 128.5, 127.9, 127.7, 122.9, 121.9, 119.7, 117.9. HRMS (EI): m/z calculated for C_22_H_16_BrO [M+H]^+^ = 375.0385, found 375.0377.

#### 3-bromo-2-phenoxy-1-phenylnaphthalene (2e)

The following compound was obtained according to the general procedure A, by using 3-bromonaphthalen-2-ol and diphenyliodonium triflate as starting material. The crude material was purified by flash column chromatography over silica gel with the (2% EtOAc/Hexane) system to afford the product **2e** (61 mg, 72%) as a white solid. m.p. = 92–94°C. R_*f*_ = 0.45 (5% EtOAc/Hexane). IR (neat) ν/cm^−1^ = 3,035, 2,918, 1,582, 1,456, 1,201, 868. ^1^H NMR (500 MHz, CDCl_3_) δ 8.23 (s, 1H), 7.84 (d, *J* = 8.2 Hz, 1H), 7.50 (ddd, *J* = 9.1, 8.1, 4.8 Hz, 2H), 7.41 (ddd, *J* = 8.3, 6.9, 1.2 Hz, 1H), 7.36–7.29 (m, 3H), 7.25–7.20 (m, 2H), 7.16–7.08 (m, 2H), 6.90 (t, *J* = 7.4 Hz, 1H), 6.63 (dd, *J* = 8.7, 0.9 Hz, 2H).

^13^C NMR (126 MHz, CDCl_3_) δ 158.7, 146.8, 134.8, 134.4, 133.7, 132.5, 132.7, 130.9, 129.3, 128.1, 127.8, 127.2, 126.8, 126.9, 126.4, 121.8, 117.3, 115.6. HRMS (EI): m/z calculated for C_22_H_16_BrO [M+H]^+^ = 375.0385, found 375.0378.

#### 2-phenoxy-1-phenyl-6-(p-tolyl)naphthalene (2f)

The following compound was obtained according to the general procedure A, by using 6-(*p*-tolyl)naphthalen-2-ol and diphenyliodonium triflate as starting material. The crude material was purified by flash column chromatography over silica gel with the (3% EtOAc/Hexane) system to afford the product **2f** (54 mg, 66%) as a white solid. m.p. = 122–124°C. R_*f*_ = 0.15 (5% EtOAc/Hexane). IR (neat) ν/cm^−1^ = 3,058, 1,510, 1,487, 1,230, 742. ^1^H NMR (500 MHz, CDCl_3_) δ 8.06 (d, *J* = 1.3 Hz, 1H), 7.90 (d, *J* = 8.9 Hz, 1H), 7.70–7.65 (m, 2H), 7.61 (d, *J* = 8.1 Hz, 2H), 7.45–7.36 (m, 5H), 7.32–7.27 (m, 3H), 7.26–7.21 (m, 2H), 7.00 (t, *J* = 7.4 Hz, 1H), 6.88 (d, *J* = 7.9 Hz, 2H), 2.42 (s, 3H). ^13^C NMR (126 MHz, CDCl_3_) δ 158.7, 150.4, 138.1, 137.8, 137.4, 135.5, 132.9, 131.3, 130.7, 130.0, 129.8, 129.64, 129.62, 128.3, 127.5, 127.3, 126.6, 126.3, 125.6, 122.4, 121.2, 117.8, 21.9. HRMS (EI): m/z calculated for C_29_H_23_O [M+H]^+^ = 387.1749, found 387.1760.

#### 7-methoxy-2-phenoxy-1-phenylnaphthalene (2g)

The following compound was obtained according to the general procedure A, by using 7-methoxynaphthalen-2-ol and diphenyliodonium triflate as starting material. The crude material was purified by flash column chromatography over silica gel with the (2% EtOAc/Hexane) system to afford the product **2g** (60 mg, 65%) as a white solid. m.p. = 122–124°C. R_*f*_ = 0.45 (4% EtOAc/Hexane). IR (neat) ν/cm^−1^ = 3,056, 1,934, 1,626, 1,459, 812. ^1^H NMR (500 MHz, CDCl_3_) δ 7.78 (d, *J* = 2.7 Hz, 1H), 7.76 (d, *J* = 2.6 Hz, 1H), 7.45–7.33 (m, 5H), 7.25–7.19 (m, 2H), 7.10 (ddd, *J* = 8.7, 8.3, 2.9 Hz, 2H), 7.01–6.96 (m, 1H), 6.92 (d, *J* = 2.5 Hz, 1H), 6.86 (dd, *J* = 8.6, 0.9 Hz, 2H), 3.71 (s, 3H). ^13^C NMR (126 MHz, CDCl_3_) δ 158.7, 158.8, 151.3, 135.6, 135.2, 130.7, 129.5, 129.4, 128.9, 128.8, 128.7, 127.9, 126.9, 122.2, 118.6, 117.7, 117.2, 104.7, 55.5. HRMS (EI): m/z calculated for C_23_H_19_O_2_ [M+H]^+^ = 327.1385, found 327.1391.

#### 2-phenoxy-1,6-diphenylnaphthalene (2h)

The following compound was obtained according to the general procedure A, by using 6-phenylnaphthalen-2-ol and diphenyliodonium triflate as starting material. The crude material was purified by flash column chromatography over silica gel with the (6% EtOAc/Hexane) system to afford the product **2 h** (54 mg, 64%) as a white solid. m.p. = 132–134°C. R_*f*_ = 0.55 (8% EtOAc/Hexane). IR (neat) ν/cm^−1^ = 3,051, 1,621, 1,588, 1,204, 757. ^1^H NMR (500 MHz, CDCl_3_) δ 8.08 (d, *J* = 1.4 Hz, 1H), 7.91 (d, *J* = 8.9 Hz, 1H), 7.74–7.66 (m, 4H), 7.49 (t, *J* = 7.7 Hz, 2H), 7.46–7.36 (m, 6H), 7.28 (d, *J* = 8.9 Hz, 1H), 7.25–7.22 (m, 2H), 7.00 (t, *J* = 7.4 Hz, 1H), 6.88 (d, *J* = 7.8 Hz, 2H). ^13^C NMR (126 MHz, CDCl_3_) δ 158.6, 150.6, 140.9, 137.8, 135.7, 133.5, 131.7, 130.7, 129.9, 129.7, 129.6, 129.5, 128.9, 128.3, 127.5, 127.8, 126.6, 126.8, 126.1, 122.6, 121.2, 117.8. HRMS (EI): m/z calculated for C_28_H_21_O [M+H]^+^ = 373.1592, found 373.1595.

#### 6-phenoxy-5-phenyl-2,2'-binaphthalene (2i)

The following compound was obtained according to the general procedure A, by using [2,2′-binaphthalen]-6-ol and diphenyliodonium triflate as starting material. The crude material was purified by flash column chromatography over silica gel with the (4% EtOAc/Hexane) system to afford the product **2i** (55 mg, 66%) as a white solid. m.p. = 126–128°C. R_*f*_ = 0.55 (6% EtOAc/Hexane). IR (neat) ν/cm^−1^ = 3,051, 1,621, 1,588, 1,204, 757. ^1^H NMR (500 MHz, CDCl_3_) δ 8.21 (d, *J* = 1.7 Hz, 1H), 8.16 (d, *J* = 0.7 Hz, 1H), 7.95 (dd, *J* = 15.2, 7.1 Hz, 3H), 7.90–7.86 (m, 2H), 7.81 (dd, *J* = 8.8, 1.8 Hz, 1H), 7.75 (d, *J* = 8.8 Hz, 1H), 7.55–7.37 (m, 7H), 7.29 (d, *J* = 8.9 Hz, 2H), 7.23 (t, *J* = 2.0 Hz, 1H), 7.01 (t, *J* = 7.4 Hz, 1H), 6.90 (d, *J* = 7.8 Hz, 2H). ^13^C NMR (126 MHz, CDCl_3_) δ 158.6, 150.8, 138.7, 137.9, 135.9, 133.9, 133.3, 132.8, 131.6, 130.7, 129.9, 129.7, 129.6, 128.7, 128.4, 128.3, 127.8, 127.7, 126.8, 126.6, 126.4, 126.3, 126.2, 126.1, 125.7, 122.5, 121.6, 117.8. HRMS (EI): m/z calculated for C_32_H_23_O [M+H]^+^ = 423.1749, found 423.1748.

#### 6-(4-fluorophenyl)-2-phenoxy-1-phenylnaphthalene (2j)

The following compound was obtained according to the general procedure A, by using 6-(4-fluorophenyl)naphthalen-2-ol and diphenyliodonium triflate as starting material. The crude material was purified by flash column chromatography over silica gel with the (4% EtOAc/Hexane) system to afford the product **2j** (58 mg, 71%) as a white solid. m.p. = 134–136°C. R_*f*_ = 0.15 (8% EtOAc/Hexane). IR (neat) ν/cm^−1^ = 3,060, 1,734, 1,588, 1,487, 826. ^1^H NMR (500 MHz, CDCl_3_) δ 7.99 (d, *J* = 1.3 Hz, 1H), 7.87 (d, *J* = 8.9 Hz, 1H), 7.64 (ddd, *J* = 10.1, 8.6, 5.3 Hz, 3H), 7.58 (dd, *J* = 8.8, 1.8 Hz, 1H), 7.43–7.34 (m, 5H), 7.25–7.20 (m, 3H), 7.15 (t, *J* = 8.6 Hz, 2H), 6.98 (t, *J* = 7.4 Hz, 1H), 6.86 (d, *J* = 7.9 Hz, 2H). ^13^C NMR (126 MHz, CDCl_3_) δ 162.9 (d, *J* = 246.9 Hz), 158.7, 150.6, 137.8 (d, *J* = 2.9 Hz), 136.8, 135.4, 132.9, 131.2, 130.7, 129.9, 129.6 (d, *J* = 2.6 Hz), 129.0 (d, *J* = 8.1 Hz), 128.3, 127.6, 126.7, 126.1, 125.8, 122.5, 121.3, 117.8, 116.1, 115.8. HRMS (EI): m/z calculated for C_28_H_20_FO [M+H]^+^ = 391.1498, found 391.1508.

#### 6-(4-chlorophenyl)-2-phenoxy-1-phenylnaphthalene (2k)

The following compound was obtained according to the general procedure A, by using 6-(4-chlorophenyl) naphthalen-2-ol as starting material. The crude material was purified by flash column chromatography over silica gel with the (4% EtOAc/Hexane) system to afford the product **2k** (55 mg, 68%) as a white solid. m.p. = 138–140°C. R_*f*_ = 0.15 (5% EtOAc/Hexane). IR (neat) ν/cm^−1^ = 3,045, 1,586, 1,485, 1,224, 703. ^1^H NMR (500 MHz, CDCl_3_) δ 8.04 (d, *J* = 1.8 Hz, 1H), 7.90 (d, *J* = 8.9 Hz, 1H), 7.70 (d, *J* = 8.8 Hz, 1H), 7.65–7.60 (m, 3H), 7.47–7.42 (m, 4H), 7.41–7.38 (m, 3H), 7.28 (d, *J* = 8.9 Hz, 1H), 7.24 (d, *J* = 1.1 Hz, 1H), 7.24–7.22 (m, 1H), 7.01 (t, *J* = 7.4 Hz, 1H), 6.88 (dd, *J* = 8.6, 0.8 Hz, 2H). ^13^C NMR (126 MHz, CDCl_3_) δ 158.5, 150.7, 139.4, 136.5, 135.3, 133.6, 133.2, 131.7, 130.7, 129.9, 129.8, 129.6, 129.2, 128.6, 128.3, 127.6, 126.8, 125.94, 125.89, 122.5, 121.3, 117.8. HRMS (EI): m/z calculated for C_28_H_20_ClO [M+H]^+^ = 407.1203, found 407.1210.

#### 6-(3-chloro-4-fluorophenyl)-2-phenoxy-1- phenylnaphthalene (2l)

The following compound was obtained according to the general procedure A, by using 6-(4-chloro-3-fluorophenyl)naphthalen-2-ol and diphenyliodonium triflate as starting material. The crude material was purified by flash column chromatography over silica gel with the (4% EtOAc/Hexane) system to afford the product **2l** (53 mg, 68%) as a white solid. m.p. = 142–144°C. R_*f*_ = 0.55 (8% EtOAc/Hexane).

IR (neat) ν/cm^−1^ = 2,984, 1,560, 1,489, 1,309, 820. ^1^H NMR (500 MHz, CDCl_3_) δ 7.91 (d, *J* = 1.8 Hz, 1H), 7.80 (d, *J* = 8.9 Hz, 1H), 7.65–7.59 (m, 2H), 7.49–7.42 (m, 2H), 7.37–7.27 (m, 5H), 7.21–7.12 (m, 4H), 6.92 (t, *J* = 7.4 Hz, 1H), 6.80 (d, *J* = 7.8 Hz, 2H). ^13^C NMR (126 MHz, CDCl_3_) δ 158.8, 158.4, 156.8, 150.9, 138.2 (d, *J* = 15 Hz), 135.3 (d, *J* = 100 Hz), 133.2, 131.1, 130.7, 129.9, 129.7, 129.5, 128.3, 127.6, 127.1, 127.0, 126.94, 126.0, 125.7, 122.6, 121.6, 121.5, 117.9, 117.1 (d, *J* = 85 Hz). HRMS (EI): m/z calculated for C_28_H_19_ClFO [M+H]^+^ = 425.1108, found 425.1123.

### Diarylphenols of Scheme 3

#### 2-(4-chlorophenoxy)-1-(4-chlorophenyl)naphthalene (3a)

The following compound was obtained according to the general procedure A, by using 2-napthol and (4-chlorophenyl)(phenyl)-λ^3^-iodanyl trifluoromethanesulfonate as starting material. The crude material was purified by flash column chromatography over silica gel with the (2% EtOAc/Hexane) system to afford the product **3a** (92 mg, 73%) as a white solid. m.p. = 98–100°C. R_*f*_ = 0.56 (5% EtOAc/Hexane). IR (neat) ν/cm^−1^ = 2,919, 1,585, 1,485, 1,202, 821. ^1^H NMR (500 MHz, CDCl_3_) δ 7.81 (dd, *J* = 8.2, 3.7 Hz, 2H), 7.51 (d, *J* = 8.3 Hz, 1H), 7.42–7.30 (m, 4H), 7.22–7.17 (m, 2H), 7.16–7.09 (m, 3H), 6.69 (dd, *J* = 8.2, 3.7 Hz, 2H). ^13^C NMR (126 MHz, CDCl_3_) δ 157.1, 150.7, 133.7, 133.66, 133.64, 132.3, 131.8, 130.3, 129.6, 128.6, 128.3, 127.5, 127.0, 125.7, 125.4, 120.8, 118.7.

**Scheme 3:**
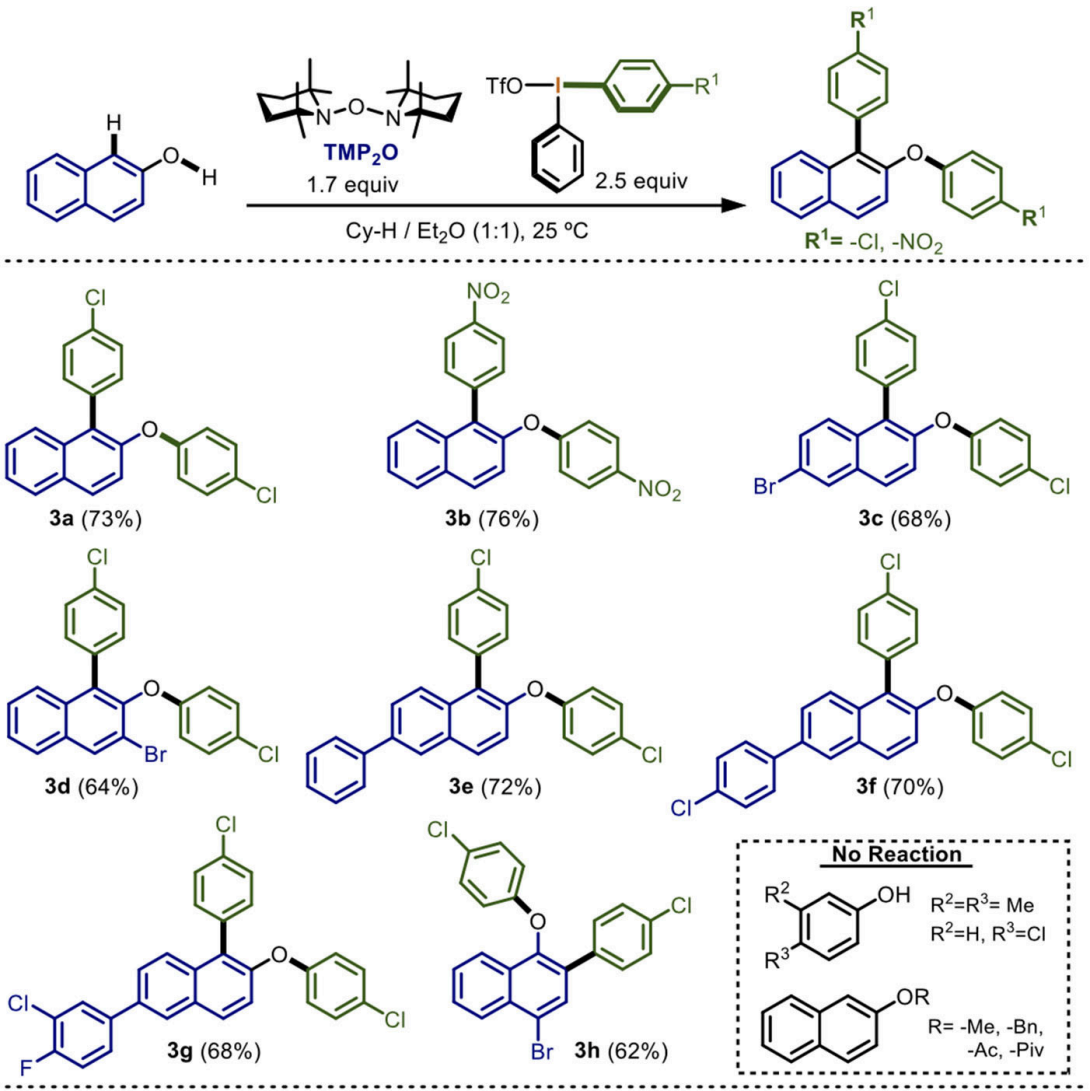
Scope of the electronic nature of the diaryliodonium(III) salt in the one-pot double arylation^*a,b*^.

HRMS (EI): m/z calculated for C_22_H_14_Cl_2_O [M]^+^ = 365.0500, found 365.0503.

#### 2-(4-nitrophenoxy)-1-(4-nitrophenyl)naphthalene (3b)

The following compound was obtained according to the general procedure A, by using 2-napthol and (4-nitrophenyl)(phenyl)-λ^3^-iodanyl trifluoromethanesulfonate as starting material. The crude material was purified by flash column chromatography over silica gel with the (8% EtOAc/Hexane) system to afford the product **3b** (99 mg, 76%) as a white solid. m.p. = 122–124°C. R_*f*_ = 0.36 (15% EtOAc/Hexane).

IR (neat) ν/cm^−1^ = 2,979, 1,484, 1,340, 1,235, 885. ^1^H NMR (500 MHz, CDCl_3_) δ 8.31–8.26 (m, 2H), 8.14–8.11 (m, 2H), 8.02 (d, *J* = 8.9 Hz, 1H), 7.97 (d, *J* = 8.2 Hz, 1H), 7.59–7.49 (m, 5H), 7.30 (d, *J* = 8.9 Hz, 1H), 6.90–6.84 (m, 2H). ^13^C NMR (126 MHz, CDCl_3_) δ 163.6, 148.5, 147.6, 142.8, 141.9, 133.0, 131.7, 131.6, 131.4, 128.7, 128.6, 127.8, 126.6, 126.1, 125.4, 123.7, 120.5, 116.6. HRMS (EI): m/z calculated for C_22_H_14_N_2_O_5_ [M]^+^= 386.0903, found 386.0910.

#### 6-bromo-2-(4-chlorophenoxy)-1-(4-chlorophenyl) naphthalene (3c)

The following compound was obtained according to the general procedure A, by using 6-bromonaphthalen-2-ol and (4-chlorophenyl)(phenyl)-λ^3^-iodanyl trifluoromethanesulfonate as starting material. The crude material was purified by flash column chromatography over silica gel with the (4% EtOAc/Hexane) system to afford the product **3c** (68 mg, 68%) as a white solid. m.p.=110–112°C. R_*f*_ = 0.55 (8% EtOAc/Hexane). IR (neat) ν/cm^−1^ = 2,979, 1,569, 1,459, 1,309, 840.^1^H NMR (500 MHz, CDCl_3_) δ 7.96 (d, *J* = 1.9 Hz, 1H), 7.69 (d, *J* = 8.9 Hz, 1H), 7.39 (dt, *J* = 19.1, 5.5 Hz, 2H), 7.34–7.31 (m, 2H), 7.20–7.10 (m, 5H), 6.70–6.66 (m, 2H). ^13^C NMR (126 MHz, CDCl_3_) δ 156.6, 150.3, 133.8, 133.4, 132.7, 132.5, 131.8, 130.16, 130.07, 129.6, 128.9, 128.8, 128.6, 127.7, 127.4, 121.3, 119.6, 118.7. HRMS (EI): m/z calculated for C_22_H_13_BrCl_2_O [M]^+^ = 441.9527, found 441.9523.

#### 3-bromo-2-(4-chlorophenoxy)-1-(4-chlorophenyl) naphthalene (3d)

The following compound was obtained according to the general procedure A, by using 3-bromonaphthalen-2-ol and (4-chlorophenyl)(phenyl)-λ^3^-iodanyl trifluoromethanesulfonate as starting material. The crude material was purified by flash column chromatography over silica gel with the (3% EtOAc/Hexane) system to afford the product **3d** (64 mg, 64%) as a white solid. m.p. = 130–132°C. R_*f*_ = 0.15 (6% EtOAc/Hexane). IR (neat) ν/cm^−1^ = 2,984, 1,560, 1,489, 1,309, 820. ^1^H NMR (500 MHz, CDCl_3_) δ 8.23 (s, 1H), 7.84 (d, *J* = 8.2 Hz, 1H), 7.55–7.42 (m, 3H), 7.35–7.32 (m, 2H), 7.18–7.15 (m, 2H), 7.12–7.08 (m, 2H), 6.58–6.54 (m, 2H). ^13^C NMR (126 MHz, CDCl_3_) δ 156.7, 146.0, 133.9, 132.9, 132.8, 132.7, 132.6, 132.5, 132.3, 131.5, 129.8, 128.4, 127.2, 127.1, 126.8, 126.6, 126.0, 116.6.

HRMS (EI): m/z calculated for C_22_H_13_BrCl_2_O [M]^+^ = 441.9527, found 441.9535.

#### 2-(4-chlorophenoxy)-1-(4-chlorophenyl)-6- phenylnaphthalene (3e)

The following compound was obtained according to the general procedure A, by using 6-phenylnaphthalen-2-ol and (4-chlorophenyl)(phenyl)-λ^3^-iodanyl trifluoromethanesulfonate as starting material. The crude material was purified by flash column chromatography over silica gel with the (3% EtOAc/Hexane) system to afford the product **3e** (85 mg, 72%) as a white solid. m.p. = 126–128°C. R_*f*_ = 0.38 (5% EtOAc/Hexane). IR (neat) ν/cm^−1^ = 2,922, 1,588, 1,465, 1,202, 828. ^1^H NMR (500 MHz, CDCl_3_) δ 8.01 (d, *J* = 1.5 Hz, 1H), 7.86 (d, *J* = 8.9 Hz, 1H), 7.65–7.61 (m, 3H), 7.58 (d, *J* = 8.8 Hz, 1H), 7.42 (t, *J* = 7.7 Hz, 2H), 7.36–7.30 (m, 3H), 7.25–7.22 (m, 2H), 7.19–7.16 (m, 1H), 7.14–7.10 (m, 2H), 6.72–6.68 (m, 2H). ^13^C NMR (126 MHz, CDCl_3_) δ 157.8, 150.5, 140.7, 138.2, 133.9, 133.7, 132.7, 132.3, 131.6, 130.7, 129.8, 129.1, 128.8, 128.6, 127.9, 127.8, 127.6, 126.7, 126.4, 126.1, 120.9, 118.7. HRMS (EI): m/z calculated for C_28_H_18_Cl_2_O [M+H]^+^ = 441.0813, found 441.0820.

#### 2-(4-chlorophenoxy)-1,6-bis(4-chlorophenyl) naphthalene (3f)

The following compound was obtained according to the general procedure A, by using 6-(4-chlorophenyl)naphthalen-2-ol and (4-chlorophenyl)(phenyl)-λ^3^-iodanyl trifluoromethanesulfo-nate as starting material. The crude material was purified by flash column chromatography over silica gel with the (6% EtOAc/Hexane) system to afford the product **3f** (65 mg, 70%) as a white solid. m.p. = 134–136°C. R_*f*_ = 0.45 (10% EtOAc/Hexane). IR (neat) ν/cm^−1^ = IR (neat) ν/cm^−1^ = 3,015, 1,460, 1,389, 1,309, 720. ^1^H NMR (500 MHz, CDCl_3_) δ 8.06 (s, 1H), 7.94 (d, *J* = 8.9 Hz, 1H), 7.66 (dt, *J* = 10.0, 8.7 Hz, 4H), 7.48 (d, *J* = 8.5 Hz, 2H), 7.44 (d, *J* = 8.4 Hz, 2H), 7.32 (d, *J* = 8.4 Hz, 2H), 7.27–7.25 (m, 1H), 7.24–7.20 (m, 2H), 6.82–6.78 (m, 2H). ^13^C NMR (126 MHz, CDCl_3_) δ 156.9, 150.5, 139.7, 136.9, 133.9, 133.8, 133.7, 132.9, 132.1, 131.7, 130.5, 129.7, 129.6, 128.9, 128.8, 128.7, 127.7, 126.8, 126.5, 126.4, 121.5, 118.9. HRMS (EI): m/z calculated for C_28_H_18_Cl_3_O [M+H]^+^ = 475.0423, found 475.0413.

#### 6-(3-chloro-4-fluorophenyl)-2-(4-chlorophenoxy)-1- (4-chlorophenyl)naphthalene (3g)

The following compound was obtained according to the general procedure A, by using 2-napthol and (4-chlorophenyl)(phenyl)-λ^3^-iodanyl trifluoromethanesulfonate as starting material. The crude material was purified by flash column chromatography over silica gel with the (6% EtOAc/Hexane) system to afford the product **3g** (59 mg, 66%) as a white solid. m.p.= 130–140°C.

R_*f*_ = 0.45 (15% EtOAc/Hexane). IR (neat) ν/cm^−1^ = 2,984, 1,560, 1,489, 1,309, 820. ^1^H NMR (500 MHz, CDCl_3_) δ 7.93 (d, *J* = 1.6 Hz, 1H), 7.84 (d, *J* = 8.9 Hz, 1H), 7.64 (dd, *J* = 7.0, 2.3 Hz, 1H), 7.58 (d, *J* = 8.8 Hz, 1H), 7.51 (dd, *J* = 8.8, 1.9 Hz, 1H), 7.46 (ddd, *J* = 8.5, 4.5, 2.3 Hz, 1H), 7.36–7.32 (m, 2H), 7.24–7.20 (m, 2H), 7.17 (dd, *J* = 9.7, 5.9 Hz, 2H), 7.15–7.10 (m, 2H), 6.72–6.68 (m, 2H). ^13^C NMR (126 MHz, CDCl_3_) δ 158.9, 156.9, 150.5, 138.0 (d, *J* = 20 Hz), 135.9, 133.8, 133.5, 133.0, 132.0, 131.3, 130.2, 129.7, 129.5, 128.8, 128.7, 127.7, 127.1, 127.0, 126.6, 126.1, 121.6 (d, *J* = 70 Hz), 121.1, 118.8, 117.1 (d, *J* = 20 Hz). HRMS (EI): m/z calculated for C_28_H_16_Cl_3_FO [M]^+^ = 492.0251 found 492.0247.

#### 4-bromo-1-(4-chlorophenoxy)-2-(4-chlorophenyl) naphthalene (3h)

The following compound was obtained according to the general procedure A, by using 4-bromonaphthalen-1-ol and (4-chlorophenyl)(phenyl)-λ^3^-iodanyl trifluoromethanesulfonate as starting material. The crude material was purified by flash column chromatography over silica gel with the (3% EtOAc/Hexane) system to afford the product **3h** (62 mg, 62%) as a white solid. m.p. = 98–100°C. R_*f*_ = 0.5 (5% EtOAc/Hexane). IR (neat) ν/cm^−1^ = 2,984, 1,560, 1,489, 1,309, 820. ^1^H NMR (500 MHz, CDCl_3_) δ 8.20 (d, *J* = 7.5 Hz, 1H), 7.87 (d, *J* = 7.5 Hz, 1H), 7.80 (s, 1H), 7.52 (t, *J* = 7.5 Hz, 1H), 7.40 (t, *J* = 7.5 Hz, 1H), 7.38 (d, *J* = 8.5 Hz, 2H), 7.24 (d, *J* = 8.5 Hz, 2H), 7.00(d, *J* = 8.5 Hz, 2H), 6.52 (d, *J* = 8.5 Hz, 2H). ^13^C NMR (126 MHz, CDCl_3_) δ 157.6, 146.8, 134.8, 134.2, 132.8, 131.6, 131.0, 130.6, 129.6, 129.3, 128.8, 128.3, 127.9, 127.7, 126.9, 123.7, 119.9, 116.8. HRMS (EI): m/z calculated for C_22_H_13_BrCl_2_O [M]^+^ = 441.9527, found 441.9523.

### Synthetic Utility of the Developed Procedure

#### Equation 1. 2-phenoxy-1,3-diphenylnaphthalene (4)

This compound was synthesized according to the general procedure for the Suzuki cross-coupling using **2c** as starting material and phenyl boronic acid. The purification was carried out using (3% EtOAc/Hexane) system to afford the product **4** in 62% of yield as a white solid. m.p. = 94–96°C. R_*f*_ = 0.45 (5% EtOAc/Hexane). IR (neat) ν/cm^−1^ = 3,046, 1,561, 1,474, 1,216, 925. ^1^H NMR (500 MHz, CDCl_3_) δ 8.05 (d, *J* = 1.3 Hz, 1H), 7.89 (d, *J* = 8.9 Hz, 1H), 7.71–7.64 (m, 3H), 7.46 (t, *J* = 7.7 Hz, 2H), 7.43–7.34 (m, 6H), 7.25–7.19 (m, 4H), 6.98 (t, *J* = 7.4 Hz, 1H), 6.86 (d, *J* = 7.8 Hz, 2H). ^13^C NMR (126 MHz, CDCl_3_) δ 158.9, 150.4, 140.8, 137.9, 135.4, 132.9, 131.4, 130.9, 129.8, 129.5(x2), 129.3, 128.9, 128.4, 127.9, 127.3, 126.5, 126.4, 125.8, 122.3, 121.7, 117.8. HRMS (EI): m/z calculated for C_28_H_21_O [M+H]^+^ = 373.1592, found 373.1594.

## Results and Discussion

We first targeted the selective *C*-arylation of phenols considering the typical behavior of Ar_2_IX.^12,13,22^ In our strategy, we envisaged the deprotonation of the hydroxyl group in the naphthol to form a bidentate anion for reacting with the Ar_2_IX. Then, the electron-poor aryl transfer from Ar_2_IX to the naphthol would take place. This way, ionic conditions in basic media for proton abstraction of the hydroxyl group in naphthol using different bases, solvent and temperatures were assayed. 2-Naphthol and the inexpensive diphenyliodonium(III) nitrate were used as a model system (Table 1).

**Table 1:** Optimization of the *C*- and *O*-arylation of 2-naphthol using diphenyliodonium(III) nitrate^a^.

**Entry**	**Ph_2_I(X), equiv**	**Base (equiv)**	**Solvent**	**T (°C)**	**t (h)**	**yield (%)^b^ 1 / 2 / 2a**
1	(NO_3_), 2.5	K_2_CO_3_, (1.5)	DMF	23	1.0	20/0/8
2	(NO_3_), 2.5	K_2_CO_3_, (1.5)	THF	23	1.5	35/0/25
3	(NO_3_), 2.5	K_2_CO_3_, (1.5)	THF	80	1.5	30/0/15
4	(NO_3_), 2.5	KHCO_3_, (1.5)	DMF	23	2.0	20/0/15
5	(NO_3_), 2.5	KHCO_3_, (1.5)	THF	23	1.0	12/0/18
6^c^	(NO_3_), 3.5	*^t^*BuOK, (2.5)	THF	23	0.5	35/10/28
7^c^	(NO_3_), 3.5	*^t^*BuOK, (2.5)	DCE	23	0.5	25/0/18
8^c^	(NO_3_), 3.5	*^t^*BuOK, (2.5)	MeCN	23	2.0	20/0/16
9^c^	(NO_3_), 3.5	*^t^*BuOK, (2.5)	Tol	23	2.0	20/0/14
10^c^	(NO_3_), 3.5	*^t^*BuOK, (2.5)	DMF	23	1.5	25/0/18
11^c^^,^ ^d^	(NO_3_), 3.5	*^t^*BuOK, (2.5)	THF	130	0.5	37/13/24
12^c^^,^ ^d^	(NO_3_), 3.5	*^t^*BuOK, (2.5)	DME	130	0.5	30/15/26
13	(NO_3_), 3.5	Cs_2_CO_3_, (2.5)	THF	23	0.5	30/0/15
14	(NO_3_), 3.5	NaH, (2.5)	THF	23	2.0	20/0/15
15	(NO_3_), 2.5	–	TMEDA	23	0.5	n. r.
16^e^^,^ ^f^	(NO_3_), 2.5	TMP_2_O, (1.2)	Cy-H/Et_2_O	23	5.0	0/0/59
17^e^^,^ ^f^	(NO_3_), 2.5	TMP_2_O, (1.5)	Cy-H/Et_2_O	23	5.0	0/0/65
18^f^	(NO_3_), 2.5	TMP_2_O, (1.7)	Cy-H/Et_2_O	23	3.0	0/0/70
19^f^	***(OTf), 2.5***	***TMP**_**2**_**O, (1.7)***	***Cy-H/Et**_**2**_**O***	***23***	***3.0***	***0/0/74***
20^f^	(OTs), 2.5	TMP_2_O, (1.7)	Cy-H/Et_2_O	23	3.0	0/0/65
21^f^	(PF_6_), 2.5	TMP_2_O, (1.7)	Cy-H/Et_2_O	23	3.0	0/0/45
22^f^	(NO_3_), 2.5	LiOH, (1.7)	Cy-H/Et_2_O	23	3.0	18/0/54
23^f^	(NO_3_), 2.5	NaOH, (1.7)	Cy-H/Et_2_O	23	3.0	47/0/10
24^f^^,^ ^g^	(OTf), 2.5	–	Cy-H/Et_2_O	23	12	5/0/10

a *Reaction conditions: 2-naphthol (0.25 mmol), solvent (0.1 M), open flask*.

b *Isolated yields*.

c *Complex reaction mixture obtained*.

d *Microwave-assisted reaction in sealed tube at 1,500 W*.

e *3–7% of remaining starting material was recovered*.

f *A 1:1 mixture of solvents was used*.

g *Yield is the average of three runs; 93% of starting material recovered. TMP_2_O = [1,1′-oxybis(2,2,6,6-tetramethylpiperidine)]. TMEDA = N,N,N′,N′-tetramethyleth-ylenediamine. n.r. = no reaction observed*.

Initial attempts to induce the arylation of 2-naphthol were carried out using excess of diphenyliodonium nitrate and potassium carbonate in dimethylformamide since essentially no reaction was found with one equivalent.

The best reaction progress was found after 1 h at room temperature, obtaining 20% of *C*-arylation and 8% of the doubly arylated product (*C*-/*O*-arylation) (entry 1). A great amount of unreacted starting material was observed in this assay even after 12 h of reaction. When using THF as solvent, a considerable increase in the reaction yield was obtained with the same product ratio (entry 2). Also, large amounts of unreacted starting material were observed even by heating at 80°C; in this case a lower yield was obtained (entry 3). The change of base to bicarbonate did not improve the yields (entries 4 and 5). These attempts to get the single *C*-arylated product **1** resulted in a poor conversion of the starting material. Thus, we considered the use of a stronger base such as potassium *tert*-butoxide (entries 6–12). After an extensive experimentation with different solvents and temperatures, the complete consumption of the starting material was achieved. However, it resulted in a complex mixture of products. The single *C*-arylation product was identified and isolated from the crude when THF or DME were tested as solvents (entries 6, 11, and 12). Nevertheless, we were unable to get selectivity for any singly arylated product. We tried with cesium carbonate (entry 13) but, again, unreacted starting material and unselective mixtures of arylated products were observed. The base reactivity was scaled up and sodium hydride was assayed as a stronger reagent (entry 14). However, a lower conversion was obtained. Another experiment was performed using TMEDA as solvent and base, but no reaction was observed in this case (entry 15).

At this point, our hypothesis for the poor reactivity of the naphthyl anion formed after deprotonation was attributed mainly to the use of a not enough strong base, which may generate the proper chemical environment of reactivity, and the inappropriate choice of the reaction solvent. In consequence, we decided to use TMP-Li (lithium 2,2,6,6-tetramethylpiperidin-1-ide) as base and cyclohexane/diethyl ether (1:1) as reaction solvent, according to the related arylation procedure of Daugulis (Truong and Daugulis, 2012).

Therefore, the synthesis of the TMP-Li was started from the deprotonation of TMP-H with *n*-BuLi. During the work-up of this synthesis, *the direct evaporation of the crude reaction* lead to the formation of a brown, *non-pyrophoric* and *air stable* solid identified by HRMS as TMP_2_O. This solid was tested in the following experiment. By using 1.2 equiv of TMP_2_O and 2.5 equiv of diphenyliodonium nitrate at room temperature, the selective double arylated product (*C*-/*O*-arylation) was surprisingly obtained in 59% yield and 7% of starting material was recovered after 5 h (entry 16). Moreover, 1.5 equiv of TMP_2_O produced the diarylated product in 65% yield and, again, 3% of starting material was recovered (entry 17). Finally, the use of 1.7 equiv of TMP_2_O yielded the double arylated naphthol in 70% after 3 h. However, no remaining starting material was observed (entry 18). Remarkably, neither the single *O*- nor *C*-arylated naphthalenes were observed under these new base-free conditions.

We became interested in this doubly arylated product after considering that: (1) it was selectively formed as the sole arylation product, (2) new arylation reactivity was found by using TMP_2_O and (3) this reaction proceeded under neutral and base-free conditions in contrast with the strongly basic conditions previously attempted. Thus, we considered this procedure as our first approach toward the selective *C*-arylation.

We continued the optimization to explore about the effect of the anion fragment within the Ar_2_IX in the reaction. Triflate, tosylate and hexafluorophosphate gave exclusively the double arylated product in 74, 65, and 45% yield, respectively (entries 19–21). We identified the Ar_2_IX with the triflate as anion as the best arylating reagent for this protocol. Next, lithium and sodium hydroxide were tested as bases, since they could have formed during the TMP_2_O preparation and plausibly participated in the arylation. However, the typical mixture of *C*- and *C*-/*O*-arylation was obtained with those bases (entries 22 and 23). An inverse ratio in the product mixture was observed when Li^+^ or Na^+^ cations were present, highlighting their strong influence in the reaction.

For completing the optimization, a control experiment was carried out in absence of TMP_2_O. Therefore, 5% of the *O*-arylation and 93% of starting 2-napthol was recovered after 12 h (entry 24). This essay confirmed the need of TMP_2_O for the reaction to proceed.

With the optimized conditions identified in the entry 19, we continue to explore the scope of the protocol (Scheme 2).

Several substituted naphthols were assayed to determine the scope of the reaction when varying its electronic nature. Electron-neutral 2- and 1-naphthol underwent double phenylation in 74 and 47% yield (**2a** and **2b**). The lower yield for 1-napthol was attributed to the greater number of reactive centers, which allowed more side-reactions. On the other hand, 2-naphthol derivatives containing electron-attracting groups such as bromine at the positions 3 and 6, gave 72 and 74% yields, respectively (**2d** and **2e**). In contrast, with the 4-bromo-1-naphthol a lower yield (56%) was obtained (**2c**). The steric hindrance of bromine did not seem to affect the reaction. Additionally, the electron-donating groups 4-tolyl, methoxy, phenyl, and 2-naphthyl in the naphthol moiety were successfully tested. In these cases, slightly lower yields ranging from 64 to 66% were found (**2f** to **2i**). These results may indicate the formation of a less-stable intermediate species during the arylation which was reflected in decreasing yields. We also used 2-naphthol derivatives with electron-attracting groups, such as 4-fluorophenyl, 4-chlorophenyl, and 3-chloro-4-fluorophenyl, leading to the formation of the doubly phenylated products in 71 and 68% yields, respectively (**2j** to **2l**). In general, very similar yields were obtained for various substrates in this one-pot double phenylation.

The previous set of experiments described the scope of the electronic nature of the 2-naphthol nucleus. The next step was to determine the functional-group tolerance of the reaction in the diaryliodonium(III) salt (Scheme 3).

Non-symmetrical diaryliodonium(III) salts containing electron-attracting groups were mainly tried. 2-Naphthol was subjected to our optimized reaction conditions using a diaryliodonium(III) salt which contains a chlorine atom in one of the aromatic rings. Gratifyingly, the double arylation product **3a** was isolated in 73% yield. Remarkably, only the electron-poor aryl was chemoselectively transferred following the Beringer and DiMagno observations (Beringer and Mausner, 1958; Beringer and Chang, 1971; Wang et al., 2010; Malmgren et al., 2013; Stuart, 2017). When nitro-containing diaryliodonium(III) salt was used, a 76% yield of **3b** was obtained, also by the selective transfer of the electron-poor aryl. Derivatives of 2-napthol having bromine atoms successfully reacted leading to the formation of **3c** and **3d** in 68 and 64% yields, respectively. Other derivatives substituted with phenyl, 4-chlorophenyl and 3-chloro-4-fluorophenyl groups proceeded in yields ranging from 68 to 72% (**3e** to **3g**). Additionally, by testing 4-bromo-1-naphthol with the chlorine-containing diaryliodonium(III) salt, the corresponding double arylation product was achieved in 62% yield (**3h**).

Finally, some mono-annular phenols assays did not display observable reaction, presumably due to their higher REDOX potential (2.1 eV) compared with naphthols (1.87 eV) (Brodwel and Cheng, 1991; Lee et al., 2014; Kang et al., 2017). In these cases, a stronger radical initiator different to TMP_2_O or higher temperatures must be employed. On the other hand, the methyl or benzyl ethers as well as the acetyl and pivaloyl esters of the 2-napthol did not produce any reaction. This observation strongly suggested that *C*-arylation on the naphthol takes place prior to *O-*arylation. Other Ar_2_IX containing electron-donating groups showed very low reactivity and were ruled out of the scope of this first report.

The synthetic utility of our procedure was demonstrated by synthesizing a highly substituted 2-naphthol derivative **4** (Equation 1).


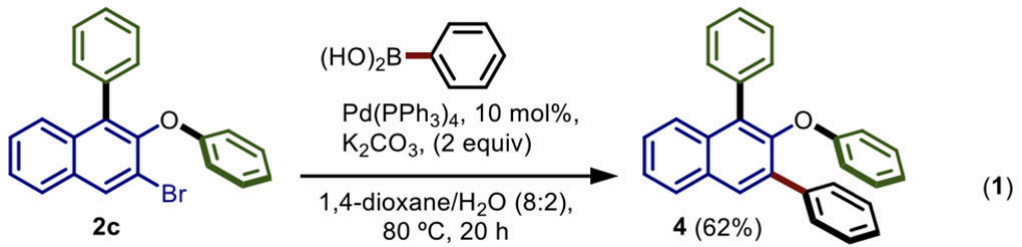


The Suzuki cross-coupling of **2c** with phenylboronic acid gave rise to the sterically hindered tris-arylated species **4** in 62% yield.

Intrigued by the observed new reactivity of this one-pot protocol of double arylation, we investigated a plausible reaction mechanism both theoretical and experimentally.

**Scheme 4:**
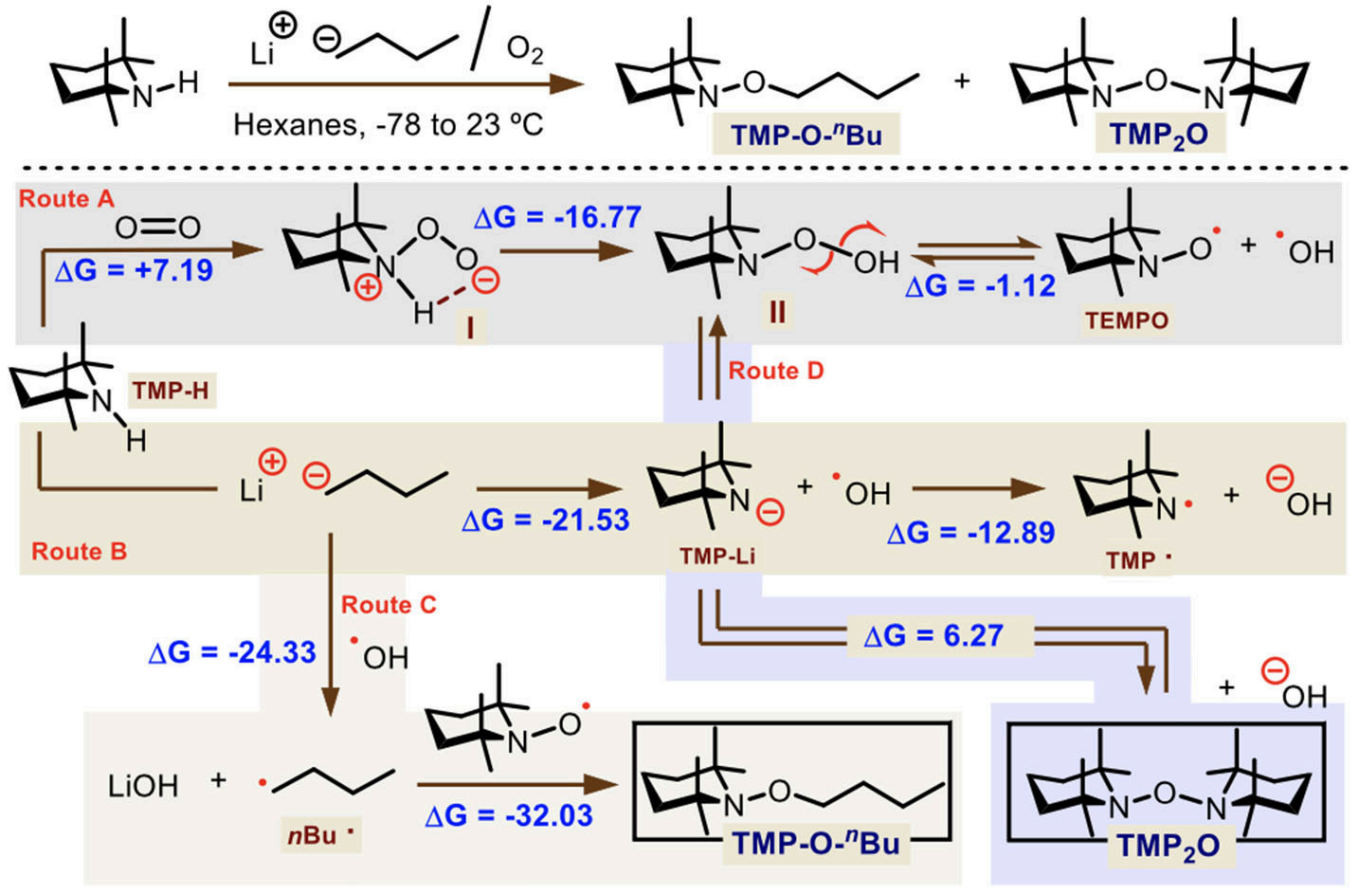
Theoretical study of the mechanism for the TMP-O-^*n*^Bu and TMP_2_O formation. Gibbs free-energies are shown in kcal·mol^−1^.

We first analyzed the brown, non-pyrophoric and air stable solid obtained from the reaction between TMP-H and *n-*BuLi which allowed the formation of TMP_2_O. This solid was identified by HRMS as the mixture of two main compounds: the TMP-O-^*n*^Bu [M+H]^+^ = 214.2171 and TMP_2_O [M+H]^+^ = 297.2906 in 9:1 ratio. Therefore, DFT calculations at the (SMD:diethylether)ω-B97XD/6-311G(d) level were performed for measuring reaction energies and postulate a plausible reaction route (Scheme 4).

We suggest that four reaction pathways operate at the same time to produce both radicals in solution. TMP-H can either react with molecular oxygen or *n*-BuLi. In the case of O_2_ (route A), this is inserted into the nitrogen to get intermediate **I** which readily tautomerizes to **II**. Transition states were located 8.67 and 21.13 kcal·mol^−1^ from reactants (see Supporting Information), for each reaction step. Then, peroxide **II** is in equilibrium with TEMPO and ^·^OH radicals, given the energy difference between both states (Δ${\text{G}}_{\text{R}}^{0}$ = −1.12 kcal·mol^−1^). On the other hand (route B), TMP-H combines with *n*-BuLi producing TMP-Li and *n*-butane (Δ${\text{G}}_{\text{R}}^{0}$ = −21.53 kcal·mol^−1^). The TMP-Li anion can transfer an electron to the ^·^OH radical leading to TMP· radical and OH^−^ spontaneously (Δ${\text{G}}_{\text{R}}^{0}$ = −12.89 kcal·mol^−1^). Both TEMPO and TMP· radicals do not combine in solution to derive TMP_2_O according to the calculated energy (Δ${\text{G}}_{\text{R}}^{0}$ = +20.27 kcal·mol^−1^). However, because of the harsh conditions carried out during the HRMS technique, it is very possible that the TMP_2_O can be detected as a single molecule. Also, an aside reaction (route C) comes from the possibility that ·OH radical absorbs one electron not only from TMP-Li anion but from the initial Bu^−^ of *n*-BuLi. This reaction is more exergonic (Δ${\text{G}}_{\text{R}}^{0}$ = −24.33 kcal·mol^−1^) than the previous ones. And the reaction between TEMPO and Bu^·^ radicals that gives TMP-O-^*n*^Bu is also more favorable (Δ${\text{G}}_{\text{R}}^{0}$ = −32.03 kcal·mol^−1^). Thus, this product is mostly obtained than TEMPO and TMP· radicals, according to our calculations. Finally, one more possibility (route D) may come from the reaction among peroxide **II** and TMP-Li to form TMP_2_O and OH^−^ in solid state. This is less exergonic than other pathways (Δ${\text{G}}_{\text{R}}^{0}$ = +6.27 kcal·mol^−1^), but not enough to keep the molecular TMP_2_O in solution, which homolyze readily.

**Scheme 5:**
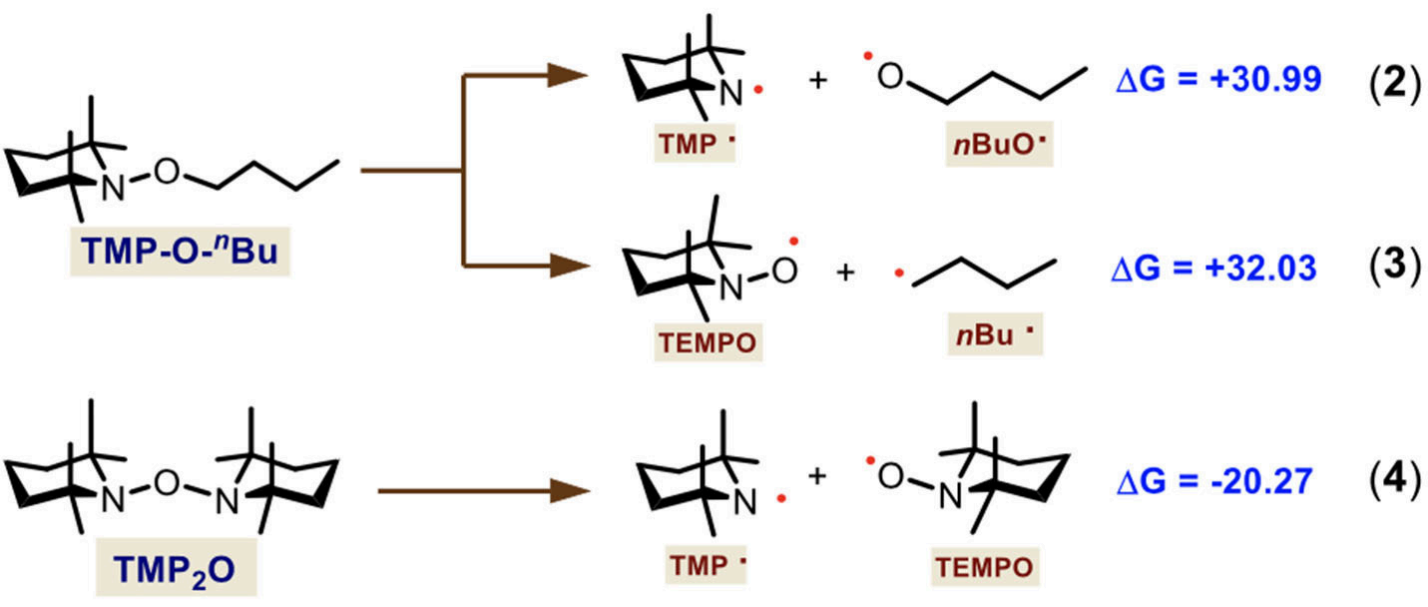
Theoretical study of the TMP-O-^*n*^Bu and TMP_2_O homolytic fragmentation. Gibbs free-energies are shown in kcal·mol^−1^.

According to these results and considering that TMP-O-^*n*^Bu and TMP_2_O are the main species observed by HRMS, we hypothesized that a radical mechanism could be operating since several different ionic conditions previously tested just gave mixtures of non-selective arylation products (Table 1, entries 1–15, 22, and 23). Under this hypothesis, we also calculated two homolytic fragmentations for TMP-O-^*n*^Bu as well as for TMP_2_O. This way, we demonstrate that the radical source comes from TMP_2_O and the other product is not involved in the subsequent reactions (Scheme 5).

We first calculated two possible fragmentations for TMP-O-^*n*^Bu. These showed thermodinamically both nonfavorable processes which gave high-in-energy species, the TMP–O^*n*^Bu (Δ${\text{G}}_{\text{R}}^{0}$ = +30.99 kcal·mol^−1^) (Equation 2) and the TMPO–^*n*^Bu (Δ${\text{G}}_{\text{R}}^{0}$ = +32.03 kcal·mol^−1^) (Equation 3) homolysis. Thus, we identified that TMP_2_O is the active initiator species, providing the TMP· and TEMPO radicals (Equation 4). For this calculated fragmentation, a very favorable process was found (Δ${\text{G}}_{\text{R}}^{0}$ = −20.27 kcal·mol^−1^). We also performed other theoretical calculations for reactions where these compounds act as bases and deprotonate the 2-naphthol, which gave high exergonic free energies (see Scheme S1 of Supporting Information). Thus, an ionic route was ruled out.

**Scheme 6:**
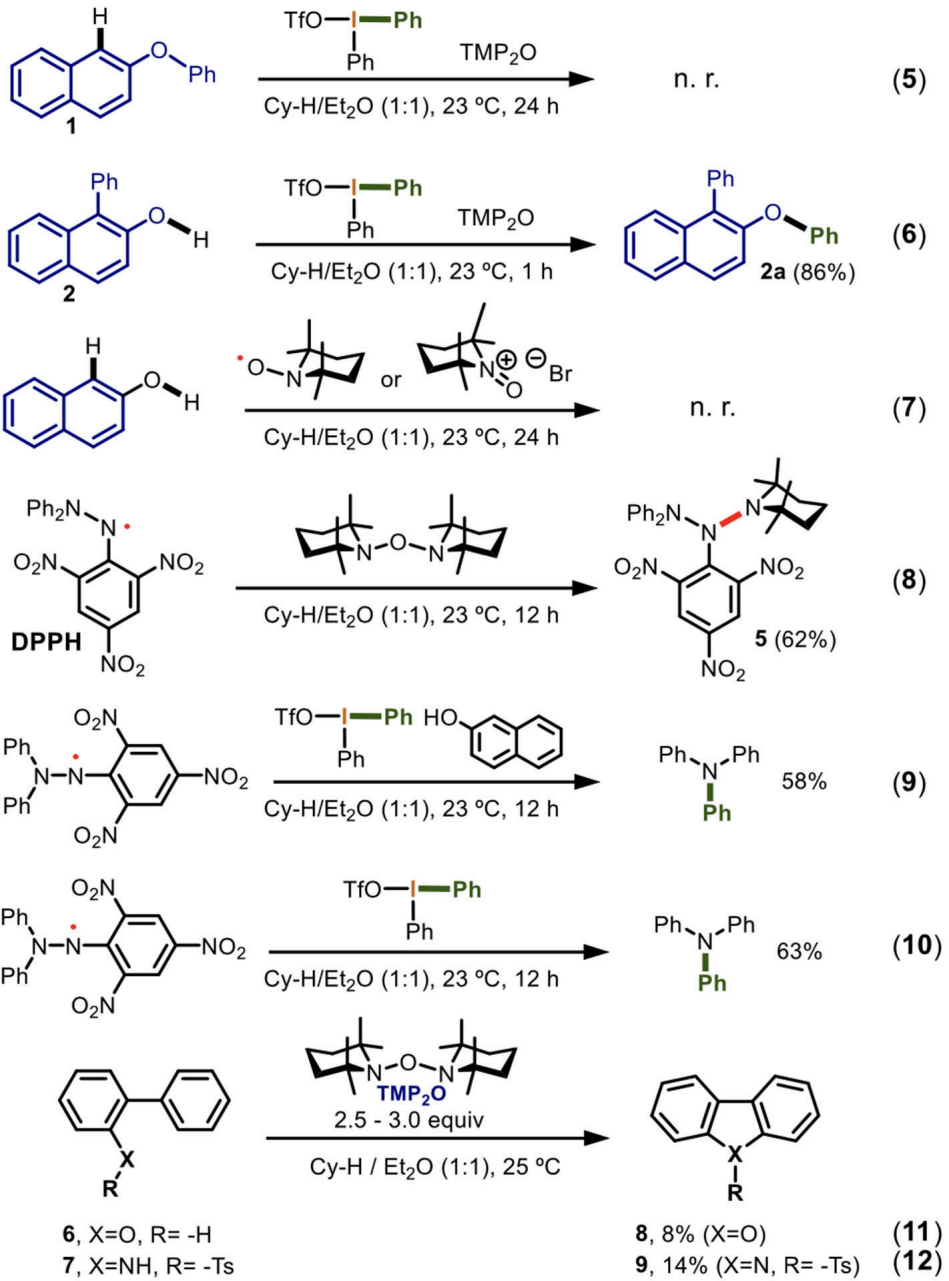
Experimental mechanistic investigation of the one-pot double arylation of naphthols mediated by TMP_2_O/DIS.

With these preliminary conclusions, we had enough information to experimentally elucidate a reaction mechanism for the observed double arylation. In consequence, a series of reactions were carried out (Scheme 6).

Different experiments were carried out to determine how the C-1 and the oxygen of the 2-naphthol derivatives were activated by TMP_2_O. The mechanistic investigation started by the elucidation of the reaction center of the first arylation. Thus, when the optimized conditions (Table 1, entry 19) were applied to the 2-phenoxynaphthalene, no reaction was observed (Equation 5). However, 1-phenyl-2-naphthol led to the *O*-arylation product **2a** in 86% yield (Equation 6). These two experiments indicated that for this developed procedure in the 2-naphthol derivatives the first arylation occurred at the carbon followed by the arylation at the oxygen. The result is in line with the innate reactivity of 2-naphthol.

According to Equation 4, the homolytic fragmentation of TMP_2_O is a very favored process and takes place spontaneously. Therefore, we experimentally confirmed the theoretical results on the generation of the TMP· and TEMPO radicals and its *N*-oxide with 2-naphthol as a model. No reaction was found for last two compounds (Equation 7). Also, to identify the TMP· radical, we designed a trapping experiment using the persistent radical DPPH (2,2-diphenyl-1-picrylhydrazyl). To our delight, we formed **5** in 62% yield (Equation 8). The result of this experiment can be explained exclusively via a radical pathway, thus unequivocally it demonstrated the homolytic fragmentation of TMP_2_O and established it as a precursor of TMP· and TEMPO radicals. It also showed that the TMP· radical reacted faster than TEMPO, consistent with its greater reactivity.

We next sought to study the reaction of diaryliodonium(III) salts reaction with the *C*-centered radical of the 2-naphthol. We attempted to generate this naphthyl radical using DPPH instead of the TMP· under the optimized conditions. In this experiment, the *selective reaction* of the *N*-centered radical of DPPH with the hypervalent bond of the Ph_2_IOTf instead of the *O-*centered radical formation in the 2-naphthol via HAT took place. This reaction produced triphenylamine in 58% by transferring one aryl group from Ph_2_IOTf and fragmentating the picryl moiety (Equation 9). Thus, the DPPH· radical directly reacted with the Ph_2_IOTf and not with 2-naphthol. To confirm this reactivity, the same reaction was carried out in absence 2-naphthol. Triphenylamine was again obtained in 63% yield (Equation 10). These two experiments (Equations 9 and 10) demonstrated that the diaryliodonium(III) salts reacted slowly at its hypervalent bond with persistent radicals such as DPPH. This observed reactivity represents a new activation mode of the Ph_2_IOTf. Additional experiments are currently ongoing to determine if there is a pattern of chemo-selectivity in the reaction of transient and persistent radicals with naphthols or Ar_2_IX.

Complementary studies to determine the radical precursor-nature of TMP_2_O were conducted. Therefore, the radical cyclization of 2-phenylphenol **6** using 2.5 equiv of TMP_2_O gave rise to the dibenzo[*b,d*]furan **8** in 8% of yield (Equation 11). Also, by using 3 equiv of TMP_2_O, the radical cyclization of the *N*-tosyl-2-phenylaniline lead to the formation of 9-tosyl-9*H*-carbazole **9** in 14% of yield (Equation 12). The experiments are in line with a radical cyclization. However, the observed low yields are attributed in one side to the non-efficient radical formation in **6** and **8**. This low reactivity of TMP_2_O for mono-annular phenols was previously observed (Scheme 3). On the other hand, there is a poor radical stabilization through the contiguous phenyl ring where the cyclization takes place, after the *O*- and *N*-centered radical formation occurs reacting with TMP_2_O.

**Scheme 7:**
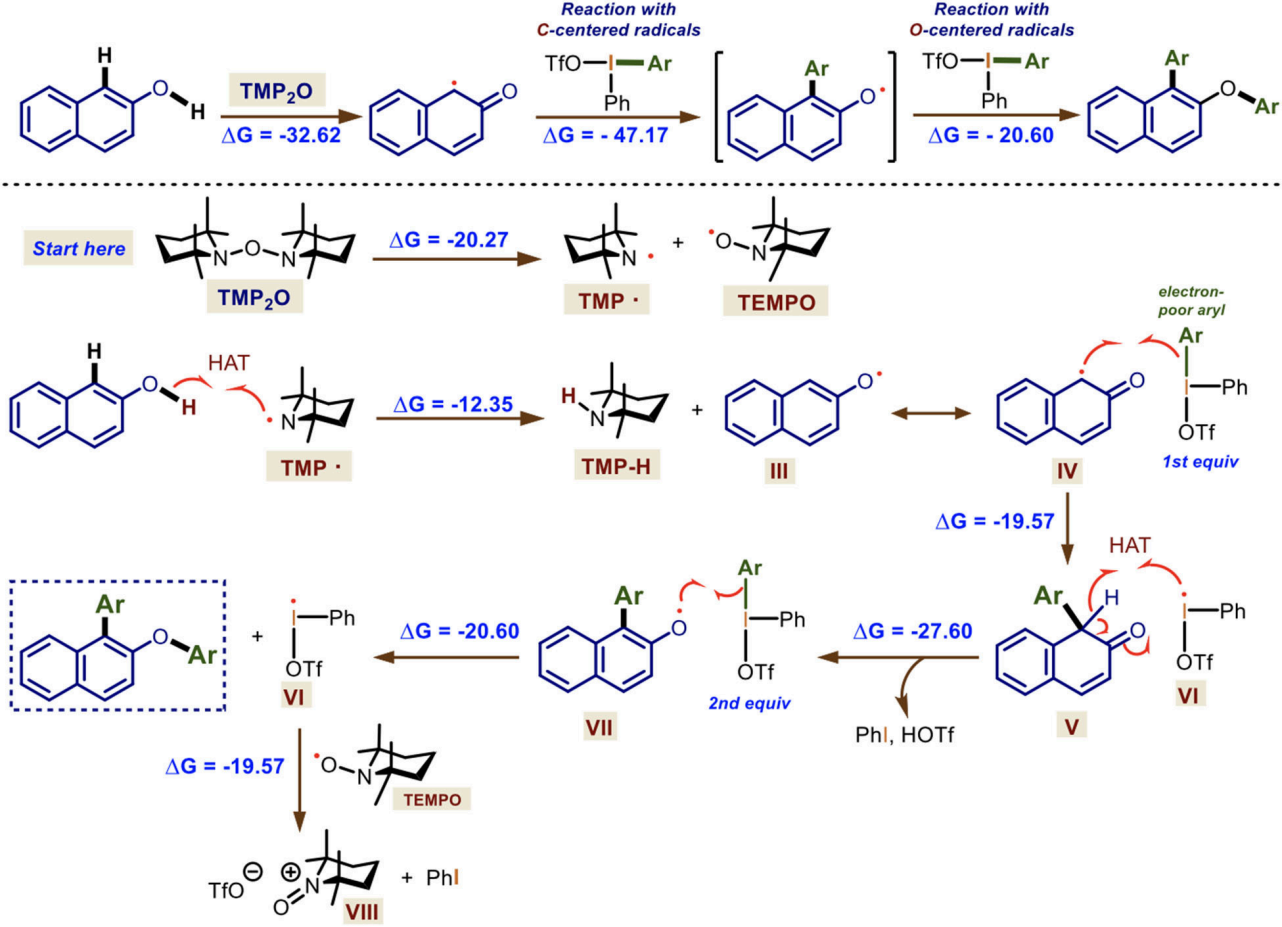
Mechanistic rationale for the one-pot double arylation mediated by TMP_2_O/DIS. Gibbs free-energies are shown in kcal·mol^−1^.

Finally, considering the overall spectroscopic, theoretical, and experimental mechanistic studies, additional DFT calculations at the same level of theory were carried out to support the plausible reaction mechanism of the double arylation of naphthols mediated by TMP_2_O and Ar_2_IOTf which is outlined in Scheme 7.

The reaction starts with the homolysis of TMP_2_O generating the transient TMP· radical, which orthogonally reacts faster via HAT with the 2-naphthol derivatives (Δ${\text{G}}_{\text{R}}^{0}$ = −12.35 kcal·mol^−1^) forming an *O*-centered radical **III** in resonance with its *C*-centered radical **IV**. This reacts with the hypervalent bond of the first equivalent of the diaryliodonium(III) salt gave rise to the arylated, non-aromatic **V** and the iodanyl radical **VI** (Δ${\text{G}}_{\text{R}}^{0}$ = −19.57 kcal·mol^−1^). The following HAT reaction promoted by this *I*-centered radical leads to the formation of a new aromatic *O*-centered radical **VII**, releasing triflic acid and iodobenzene (Δ${\text{G}}_{\text{R}}^{0}$ = −27.60 kcal·mol^−1^). Then, **VII** reacts with a second equivalent of the diaryliodonium(III) salt at the hypervalent bond, yielding the doubly arylated naphthol and a second molecule of **VI** (Δ${\text{G}}_{\text{R}}^{0}$ = −20.60 kcal·mol^−1^). The final reaction of **VI** with the persistent radical TEMPO forms the *N*-oxide triflate **VIII** of TMP· and releases iodobenzene (Δ${\text{G}}_{\text{R}}^{0}$ = −19.57 kcal·mol^−1^).

Additional synthetic applications of this new radical precursor TMP_2_O, as well as the new reactivity displayed by the Ar_2_IOTf, are currently being explored and developed in our laboratory.

## Conclusions

In summary, we demonstrated that the radical precursor TMP_2_O spontaneously undergoes homolytic fragmentation in solution and generates the transient *N-*centered radical of tetramethylpiperidinyl (TMP·) as well as the persistent radical TEMPO. The TMP· radical reacts with 2-naphthol derivatives, giving rise to the formation of an *O*-centered radical via HAT, in resonance with its corresponding *C*-centered radical. These *C*- and *O*-centered naphthyl radicals sequentially react with diaryliodonium(III) salts at its more electron-deficient hypervalent bond, transferring chemoselectively the more electron-poor aryl group. This observed reactivity constitutes a new activation mode of the diaryliodonium salts, which was used for developing the first one-pot double arylation procedure of naphthols via the sequential ${\text{C}}_{\text{sp}}^{2}$-${\text{C}}_{\text{sp}}^{2}$/O-${\text{C}}_{\text{sp}}^{2}$ bond formation. Spectroscopic, theoretical and experimental mechanistic studies supported and revealed a complete panorama of the reaction pathway. Finally, this novel protocol was conducted under neutral and base-free, room temperature and operationally simple conditions.

## Data Availability Statement

All datasets generated for this study are included in the article/Supplementary Material.

## Author Contributions

YS: full experimental part. KW: TMP_2_O and TMP-O-^*n*^Bu analysis by HRMS and results discussion of this section. DT-G: radical calculations of Schemes 4, 5. RO-A: first draft writing and initial discussion. JJ-H: writing of full theoretical calculations part. CS-A: full organization, supervision, writing of final version of manuscript, and submission. All authors contributed to the article and approved the submitted version.

## Conflict of Interest

The authors declare that the research was conducted in the absence of any commercial or financial relationships that could be construed as a potential conflict of interest.
