# Supplementary material for: The Diaryliodonium(III) Salts Reaction With Free-Radicals Enables One-Pot Double Arylation of Naphthols
Source: Front Chem. 2020 Oct 15;8:563470. doi: 10.3389/fchem.2020.563470 (PMC7593783; doi:10.3389/fchem.2020.563470)
Supplement: Supplementary file 1 [file Data_Sheet_1.PDF]

The Diaryliodonium(III) Salts Reaction with Free-Radicals Enables One-Pot Double Arylation of Naphthols

Yuvraj Satkar,[a] Kazimirez Wröbel,[a] Daniel E. Trujillo-González,[a] J. Oscar C. Jimenez-Halla,*,[a] Rafael Ortiz-Alvarado,*,[b] and César R. Solorio-Alvarado*,[a]

[a] Universidad de Guanajuato, Campus Guanajuato, División de Ciencias Naturales y Exactas, Departamento de Química, Noria Alta S/N, 36050, Guanajuato, Guanajuato. México.

[b] Facultad de Químicofarmacobiología, Universidad Michoacana de San Nicolás de Hidalgo, Tzintzuntzan 173, Col. Matamoros 58240, Morelia Michoacán México.

*Corresponding author: csolorio@ugto.mx, jimenez@ugto.mx, rafael.ortiz@umich.mx

TABLE OF CONTENTS

	Page
**1. Characterization of the "*brown and air stable solid*" by HRMS**	**S4**
A. Ratio of TMP-O-^{n}Bu and TMP_2O	**S5**
B. HRMS (MS-MS) of TMP-O-^{n}Bu	**S5**
C. HRMS (MS-MS) of TMP_2O	**S6**
**2. Mechanistic studies of the double arylation using the system TMP_2O/ DIS**	**S20**
**3. DFT calculations**	**S26**
**4. ^1H and ^{13}C NMR spectra for compounds 1-21**	**S45**
**5. References**	**S76**

1. Characterization of the "*brown and air stable solid*" by HRMS

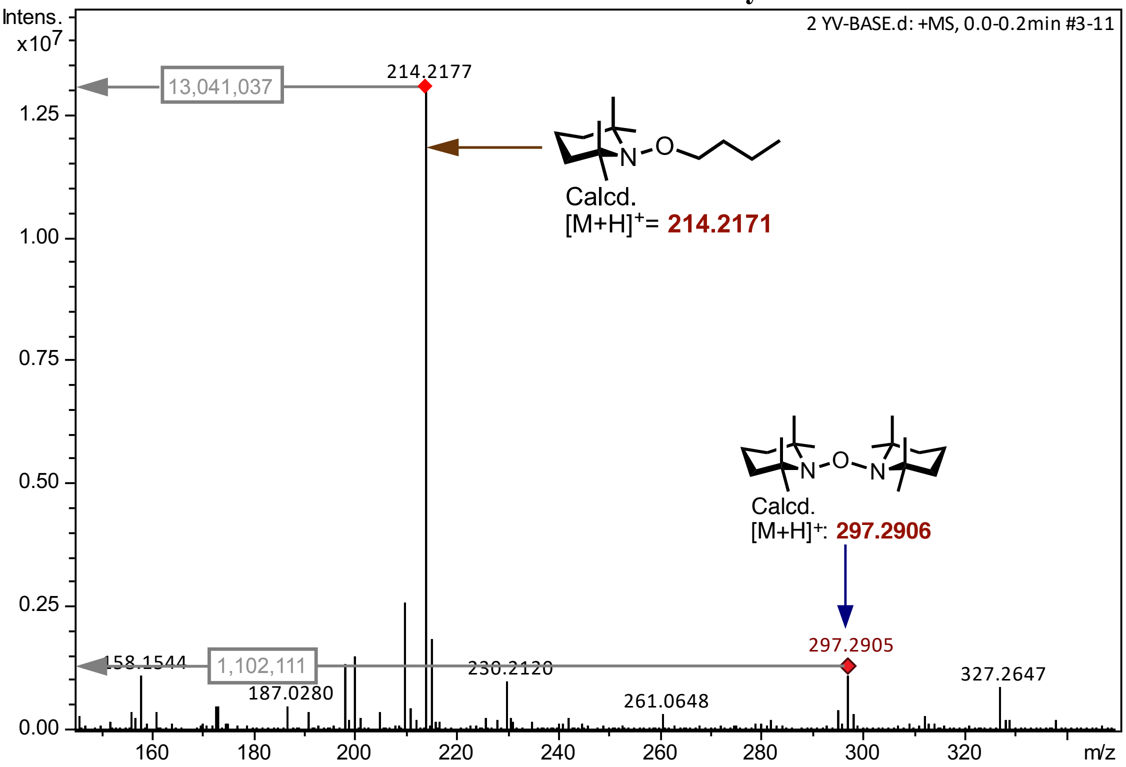

Under the high-resolution mass analysis of the obtained "brown solid" we were able to identify a mixture of two main compounds as well as their ratio in the mixture:

1) TMP-O-^{n}Bu $[M+H]^+$= 214.21717 and

2) TMP_2O $[M+H]^+$= 297.2906 in an approximately (9:1) ratio.

According to the intensity of the obtained HRMS chromatogram we determined that every 100 mg of this mixture contains 8 mg of TMP_2O. (See gray arrows)

A. Ratio of TMP-O-^{n}Bu and TMP_2O

I. Amount of TMP-O-^{n}Bu = [13041037 / (13041037 + 1102111)]*100 = 92.2%

II. Amount of **TMP_2O** = [1102111 /(13041037 + 1102111)]*100 = **7.8%**

The subsequent MS-MS analysis of TMP-O-^{n}Bu as well as of TMP_2O allowed the observation of different representative fragments.

B. HRMS (MS-MS) of TMP-O-^{n}Bu

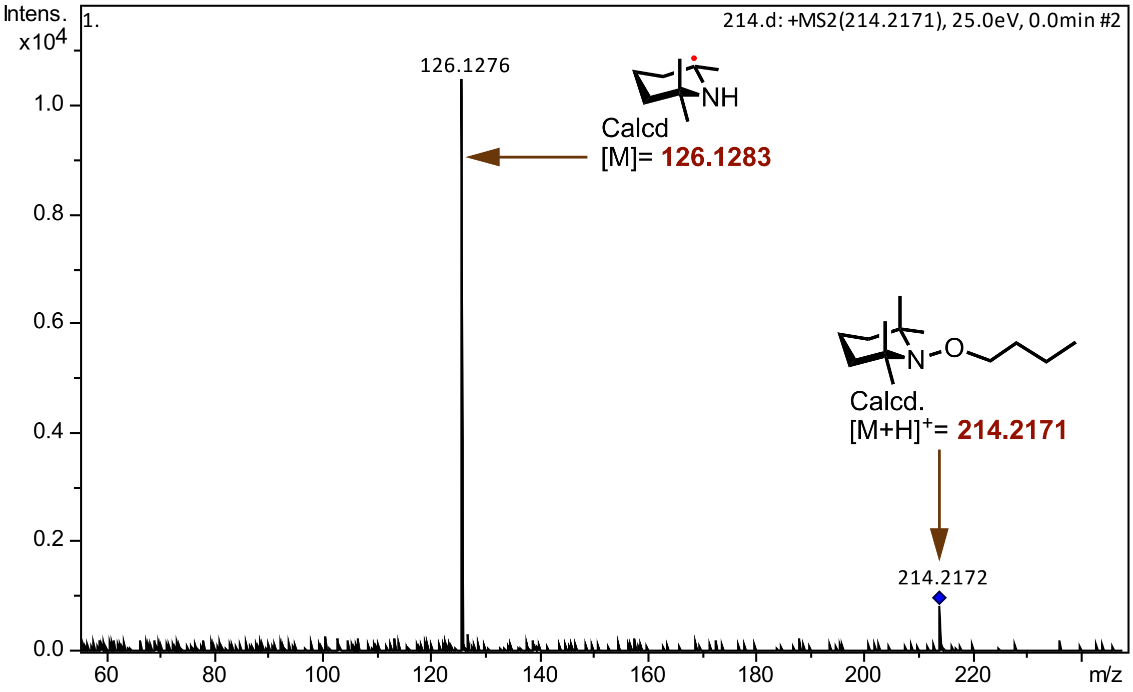

The MS-MS analysis of TMP-O-^{n}Bu showed its fragmentation in the following ion:

1) (TMPH – Me) ion [M-H]= 126.1276

This study confirms the presence of TMP-O-^{n}Bu species.

C. HRMS (MS-MS) of TMP_2O

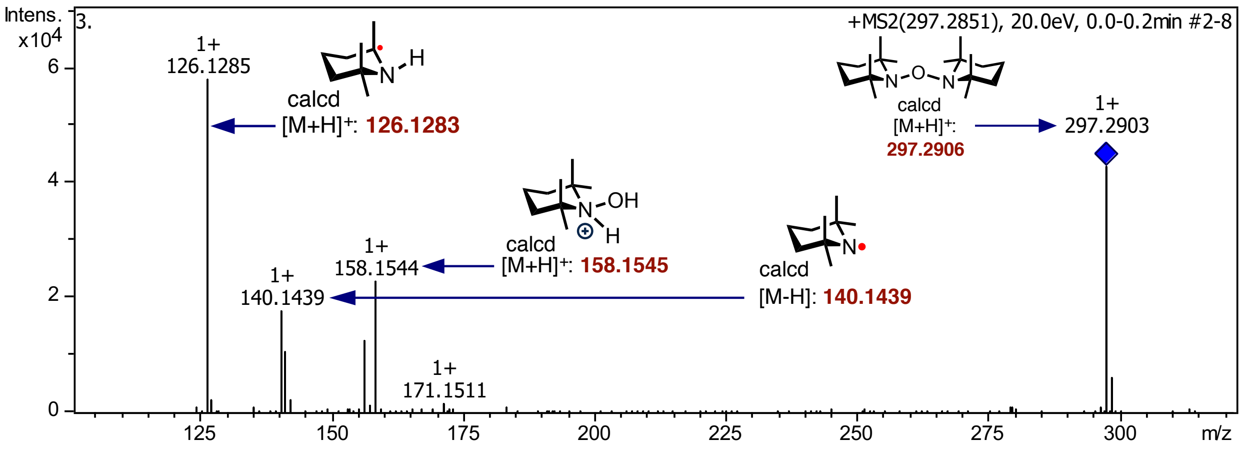

The MS-MS analysis of TMP_2O showed its fragmentation in tree main ions:

1) (TEMPO-H + H) ion $[M+H]^+$=158.1544

2) (TMP·) ion [M-H]= 140.1439
3) (TMP-H – Me) ion [M-H]= 126.1283

This study confirms the identification and identity of the TMP_2O species.

2. Mechanistic Studies of the double arylation using the system TMP_2O/ Ph_2IOTf

2-phenoxynaphthalene [4]

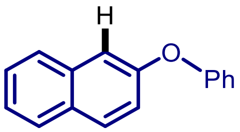

To a solution of NaOH (2.0 equiv., 1.0 mmol) in distilled water (2.5 mL), 2-napthol (1.0 equiv., 0.5 mmol) was added at 23 ºC and the mixture was stirred for 10 minutes. Diphenyliodonium salt (1.2 equiv., 0.6 mmol) was added in one portion and the reaction was vigorously stirred up at 23 ºC for 12 h. The reaction was extracted with EtOAc, dried over Na_2SO_4, filtered and concentrated in vacuum. The crude material was purified with flash chromatography over silica gel with the system (3% EtOAc/Hexane) to afford the product 2-phenoxynaphthalene (86 mg, 78%) as a white solid. R_f= 0.5 (5% EtOAc/Hexane). The spectroscopic data match perfectly with those described previously in literature.[4]

^{1}H NMR (500 MHz, $CDCl_3$) δ 7.84 (d, J = 14.5 Hz, 2H), 7.71 (d, J = 8.1 Hz, 1H), 7.48 – 7.36 (m, 4H), 7.33 (d, J = 2.3 Hz, 1H), 7.28 (dd, J = 8.9, 2.4 Hz, 1H), 7.15 (t, J = 7.4 Hz, 1H), 7.09 (dd, J = 8.6, 0.9 Hz, 2H).

^{13}C NMR (126 MHz, $CDCl_3$) δ 157.3, 155.2, 134.7, 130.9, 130.1, 129.9, 127.8, 127.6, 126.6, 124.8, 123.8, 120.3, 119.7, 114.2.

Equation 5

Attempt to get 2-phenoxynaphthalene

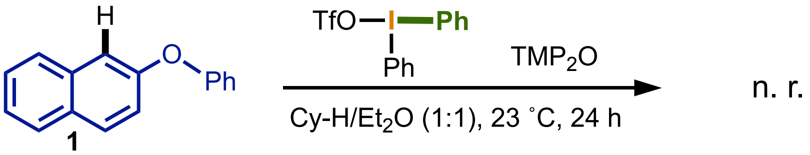

A 25 mL round bottom flask with a stir bar was fitted with a rubber septum and flame dried under high vacuum. The flask was purged with argon and charged with 2-phenoxynaphthalene (22 mg, 0.1 mmol, 1 equiv), anhydrous diethyl ether and cyclohexane (1:1) at 25 °C. Then 627 mg of the solids mixture which contains TMP_2O (50.4 mg, 0.17 mmol, 1.7 equiv) was added and stirred for 15 min obtaining a homogeneous mixture. Then diphenyliodonium nitrate (107.5 mg, 0.25 mmol, 2.5 equiv) was added and stirred at 25 °C during 24 h. Only starting material was recovered.

1-phenylnaphthalen-2-ol [5]

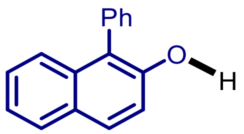

The following substrate was prepared by Suzuki-Miyaura cross-coupling reaction. A 50 mL round bottom flask with a stir bar was fitted with a rubber septum and flame dried under high vacuum. The flask was purged with argon and successively charged with $Pd(PPh_3)_4$ (106.2 mg, 0.1 mmol), K_2CO_3 (445.2 mg, 4.2 mmol), 1-bromonaphthalen-2-ol (410 mg, 2.0 mmol), phenylboronic acid (4.0 mmol), 10.0 mL 1,4-dioxene, and 2 mL distilled water. The reaction mixture was then heated at 80 °C for 12 h. After the reaction was cooled down to room temperature, the organic layer was separated, and the aqueous layer was extracted with ethyl acetate (3 × 10 mL) and the combined organic layer was dried over Na_2SO_4 and concentrated. The crude products were purified by flash chromatography on silica gel (6% EtOAc/Hexane) to afford the product 1-phenylnaphthalen-2-ol (86 mg, 78%) as a white solid. R_f= 0.15 (10% EtOAc/Hexane). The spectroscopic data match perfectly with those described previously in literature.[5]

^{1}H NMR (400 MHz, $CDCl_3$) δ 7.46 (d, J = 7.3 Hz, 3H), 7.38 (t, J = 7.5 Hz, 3H), 7.31 (d, J = 7.3 Hz, 1H), 7.25 (dd, J = 8.0, 1.4 Hz, 1H), 7.16 (dd, J = 7.9, 6.9 Hz, 2H), 6.86 (t, J = 7.8 Hz, 1H), 5.64 (s, 1H).

Equation 6

2-phenoxy-2-phenylnaphthalene

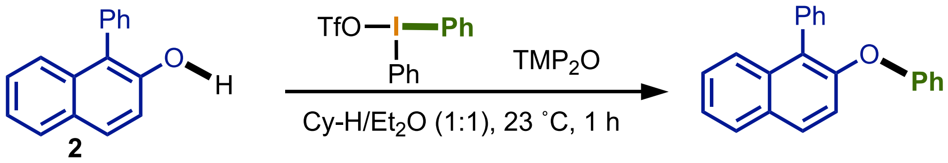

A 25 mL round bottom flask with a stir bar was fitted with a rubber septum and flame dried under high vacuum. The flask was purged with argon and charged with 1-phenyl-2-naphthol (22 mg, 0.1 mmol, 1 equiv), anhydrous diethyl ether and cyclohexane (1:1) at 25 °C. Then 627 mg of the solids mixture which contains TMP_2O (50.4 mg, 0.17 mmol, 1.7 equiv) was added and stirred for 15 min obtaining a homogeneous mixture. Then diphenyliodonium nitrate (107.5 mg, 0.25 mmol, 2.5 equiv) was added and stirred at 25 °C until fully consumption of the starting material. The organic layer was separated, and the aqueous layer was extracted with ethyl acetate (3 × 10 mL), the combined organic layers were dried over Na_2SO_4 and concentrated. The crude product was purified by flash chromatography on silica gel (10% EtOAc/Hexane) to afford the product 2-phenoxy-1-phenylnaphthalene (88 mg, 86%) as gel solid. R_f= 0.5 (4% EtOAc/Hexane).

^{1}H NMR (500 MHz, $CDCl_3$) δ 7.87 (dd, *J* = 11.8, 8.3 Hz, 2H), 7.63 (d, *J* = 8.4 Hz, 1H), 7.49 – 7.33 (m, 7H), 7.25 – 7.21 (m, 3H), 6.99 (t, *J* = 7.4 Hz, 1H), 6.87 (dd, *J* = 8.6, 0.9 Hz, 2H).

^{13}C NMR (126 MHz, $CDCl_3$) δ 158.6, 150.4, 135.5, 133.9, 131.1, 130.7, 130.3, 129.6, 129.4, 128.3, 128.4, 127.7, 126.6, 126.6, 125.5, 122.9, 120.7, 117.8.

Equation 7

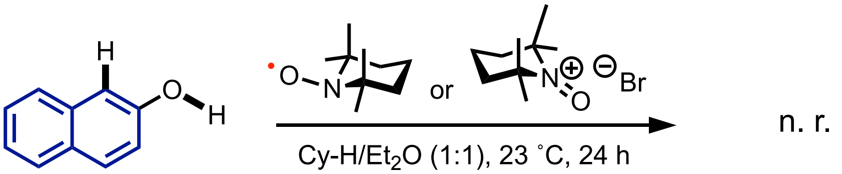

A 25 mL round bottom flask with a stir bar was fitted with a rubber septum and flame dried under high vacuum. The flask was purged with argon and charged with 2-naphthol (14.4 mg, 0.1 mmol, 1 equiv), TEMPO (26.6 mg, 0.17 mmol, 1.7 equiv) or 2,2,6,6-tetramethyl-1-oxopiperidin-1-ium bromide (39.9 mg, 0.17 mmol, 1.7 equiv), anhydrous diethyl ether and cyclohexane (1:1) at 25 °C. the reaction was stirred at 25 °C during 24 h. No reaction observed.

Equation 8

Trapping experiment of TMP_2O

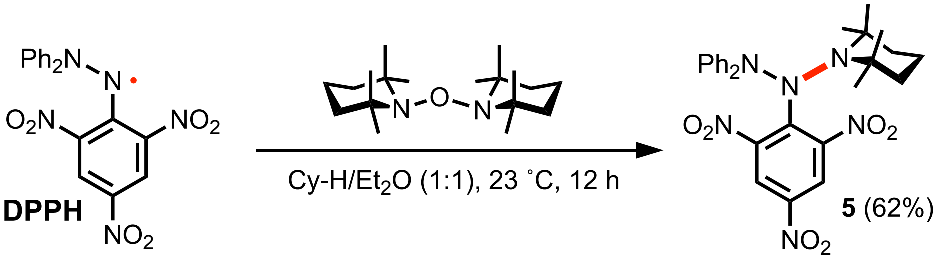

A 25 mL round bottom flask with a stir bar was fitted with a rubber septum and flame dried under high vacuum. The flask was charged with 890 mg of the solids mixture which contains TMP_2O (71.2 mg, 0.24 mmol, 2.4 equiv) and 2,2-diphenyl-1-picrylhydrazyl (DPPH) (39.4 mg, 0.1 mmol, 1 equiv), anhydrous diethyl ether and cyclohexane (1:1). The mixture was stirred at 25 °C until fully consumption of the starting material. The organic layer was separated and the aqueous layer was extracted with ethyl acetate (3 × 10 mL), and the combined organic layers were dried over Na_2SO_4 and concentrated. The crude products were purified by flash chromatography on silica gel (10% EtOAc/Hexane) to afford the product (73 mg, 64%) as brown solid. m.p.= 100 °C dec. R_f= 0.15 (4% EtOAc/Hexane) a single spot is observed.

IR (neat) ν/cm^{-1} = 3580, 3429, 3364, 3121, 3052, 2920, 1604.

^1H NMR (500 MHz, $CDCl_3$) δ 9.15 (s, 1H), 8.45 (s, 1H), 7.31 – 7.26 (m, 4H), 7.14 (t, J = 7.4 Hz, 2H), 7.04 (dt, J = 8.9, 1.8 Hz, 4H), 1.23 – 1.13 (m, 15H), 0.84 – 0.78 (m, 3H).

^{13}C NMR (126 MHz, $CDCl_3$) δ 146.0, 142.4, 136.9, 129.6, 126.6, 125.9, 125.1, 120.5, 59.5, 38.5, 31.9, 31.4, 29.7, 29.7, 22.7, 14.2.

HRMS (EI): m/z calculated for $C_{27}H_{29}N_6O_6$ $[M\text{-}H]^+$= 533.2154, found 533.5289

Equation 9[6]

First trapping experiment of a phenyl group from Ph_2INO_3

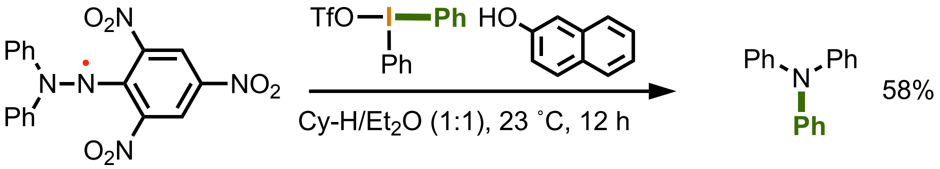

A 25 mL round bottom flask with a stir bar was fitted with a rubber septum and flame dried under high vacuum. The flask was successively charged with 2-naphthol (14.4 mg, 0.1 mmol, 1 equiv), 2,2-diphenyl-1-picrylhydrazyl (DPPH) (39.4 mg, 0.1 mmol, 1 equiv), diphenyliodonium nitrate (107.5 mg, 0.25 mmol, 2.5 equiv) and anhydrous diethyl ether-cyclohexane (1:1) at 25 °C. diaryliodonium salt (2.4 equiv), was added and stirred for 15 min obtaining a homogeneous mixture. The reaction was stirred at 25 °C until fully consumption of the starting material. The organic layer was separated, and the aqueous layer was extracted with ethyl acetate (3 × 10 mL). The combined organic layers were dried over Na_2SO_4 and concentrated. The crude material was purified by flash column chromatography over silica gel with the system (3% EtOAc/Hexane) to afford the product triphenylamine (62 mg, 58%) as a white solid. R_f= 0.5 (5% EtOAc/Hexane).

^{1}H NMR (500 MHz, $CDCl_3$) δ 7.38 – 7.33 (m, 6H), 7.21 (t, J = 7.4 Hz, 3H), 7.13 – 7.09 (m, 6H).

^{13}C NMR (126 MHz, $CDCl_3$) δ 146.5, 129.7, 126.7, 120.6.

Equation 10[6]

Second trapping experiment of a phenyl group from Ph_2INO_3

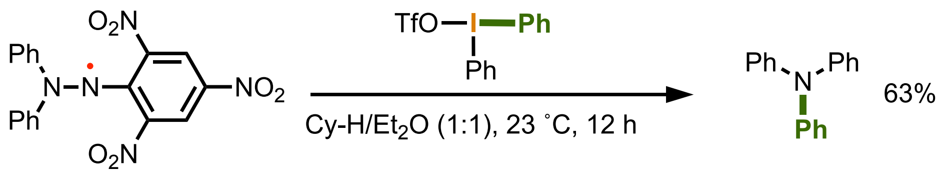

A 25 mL round bottom flask with a stir bar was fitted with a rubber septum and flame dried under high vacuum. The flask was successively charged with 2,2-diphenyl-1-picrylhydrazyl (DPPH) (39.4 mg, 0.1 mmol, 1 equiv), diphenyliodonium nitrate (107.5 mg, 0.25 mmol, 2.5 equiv) and anhydrous diethyl ether-cyclohexane (1:1) at 25 °C. The reaction was stirred at 25 °C until fully consumption of the starting material. The organic layers were separated, and the aqueous layer was extracted with ethyl acetate (3 × 10 mL). The combined organic layers were dried over Na_2SO_4 and concentrated. The crude material was purified by flash column chromatography over silica gel with the system (3% EtOAc/Hexane) to afford the product triphenylamine (62 mg, 58%) as a white solid. R_f= 0.5 (5% EtOAc/Hexane).

^{1}H NMR (500 MHz, $CDCl_3$) δ 7.38 – 7.33 (m, 6H), 7.21 (t, J = 7.4 Hz, 3H), 7.13 – 7.09 (m, 6H).

^{13}C NMR (126 MHz, $CDCl_3$) δ 146.5, 129.7, 126.7, 120.6.

Equation 11.

dibenzo[*b*,*d*]furan (8)[2]

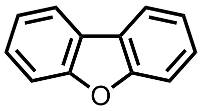

This compound was obtained by cyclization of 2-phenylphenol **6** using 2.5 equivalents of TMP_2O. It was obtained dibenzo[*b*,*d*]furan in **8** in 8% of yield. The physicochemical and spectroscopic data perfectly match with the previous ones reported for this compound.

^{1}H NMR (500 MHz, $CDCl_3$) δ 7.97 (d, *J*=8.4 Hz, 2H), 7.59 (d, *J*=8.4 Hz, 2H), 7.48 (t, *J*=8.4 Hz, 2H), 7.36 (t, *J*=8.4 Hz, 2H).

^{13}C NMR (126 MHz, $CDCl_3$) δ 156.2, 127.1, 124.0, 122.7, 120.7, 111.7.

Equation 12.

9-tosy-9*H*-carbazole (9)[3]

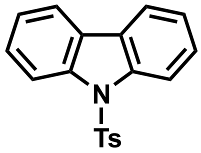

This compound was obtained by cyclization of 2-phenyl-*N*-tosylaniline **7** using 3 equivalents of TMP_2O. It was obtained 9-tosyl-9*H*-lcarbazole in **9** in 14% of yield. The physicochemical and spectroscopic data perfectly match with the previous ones reported for this compound.

^{1}H NMR (500 MHz, $CDCl_3$) δ 8.33 (d, *J* = 8.2 Hz, 2H), 7.90 (d, *J* = 8.2 Hz, 2H), 7.70 (d, *J* = 8.2 Hz, 2H), 7.50 (t, *J* = 8.2 Hz, 2H), 7.47 (t, *J* = 8.2 Hz, 2H), 7.10 d, *J* = 8.2 Hz, 2H), 2.26 (s, 3H).

^{13}C NMR (126 MHz, $CDCl_3$) δ 145.0, 138.5, 135.2, 129.8, 127.5, 126.6, 126.5, 124.0, 120.1, 15.3, 21.6.

3. Computational Methodology

Gas-phase geometry optimizations were performed using the Gaussian 09 suite of programs.[7] Theoretical calculations were done choosing the hybrid, range-separated density-functional ω-B97XD,[6] which considers dispersion interaction through a range separation (22% when in short range and 100% HF for long range). It has been established to give better results for charge transfer excitations as for our study case. On the other hand, we have set up the triple-ζ quality basis set, 6-311G(d), with one polarization function for all the atoms but hydrogens. The LANL08d pseudopotential basis set was used for I atom.[7] Thus, after running our geometry optimizations, we have performed harmonic frequency calculations at the same level for a two-fold aim: 1) to be sure all the reported structures are minima on the potential energy surface (each reported geometry gave positive frequency values only) and 2) to get the zero-point energy correction as well as the thermodynamic factors to convert the electronic energy into the scale of enthalpy and free energy at 298K and 1 atm. This way, the reported energies herein are expressed in terms of H and G, as pointed out in the text of our manuscript.

Also, solvent-phase corrections were considered through single-point calculations using the optimized geometries at the same level of theory and considering the SMD variation of IEFPCM of Truhlar and coworkers.[8] We selected the solvent parameters for diethyl ether ($\varepsilon = 4.24$) to give final energy values.

Calculations of Schemes 4 and 5.

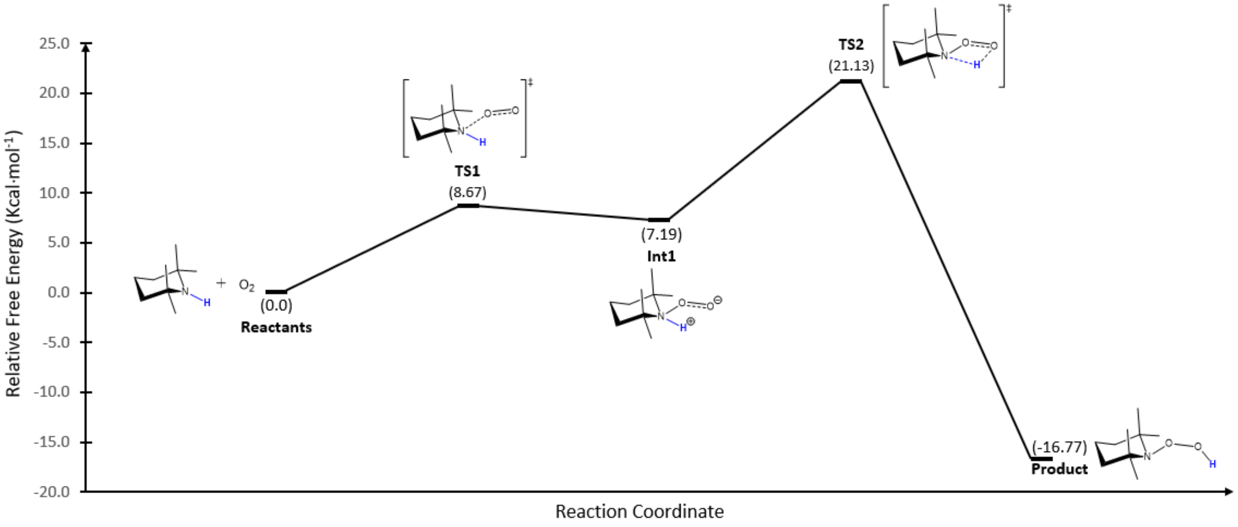

Figure S1. Free-energy profile for the reaction between 2,2,6,6-tetramethylpiperidine (TMP) and molecular oxygen calculated at the (SMD:diethylether)ω-B97XD/6-311G(d) level.

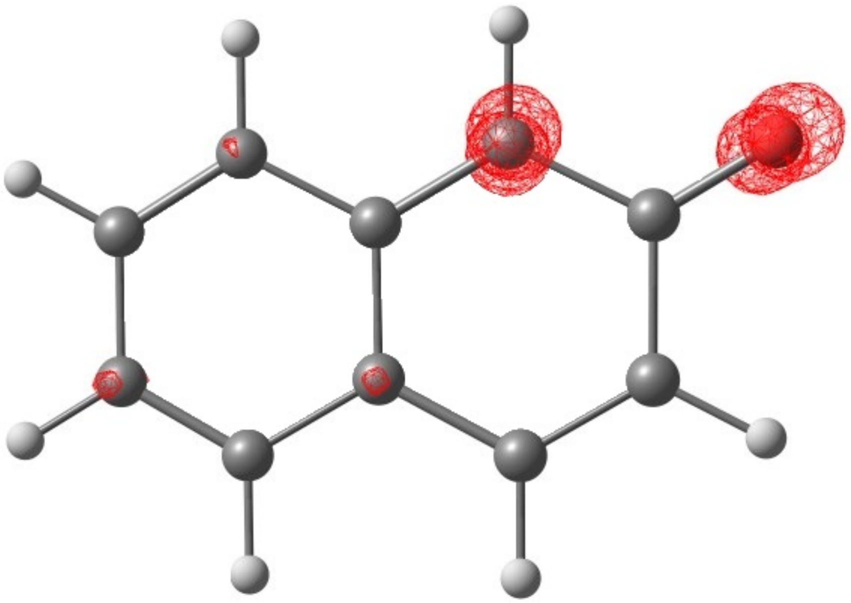

Figure S2. Isosurface of spin electron density (0.027 a. u.) where it is shown the highest probability of locating the unpaired electron of the naphthol radical calculated at the ω-UB97XD/6-31g(d) level.

Calculations for exploring the possible ionic pathway of arylation

We also considered the possibility that the radical precursors did, indeed, not form radicals as we suggest in the manuscript, but they react with 2-naphthol. Therefore, some deprotonation reactions were calculated (Scheme S1). Either TMP-O-*n*Bu or TMP_2O produced very exergonic (and exothermic) free energies which means those reaction pathways are very unfavourable. Moreover, the deprotonation via the attack of one nitrogen of TMP-O-*n*Bu or TMP_2O gave the half of energy values than for the attack of central oxygen. These cations did not split into two fragments from optimization calculations, so they can be considered as intermediates before that can happen and, because of the high energies involved, it is very unlikely that deprotonation reactions among these species can occur. Thus, an ionic reaction between 2-naphthol and TMP-O-*n*Bu or TMP_2O does not follow.

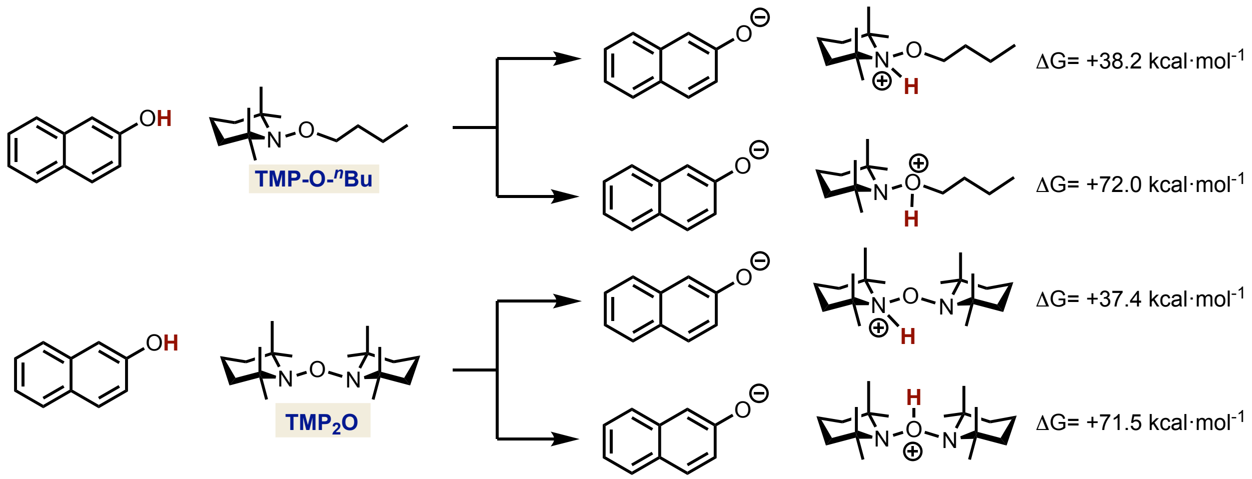

Scheme S1. Calculated reaction energies for testing ionic reaction pathways calculated at the (SMD:diethylether)ω-B97XD/6-311G(d) level.

Table S1. Cartesian coordinates (x-y-z format) of all the optimized geometries calculated at the ω-B97XD/6-311G(d) level.

2–naphthol

E(scf) = –460.950514634 a.u.

C	–2.811384	0.877349	0.000000
C	–2.219361	–0.358364	0.000000
C	–0.806004	–0.486610	0.000000
C	0.000000	0.686601	0.000000
C	–0.644069	1.953366	0.000000
C	–2.011319	2.045609	0.000000
H	–0.769440	–2.651150	0.000000
H	–3.893492	0.966659	0.000000
H	–2.825917	–1.260886	0.000000
C	–0.161765	–1.749832	0.000000
C	1.411004	0.564715	0.000000
H	–0.031639	2.851391	0.000000
H	–2.489083	3.020938	0.000000
C	2.001267	–0.674810	0.000000
C	1.204092	–1.847761	0.000000
H	2.036619	1.451995	0.000000
H	1.683925	–2.824315	0.000000
O	3.360188	–0.743487	0.000000
H	3.632761	–1.668306	0.000000

O_2

E(scf) = –150.209389773 a.u.

O	0.000000	0.000000	0.602839
O	0.000000	0.000000	–0.602839

OH anion

E(scf) = –75.6979240905 a.u.

O	0.000000	0.000000	0.108785
H	0.000000	0.000000	–0.870280

OH radical

E(scf) = –75.6985340101 a.u.

O	0.000000	0.000000	0.108562

$O(TMP)_2$

E(scf) = –891.988943793 a.u.

O	0.082383	–0.059352	–0.486916
C	–1.803403	1.140830	0.554391
C	–1.959455	–1.077105	–0.695787
C	–3.134783	0.781617	1.251786
C	–3.294963	–1.364540	0.012028
C	–4.048486	–0.105072	0.416279
H	–3.638462	1.719580	1.514480
H	–2.899367	0.266088	2.191628
H	–3.906269	–1.992102	–0.647617
H	–3.080393	–1.954184	0.912958
H	–4.942348	–0.370760	0.992146
H	–4.405290	0.430759	–0.471537
N	–1.178214	–0.167677	0.194993
C	–2.064075	2.138729	–0.596010
H	–2.076383	3.158394	–0.196059
H	–1.283190	2.087895	–1.352186
H	–3.024960	1.979342	–1.090294
C	–0.947133	1.853809	1.615190
H	–1.548242	2.654630	2.062810
H	–0.647516	1.160520	2.404171
H	–0.042277	2.288520	1.193490
C	–2.191189	–0.543443	–2.127316
H	–2.543033	–1.361464	–2.765340
H	–2.937233	0.249874	–2.178404
H	–1.256159	–0.167160	–2.551079
C	–1.224157	–2.418269	–0.846749
H	–0.978670	–2.863358	0.119985
H	–1.877957	–3.119050	–1.377236
H	–0.306038	–2.306742	–1.425798
C	2.096551	0.990011	–0.706331
C	1.786134	–0.782630	1.086074
C	2.689406	–0.088551	–1.637842
C	2.386641	–1.839200	0.140696
C	3.351298	–1.241073	–0.883811
H	3.405366	0.385617	–2.320833
H	1.876082	–0.489154	–2.255423

H	0.000000	0.000000	−0.868493

n-butane

E(scf) = –158.407816264 a.u.

C	−0.704211	1.825374	0.000000
H	−0.189432	2.218082	0.884635
H	−0.189432	2.218082	−0.884635
C	−0.704211	0.297779	0.000000
H	−1.250967	−0.070957	0.878316
H	−1.250967	−0.070957	−0.878316
C	0.704211	−0.297779	0.000000
H	1.250967	0.070957	0.878316
H	1.250967	0.070957	−0.878316
C	0.704211	−1.825374	0.000000
H	1.722630	−2.227459	0.000000
H	0.189432	−2.218082	0.884635
H	0.189432	−2.218082	−0.884635
H	−1.722630	2.227459	0.000000

2–naphthol_radical

E(scf) = –460.313414425 a.u.

C	–2.805618	0.676243	0.000000
C	–2.117670	–0.524438	0.000000
C	–0.715487	–0.548579	0.000000
C	0.000000	0.688596	0.000000
C	–0.731900	1.905990	0.000000
C	–2.107397	1.898642	0.000000
H	–0.542852	–2.717117	0.000000
H	–3.891352	0.677089	0.000000
H	–2.661996	–1.465103	0.000000
C	0.023770	–1.788947	0.000000
C	1.405072	0.666766	0.000000
H	–0.184250	2.844407	0.000000
H	–2.657704	2.834183	0.000000
C	2.152018	–0.569131	0.000000
C	1.374105	–1.811731	0.000000
H	1.976545	1.590687	0.000000
H	1.934914	–2.740711	0.000000
O	3.395667	–0.572988	0.000000

H	2.895700	–2.602894	0.741969
H	1.569145	–2.344746	–0.383652
H	3.666162	–2.014402	–1.593555
H	4.267236	–0.894788	–0.388838
N	1.190205	0.378929	0.332904
C	0.731042	–1.430719	1.994445
H	0.413188	–0.730747	2.773112
H	–0.157750	–1.746616	1.462782
H	1.187708	–2.298845	2.483662
C	2.854482	–0.259634	2.070972
H	2.522413	0.675215	2.531325
H	2.981759	–1.000520	2.866541
H	3.837760	–0.103859	1.629672
C	1.300738	1.985033	–1.563101
H	0.533060	1.492018	–2.157978
H	0.830460	2.749192	–0.936876
H	1.989988	2.487922	–2.249593
C	3.202965	1.826163	–0.034615
H	3.597350	2.538116	–0.766133
H	2.794830	2.395099	0.805535
H	4.050643	1.242922	0.320417

TMP

E(scf) = –409.051745139 a.u.

H	–0.000037	–1.119137	–1.355704
C	–1.274821	–0.260557	–0.042415
C	1.274833	–0.260579	–0.042397
C	–1.254431	1.230954	–0.441626
C	1.254473	1.230923	–0.441664
C	0.000036	1.952849	0.051852
H	–2.159227	1.727296	–0.068446
H	–1.285507	1.298326	–1.539157
H	2.159302	1.727240	–0.068549
H	1.285485	1.298251	–1.539204
H	0.000037	2.987961	–0.308416
H	0.000044	2.011944	1.147604
N	–0.000001	–0.937302	–0.354748
C	2.353166	–0.989712	–0.851778
H	2.387337	–2.049020	–0.576935
H	2.152833	–0.919594	–1.928604
H	3.338398	–0.547955	–0.669465
C	1.623515	–0.419534	1.446366

H	1.471101	–1.458724	1.751735
H	2.672588	–0.152528	1.616141
H	1.018632	0.216934	2.095909
C	–2.353220	–0.989653	–0.851760
H	–2.152840	–0.919679	–1.928578
H	–2.387627	–2.048932	–0.576845
H	–3.338401	–0.547706	–0.669540
C	–1.623512	–0.419552	1.446348
H	–2.672663	–0.152829	1.616098
H	–1.470836	–1.458695	1.751734
H	–1.018839	0.217127	2.095900

O(TMP)_radical

E(scf) = –483.588178170 a.u.

O	0.000078	–2.010987	–0.086839
C	–1.316613	–0.071910	–0.026209
C	1.316602	–0.071859	–0.026214
C	–1.241992	1.388887	–0.496796
C	1.241962	1.388943	–0.496754
C	–0.000042	2.122772	–0.002636
H	–2.156779	1.897328	–0.170997
H	–1.243681	1.405663	–1.594901
H	2.156715	1.897387	–0.170866
H	1.243728	1.405805	–1.594860
H	–0.000068	3.150763	–0.380948
H	–0.000063	2.195476	1.091726
N	0.000008	–0.741056	–0.221847
C	–1.718151	–0.167126	1.455347
H	–2.762462	0.138458	1.579657
H	–1.612631	–1.201358	1.794018
H	–1.102582	0.471590	2.094885
C	–2.340968	–0.829888	–0.875979
H	–3.310974	–0.325545	–0.811474
H	–2.028790	–0.852124	–1.924817
H	–2.450353	–1.858223	–0.528113
C	1.718171	–0.167131	1.455333
H	2.762535	0.138339	1.579574
H	1.102730	0.471656	2.094904
H	1.612583	–1.201358	1.793999
C	2.340934	–0.829831	–0.876022
H	2.028723	–0.852007	–1.924847
H	3.310949	–0.325509	–0.811498

2–naphthol_anion

E(scf) = –460.379324702 a.u.

C	–2.855269	0.603421	0.000000
C	–2.127724	–0.567654	0.000000
C	–0.718346	–0.567718	0.000000
C	0.000000	0.677170	0.000000
C	–0.788411	1.871698	0.000000
C	–2.160690	1.836185	0.000000
H	–0.476058	–2.721893	0.000000
H	–3.942679	0.584094	0.000000
H	–2.642448	–1.529293	0.000000
C	0.053738	–1.767286	0.000000
C	1.400830	0.690114	0.000000
H	–0.268243	2.828646	0.000000
H	–2.723039	2.769191	0.000000
C	2.198401	–0.495712	0.000000
C	1.415361	–1.738401	0.000000
H	1.925461	1.644907	0.000000
H	1.989931	–2.663892	0.000000
O	3.453717	–0.520334	0.000000

H	2.450311	–1.858189	–0.528218

HO(TMP)$_2$ cation 1

E(scf) = –892.333747082 a.u.

O	0.047952	–0.017738	–0.420884
C	–1.888147	1.158479	0.568169
C	–2.004824	–1.111867	–0.695994
C	–3.246669	0.793080	1.211005
C	–3.361832	–1.369888	–0.009674
C	–4.123425	–0.099438	0.342496
H	–3.752688	1.735825	1.443817
H	–3.048688	0.289895	2.164639
H	–3.948730	–2.011017	–0.675482
H	–3.174947	–1.942458	0.906806
H	–5.035766	–0.357562	0.888175
H	–4.450244	0.424670	–0.562691
N	–1.280855	–0.166365	0.224749
C	–2.104808	2.147888	–0.596714
H	–2.216951	3.152058	–0.179017
H	–1.267354	2.203552	–1.299186
H	–3.002668	1.938321	–1.177795
C	–1.041021	1.843154	1.648943
H	–1.621254	2.670054	2.068972
H	–0.803545	1.147642	2.455686
H	–0.106007	2.246037	1.257769
C	–2.189504	–0.608247	–2.139979
H	–2.592240	–1.427016	–2.742679
H	–2.880158	0.228599	–2.231245
H	–1.237008	–0.328173	–2.606841
C	–1.244792	–2.441753	–0.758178
H	–1.033582	–2.839463	0.236627
H	–1.868011	–3.173094	–1.280638
H	–0.309004	–2.353132	–1.315259
C	2.199008	0.949757	–0.765971
C	1.839460	–0.737527	1.142586
C	2.692544	–0.204029	–1.654351
C	2.367820	–1.846622	0.218961
C	3.326458	–1.342438	–0.857820
H	3.402513	0.203127	–2.382443
H	1.847277	–0.600057	–2.233288
H	2.861833	–2.596544	0.845626
H	1.518371	–2.351286	–0.252869

O(TMP)_cation

E(scf) = –483.338119585 a.u.

O	0.000084	–1.941458	–0.319045
C	–1.349553	–0.073244	0.010921
C	1.349486	–0.073223	0.010888
C	–1.246921	1.329778	–0.614441
C	1.246853	1.329873	–0.614333
C	–0.000075	2.106026	–0.208221
H	–2.157636	1.863380	–0.325438
H	–1.275906	1.226639	–1.705911
H	2.157497	1.863472	–0.325107
H	1.276013	1.226899	–1.705816
H	–0.000069	3.073813	–0.716548
H	–0.000172	2.325766	0.864746
N	0.000027	–0.769043	–0.125532
C	–1.655846	–0.026710	1.524024
H	–2.704364	0.267180	1.620474
H	–1.538145	–1.010915	1.985481
H	–1.052395	0.702622	2.063711
C	–2.386931	–0.933762	–0.706370
H	–3.329961	–0.381085	–0.695594
H	–2.112595	–1.112693	–1.749847
H	–2.548168	–1.892723	–0.210874
C	1.656016	–0.026661	1.523968
H	2.704793	0.266387	1.620148
H	1.053307	0.703369	2.063533
H	1.537677	–1.010655	1.985707
C	2.386808	–0.933750	–0.706550
H	2.112276	–1.112809	–1.749951
H	3.329796	–0.381001	–0.695991
H	2.548169	–1.892645	–0.210961

OBu radical

E(scf) = –232.923544701 a.u.

O	2.534221	–0.345828	0.005928
C	1.394824	0.404280	–0.012213
H	1.439144	1.117543	–0.858426
H	1.458253	1.057762	0.883722
C	0.086256	–0.383294	–0.007576

H	3.581589	–2.161666	–1.536132
H	4.272125	–1.015446	–0.412042
N	1.330631	0.465856	0.386079
C	0.760274	–1.286383	2.084688
H	0.482794	–0.542311	2.835235
H	–0.140998	–1.609140	1.576049
H	1.189164	–2.146868	2.606046
C	2.949722	–0.211196	2.085406
H	2.693040	0.767104	2.499255
H	3.027383	–0.913849	2.918501
H	3.933735	–0.159736	1.624477
C	1.419418	1.976787	–1.615097
H	0.677715	1.544917	–2.305787
H	0.951593	2.745338	–0.991954
H	2.123630	2.477607	–2.282662
C	3.361212	1.753681	–0.148096
H	3.809361	2.359271	–0.939565
H	3.002481	2.428503	0.633229
H	4.153792	1.132368	0.257911
H	–0.035971	0.69876	–1.082245

H	0.064162	–1.029918	–0.894034
H	0.080040	–1.046573	0.866250
C	–1.144647	0.523376	0.007424
H	–1.115406	1.194467	–0.861894
H	–1.110622	1.170663	0.894641
C	–2.455449	–0.261023	0.000295
H	–3.320399	0.409810	0.013736
H	–2.530732	–0.889739	–0.894003
H	–2.524121	–0.917430	0.875001

TMP_radical

E(scf) = –408.393125533 a.u.

C	–1.262864	–0.268165	–0.061969
C	1.262864	–0.268166	–0.061969
C	–1.240144	1.230272	–0.433759
C	1.240145	1.230272	–0.433759
C	0.000000	1.946407	0.090171
H	–2.158117	1.702630	–0.063772
H	–1.258817	1.320753	–1.528616
H	2.158117	1.702629	–0.063770
H	1.258819	1.320754	–1.528615
H	0.000000	2.991537	–0.239072
H	0.000000	1.969220	1.187270
N	0.000000	–0.968088	–0.252673
C	2.288809	–0.998282	–0.941252
H	2.359656	–2.050862	–0.651140
H	1.989474	–0.955344	–1.993526
H	3.278263	–0.536147	–0.844029
C	1.679821	–0.449998	1.416444
H	1.561343	–1.495619	1.715260
H	2.732139	–0.169652	1.536204

Product1

E(scf) = –943.939641811 a.u.

C	3.297882	–2.454368	–0.638827
C	3.602303	–1.414981	0.233673
C	2.849497	–0.239081	0.224103
C	1.765051	–0.118177	–0.658718
C	1.480239	–1.152562	–1.542119
C	2.243238	–2.318326	–1.536883
H	4.005030	0.717874	1.814198
H	3.889500	–3.364672	–0.625246
H	4.436554	–1.509912	0.924172
C	3.201103	0.882098	1.099346
C	0.893338	1.109300	–0.608847
H	0.639133	–1.052899	–2.220416
H	2.009418	–3.121019	–2.229625
C	1.565944	2.340364	0.015658
C	2.617334	2.088200	1.014316
H	0.574487	1.380491	–1.617053
H	2.931568	2.928987	1.623897
O	1.183155	3.456213	–0.272599
O	–0.246982	0.984197	0.255563

H	1.085540	0.169992	2.092579
C	–2.288809	–0.998282	–0.941252
H	–1.989475	–0.955345	–1.993526
H	–2.359656	–2.050862	–0.651139
H	–3.278263	–0.536146	–0.844029
C	–1.679821	–0.449997	1.416444
H	–2.732139	–0.169651	1.536204
H	–1.561343	–1.495619	1.715261
H	–1.085539	0.169993	2.092579

n-butane radical

E(scf) = –157.732318655 a.u.

C	1.998992	–0.092170	0.001108
H	2.432784	–0.492502	0.912622
H	2.497964	–0.323511	–0.934839
C	0.638612	0.514434	0.012883
H	0.509798	1.134238	0.911354
H	0.515365	1.182514	–0.850003
C	–0.497745	–0.531612	–0.014680
H	–0.388044	–1.201348	0.847288
H	–0.387438	–1.156921	–0.909038
C	–1.883177	0.112058	0.001529
H	–2.673386	–0.645542	–0.013550
H	–2.022111	0.724075	0.900177
H	–2.025027	0.762742	–0.869049

Product2

E(scf) = –943.969342032 a.u.

C	–4.726136	–1.541477	0.000112
C	–4.652724	–0.172424	–0.000036
C	–3.401659	0.493839	–0.000011
C	–2.209220	–0.280430	0.000169
C	–2.311279	–1.697474	0.000319
C	–3.539413	–2.308703	0.000291
H	–4.209169	2.505324	–0.000293
H	–5.691453	–2.038572	0.000058
H	–5.559376	0.427776	–0.000186
C	–3.301112	1.908982	–0.000151
C	–0.947622	0.381617	0.000179

C	–1.368115	–0.869652	1.135078
C	–2.331281	0.544631	–0.764866
C	–2.379293	–1.973608	0.780612
C	–3.313655	–0.601170	–1.067563
C	–3.662742	–1.439844	0.155892
H	–2.594645	–2.544049	1.691911
H	–1.900055	–2.663141	0.073175
H	–4.215091	–0.166915	–1.515828
H	–2.858316	–1.253117	–1.825125
H	–4.314954	–2.271430	–0.133664
H	–4.227920	–0.843526	0.881947
N	–1.140505	–0.066904	–0.103781
C	–1.836859	–0.051263	2.356307
H	–1.664195	–0.632013	3.268603
H	–1.264340	0.876282	2.432145
H	–2.898869	0.197834	2.329525
C	–0.049653	–1.548380	1.524993
H	–0.236583	–2.248782	2.346374
H	0.374669	–2.105163	0.686480
H	0.684296	–0.814470	1.865911
C	–3.011176	1.679460	0.025239
H	–3.652352	2.256495	–0.649832
H	–3.640070	1.323047	0.842276
H	–2.254786	2.355002	0.432563
C	–1.887657	1.140866	–2.109954
H	–1.268414	0.434112	–2.671889
H	–2.773948	1.367849	–2.712650
H	–1.338975	2.077134	–1.973721

n-butane anion

E(scf) = –157.692482195 a.u.

C	–2.107497	–0.004808	0.000108
H	–2.192881	–0.693697	–0.878610
H	–2.192920	–0.692684	0.879655
C	–0.670625	0.504913	–0.000207
H	–0.490218	1.163215	–0.877865
H	–0.490109	1.163611	0.877103
C	0.503297	–0.516643	–0.000149
H	0.390741	–1.169930	–0.878909
H	0.390475	–1.170171	0.878397
C	1.905487	0.107292	0.000157
H	2.714258	–0.638831	–0.001247

H	–1.404979	–2.292148	0.000457
H	–3.601867	–3.393178	0.000429
C	–0.875095	1.762747	–0.000030
C	–2.077342	2.513613	–0.000191
H	0.995129	1.794771	–0.000191
H	–1.975867	3.594209	–0.000355
O	0.279297	2.474526	–0.000212
O	0.121337	–0.496982	0.000364
C	2.072393	–0.274950	1.292608
C	2.071930	–0.275380	–1.292655
C	3.483194	0.337686	1.249243
C	3.482751	0.337272	–1.249989
C	4.270966	–0.046970	–0.000446
H	4.015432	0.033315	2.157658
H	3.386991	1.431165	1.287796
H	4.014676	0.032594	–2.158481
H	3.386571	1.430743	–1.288868
H	5.238932	0.465417	–0.000695
H	4.491793	–1.120955	–0.000313
N	1.419376	0.086148	0.000029
C	2.115711	–1.783938	1.586022
H	2.337671	–1.934028	2.647486
H	1.144920	–2.240414	1.377688
H	2.879859	–2.313886	1.015140
C	1.267397	0.404264	2.409966
H	1.800215	0.294260	3.360053
H	1.132275	1.471711	2.213281
H	0.280827	–0.054350	2.520746
C	2.115106	–1.784477	–1.585584
H	2.336615	–1.934921	–2.647092
H	2.879516	–2.314228	–1.014865
H	1.144406	–2.240885	–1.376681
C	1.266474	0.403499	–2.409887
H	1.131129	1.470936	–2.213279
H	1.799132	0.293541	–3.360068
H	0.279996	–0.055373	–2.520416

TMP radical

E(scf) = –408.394983342 a.u.

C	–1.220328	–0.294422	–0.069926
C	1.220328	–0.294423	–0.069926
C	–1.251051	1.220905	–0.431455
H	2.042929	0.748256	–0.881931
H	2.043753	0.745707	0.883952

TMPO-*n*Bu

E(scf) = –641.407656225 a.u.

O	–0.621106	–0.043548	0.384952
C	1.276331	1.285628	0.016132
C	1.348443	–1.273134	0.025163
C	2.654537	1.287036	–0.668330
C	2.725539	–1.199516	–0.658172
C	3.500290	0.068158	–0.315105
H	3.171765	2.216009	–0.400023
H	2.499940	1.306189	–1.755181
H	3.294703	–2.095344	–0.381885
H	2.573233	–1.236898	–1.744879
H	4.444124	0.092452	–0.871347
H	3.769384	0.080512	0.747945
N	0.628818	–0.014150	–0.295001
C	1.397374	1.606062	1.518680
H	1.599512	2.674678	1.650660
H	0.458872	1.370560	2.026307
H	2.204572	1.059742	2.010382
C	0.421654	2.385474	–0.629596
H	0.943553	3.346723	–0.566712
H	0.237385	2.159355	–1.684502
H	–0.539071	2.491828	–0.116774
C	1.485931	–1.578891	1.529312
H	1.749033	–2.633581	1.666043
H	2.258988	–0.984767	2.020307
H	0.534829	–1.396476	2.035376
C	0.558742	–2.422889	–0.616127
H	0.353846	–2.206492	–1.669238
H	1.138114	–3.351040	–0.558005
H	–0.390306	–2.587823	–0.097246
C	–1.706253	–0.077186	–0.527332
H	–1.682009	–1.000563	–1.120859
H	–1.646284	0.766415	–1.225758
C	–2.978261	–0.003639	0.299923
H	–2.982831	–0.833115	1.018452
H	–2.963653	0.922441	0.889224
C	–4.240265	–0.053495	–0.561781
H	–4.245031	–0.982624	–1.147063

C	1.251052	1.220904	–0.431457
C	0.000001	1.942172	0.071233
H	–2.159080	1.714844	–0.045440
H	–1.277023	1.303834	–1.528656
H	2.159082	1.714843	–0.045444
H	1.277022	1.303833	–1.528658
H	0.000001	2.993698	–0.256659
H	0.000002	1.968510	1.170967
N	0.000000	–0.949101	–0.423411
C	2.326664	–0.988219	–0.889569
H	2.349730	–2.054306	–0.632134
H	2.080797	–0.912112	–1.954915
H	3.325661	–0.556634	–0.715333
C	1.653442	–0.439720	1.433194
H	1.513022	–1.487155	1.723842
H	2.705939	–0.158325	1.608317
H	1.038088	0.172834	2.101965
C	–2.326664	–0.988218	–0.889571
H	–2.080795	–0.912110	–1.954917
H	–2.349731	–2.054304	–0.632136
H	–3.325660	–0.556631	–0.715336
C	–1.653444	–0.439722	1.433193
H	–2.705941	–0.158326	1.608316
H	–1.513025	–1.487157	1.723840
H	–1.038091	0.172831	2.101967
H	–4.219544	0.768590	–1.289281
C	–5.519485	0.032272	0.268944
H	–6.411237	–0.015045	–0.364427
H	–5.574545	–0.790703	0.990511
H	–5.557696	0.971131	0.833097

TMP-O_2 (ts1)

E(scf) = –559.260225703 a.u.

ν_{min} = –406.2 cm^{-1}

C	1.311151	–0.233289	0.011557
C	–1.317952	–0.192811	0.010742
C	1.226342	–1.624233	–0.642995
C	–1.276396	–1.585183	–0.644942
C	–0.037583	–2.396231	–0.265867
H	2.128037	–2.183478	–0.368422
H	1.252880	–1.501402	–1.734603
H	–2.195390	–2.116120	–0.371777
H	–1.297497	–1.461040	–1.736544
H	–0.052103	–3.356516	–0.791503
H	–0.042136	–2.631620	0.804707

TMP-O_2 (int1)

E(scf) = –559.262884286 a.u.

C	1.323584	–0.194321	0.033667
C	–1.324088	–0.191703	0.033565
C	1.247224	–1.537618	–0.708997
C	–1.250437	–1.535212	–0.708992
C	–0.002383	–2.350886	–0.377246
H	2.159033	–2.098716	–0.475993
H	1.266675	–1.344638	–1.790464
H	–2.163310	–2.094514	–0.475850
H	–1.269596	–1.342327	–1.790481
H	–0.003272	–3.277421	–0.960619
H	–0.002672	–2.651400	0.676658
N	0.000460	0.525196	–0.163802
C	–2.384799	0.715782	–0.598305

N	0.007255	0.478070	–0.217877
C	–2.358894	0.697820	–0.676278
H	–2.319542	1.713430	–0.277064
H	–2.179483	0.755468	–1.755807
H	–3.355857	0.274240	–0.521215
C	–1.630432	–0.273999	1.507656
H	–1.456334	0.694878	1.979002
H	–2.686642	–0.533496	1.625995
H	–1.046727	–1.034794	2.028672
C	2.378907	0.624070	–0.676841
H	2.207942	0.674738	–1.758126
H	2.361718	1.644560	–0.288401
H	3.364188	0.177971	–0.510843
C	1.620412	–0.321789	1.508394
H	2.661977	–0.634696	1.627695
H	1.496060	0.657621	1.973749
H	0.998371	–1.047434	2.034900
O	0.028735	1.887837	0.496817
O	0.046588	2.801659	–0.504515
H	0.013840	0.879103	–1.162256

TMP-O_2 (ts2)

E(scf) = –559.241889305 a.u.

ν_{min} = –1506.2 cm^{-1}

C	1.308102	–0.187606	0.040638
C	–1.308080	–0.187677	0.040644
C	1.246094	–1.514587	–0.733479
C	–1.246052	–1.514625	–0.733516
C	0.000025	–2.340169	–0.424816
H	2.159676	–2.077516	–0.511153
H	1.263961	–1.292493	–1.808934
H	–2.159617	–2.077566	–0.511130
H	–1.263960	–1.292579	–1.808979
H	0.000043	–3.251655	–1.032201
H	0.000005	–2.667948	0.621242
N	–0.000011	0.517151	–0.161954
C	–2.393455	0.710153	–0.564198
H	–2.421561	1.680656	–0.065313
H	–2.212106	0.882372	–1.630127
H	–3.367770	0.222364	–0.457176
H	–2.369643	1.705575	–0.139420
H	–2.209154	0.846521	–1.670737
H	–3.369092	0.256096	–0.464354
C	–1.623800	–0.363762	1.523277
H	–1.461644	0.579928	2.047761
H	–2.676120	–0.641907	1.633350
H	–1.026578	–1.143954	1.997903
C	2.386258	0.710979	–0.598022
H	2.211118	0.841965	–1.670505
H	2.372903	1.700874	–0.139303
H	3.369600	0.249345	–0.463782
C	1.622700	–0.367094	1.523416
H	2.674183	–0.648323	1.633619
H	1.463217	0.577102	2.047802
H	1.023058	–1.145396	1.998103
O	0.001682	1.764083	0.523229
O	0.003513	2.695246	–0.581669
H	0.000972	0.963198	–1.113732

TMP-O_2

E(scf) = –559.310090671 a.u.

C	–1.300270	0.193716	0.034772
C	1.300282	0.193651	0.034770
C	–1.244889	1.526995	–0.730338
C	1.244966	1.526935	–0.730337
C	0.000058	2.351185	–0.415600
H	–2.158439	2.088951	–0.503702
H	–1.259782	1.311495	–1.806909
H	2.158543	2.088847	–0.503699
H	1.259850	1.311437	–1.806908
H	0.000079	3.268690	–1.014446
H	0.000064	2.669086	0.633782
N	–0.000012	–0.497090	–0.178316
C	2.384119	–0.694153	–0.589650
H	2.458261	–1.651517	–0.067949
H	2.161557	–0.890066	–1.643952
H	3.355001	–0.190707	–0.532110
C	1.644576	0.414238	1.518358
H	1.511903	–0.515480	2.077877

C	–1.624813	–0.407765	1.527105
H	–1.472403	0.519857	2.083796
H	–2.675374	–0.698001	1.628200
H	–1.021994	–1.194956	1.983126
C	2.393410	0.710234	–0.564320
H	2.211960	0.882442	–1.630234
H	2.421578	1.680733	–0.065435
H	3.367742	0.222453	–0.457405
C	1.624972	–0.407556	1.527092
H	2.675599	–0.697628	1.628122
H	1.472488	0.520103	2.083726
H	1.022313	–1.194801	1.983255
O	–0.000065	1.734536	0.522116
O	–0.000149	2.712813	–0.548792
H	–0.000006	1.472898	–1.377198

2-naphthol radical

E(scf) = –460.306854488 a.u.

C	–2.806548	0.676533	0.000000
C	–2.118198	–0.524687	0.000000
C	–0.715861	–0.548773	0.000000
C	0.000000	0.688607	0.000000
C	–0.732097	1.906240	0.000000
C	–2.108185	1.898996	0.000000
H	–0.542604	–2.717540	0.000000
H	–3.892316	0.677100	0.000000
H	–2.662771	–1.465240	0.000000
C	0.023876	–1.789184	0.000000
C	1.405502	0.666582	0.000000
H	–0.184680	2.844828	0.000000
H	–2.658195	2.834789	0.000000
C	2.152686	–0.569209	0.000000
C	1.374675	–1.811724	0.000000
H	1.977193	1.590317	0.000000
H	1.935603	–2.740626	0.000000
O	3.396583	–0.572989	0.000000

HOTf

E(scf) = –961.822358803 a.u.

H	1.447862	1.847013	–0.385654

H	2.692185	0.719896	1.610855
H	1.034070	1.189650	1.985775
C	–2.384152	–0.694037	–0.589643
H	–2.161601	–0.889967	–1.643945
H	–2.458341	–1.651395	–0.067939
H	–3.355009	–0.190542	–0.532103
C	–1.644547	0.414323	1.518360
H	–2.692138	0.720042	1.610860
H	–1.511924	–0.515401	2.077880
H	–1.033995	1.189702	1.985773
O	–0.000040	–1.693621	0.514363
O	–0.000085	–2.774437	–0.468711
H	–0.000056	–2.235747	–1.280297

Product 3

E(scf) = –896.344430999 a.u.

C	3.904903	–2.246871	–0.010891
C	3.828447	–1.148385	0.834864
C	2.917341	–0.116197	0.581684
C	2.070185	–0.196779	–0.534001
C	2.153153	–1.303771	–1.375358
C	3.065973	–2.322453	–1.121529
H	3.491244	1.049749	2.337082
H	4.617312	–3.040227	0.191973
H	4.482794	–1.080013	1.700215
C	2.855225	1.056228	1.454068
C	1.043189	0.880395	–0.797182
H	1.489214	–1.369784	–2.233657
H	3.121076	–3.176397	–1.789634
C	1.260895	2.191671	–0.020819
C	2.091307	2.129230	1.190737
H	1.080341	1.158299	–1.856502
H	2.093439	3.010058	1.824349
O	0.725056	3.217533	–0.392554
C	–0.383955	0.424073	–0.494221
C	–1.449056	1.063525	–1.136638
C	–0.651694	–0.575162	0.444183
C	–2.760896	0.704236	–0.863908
H	–1.244189	1.860519	–1.843736
C	–1.959984	–0.943967	0.730655

O	1.238957	1.060426	–0.917491
S	0.846244	–0.143605	0.078037
O	1.208067	–1.375123	–0.564612
O	1.248502	0.214441	1.417576
C	–0.996131	0.006981	–0.001751
F	–1.526810	–0.886477	0.817348
F	–1.414098	–0.208897	–1.236664
F	–1.345223	1.229910	0.381959

Product 3 radical

E(scf) = –895.718156982 a.u.

C	3.983703	–2.355699	–0.264528
C	4.338575	–1.041753	–0.011455
C	3.368262	–0.033958	0.067775
C	1.988051	–0.369201	–0.093008
C	1.658829	–1.721363	–0.382651
C	2.634697	–2.690776	–0.462800
H	4.808849	1.557228	0.421144
H	4.748726	–3.123358	–0.324043
H	5.383799	–0.776876	0.123552
C	3.751303	1.340081	0.292721
C	1.003486	0.655437	0.004909
H	0.623081	–1.986314	–0.562670
H	2.359577	–3.716239	–0.688167
C	1.410528	2.053839	0.167380
C	2.838202	2.329850	0.323799
H	3.111177	3.368982	0.473617
O	0.578560	2.973386	0.193882
C	–0.442375	0.362457	–0.007213
C	–1.309197	1.053792	–0.865961
C	–0.977446	–0.588276	0.875369
C	–2.670213	0.786508	–0.862543
H	–0.911998	1.802661	–1.539546
C	–2.338194	–0.857526	0.896501
H	–0.320249	–1.106383	1.566456
C	–3.163237	–0.165990	0.019990
H	–3.349715	1.306823	–1.525874
H	–2.762442	–1.582572	1.579674
N	–4.603842	–0.448615	0.029658
O	–5.307709	0.173060	–0.750448
O	–5.010068	–1.289970	0.816384

H	0.165635	–1.077624	0.950964
C	–2.994925	–0.299875	0.067368
H	–3.595728	1.186101	–1.357291
H	–2.185078	–1.717427	1.454321
N	–4.378580	–0.691159	0.359245
O	–5.269908	–0.117323	–0.247045
O	–4.555980	–1.567774	1.191241

Product 4

E(scf) = –1331.75722858 a.u.

C	5.081742	2.527569	–1.203351
C	4.179704	3.279525	–0.496819
C	3.004705	2.691654	0.039365
C	2.771515	1.300479	–0.156785
C	3.721900	0.549820	–0.899275
C	4.845909	1.149005	–1.408004
H	2.245718	4.519966	0.918902
H	5.976473	2.987510	–1.611107
H	4.350202	4.340981	–0.337843
C	2.058049	3.460962	0.765843
C	1.585300	0.706974	0.380931
H	3.547674	–0.507785	–1.067367
H	5.559650	0.560621	–1.976394
C	0.702104	1.503879	1.068957
C	0.926572	2.883002	1.271763
H	0.197021	3.453547	1.836944
O	–0.414697	0.929829	1.649400
C	1.317532	–0.750997	0.230095
C	0.355597	–1.215581	–0.671210
C	2.040148	–1.672861	0.994888
C	0.108791	–2.575839	–0.806558
H	–0.207318	–0.507545	–1.270711
C	1.807820	–3.035524	0.869653
H	2.788272	–1.314796	1.695089
C	0.842187	–3.463913	–0.031951
H	–0.634020	–2.950952	–1.499469
H	2.357344	–3.761153	1.456180
N	0.591119	–4.904394	–0.172237
O	–0.263227	–5.254367	–0.970786
O	1.253268	–5.663121	0.518257
C	–1.606531	0.997996	1.000139

C	–2.679386	0.366203	1.637455
C	–1.784488	1.637502	–0.229927
C	–3.929670	0.370274	1.046648
H	–2.508119	–0.124256	2.588930
C	–3.040271	1.639028	–0.822152
H	–0.952039	2.128378	–0.720913
C	–4.096376	1.007007	–0.180502
H	–4.776569	–0.113085	1.517655
H	–3.207755	2.124754	–1.775636
N	–5.415610	1.010438	–0.809153
O	–5.527866	1.569369	–1.890343
O	–6.327756	0.453854	–0.216494

Iodobenzene

E(scf) = –242.953524966 a.u.

I	1.550235	–0.000002	0.000010
C	0.561101	0.000024	–0.000041
C	1.245164	–1.212887	–0.000020
C	1.245151	1.212888	–0.000055
C	2.638121	–1.204353	–0.000024
H	0.704247	–2.152828	–0.000153
C	2.638125	1.204356	0.000009
H	0.704231	2.152838	–0.000207
C	3.335916	–0.000008	0.000066
H	3.175486	–2.147885	0.000024
H	3.175484	2.147881	0.000083
H	4.421555	–0.000013	0.000091

Ph_2IOTf

E(scf) = –1640.11658906 a.u.

I	–0.020901	–0.519891	0.301127
C	0.013579	1.612201	0.240246
C	0.994644	2.265444	0.975282
C	–0.922955	2.267139	–0.545054
C	1.041780	3.655144	0.905268
H	1.709311	1.722367	1.584168
C	–0.850844	3.658800	–0.594292
H	–1.701712	1.735951	–1.085054
C	0.123252	4.348694	0.121251
H	1.796530	4.190409	1.472390
H	–1.570860	4.197617	–1.201384
H	0.166032	5.432027	0.070991
O	–2.406647	–0.114033	0.556745
S	–3.253101	–0.591314	–0.604104
O	–3.463088	0.452421	–1.603897
O	–2.841827	–1.908689	–1.076059
C	–4.884850	–0.825922	0.220409
F	–5.776883	–1.242620	–0.673563
F	–5.305988	0.319412	0.750153
F	–4.784555	–1.736205	1.186493
C	2.097111	–0.597631	0.098174
C	2.691547	–0.167383	–1.088531
C	2.862822	–1.101324	1.148363
C	4.072287	–0.232522	–1.224591

PhIOTf radical

E(scf) = –1204.11857149 a.u.

I	1.076821	–1.568068	–0.003528
C	2.221057	0.188853	0.005725
C	3.373387	0.226819	0.787598
C	1.800637	1.265329	–0.770947
C	4.141647	1.387340	0.771295
H	3.668832	–0.619466	1.398402
C	2.580476	2.418313	–0.758568
H	0.877611	1.223581	–1.341077
C	3.746394	2.478548	0.001459
H	5.045572	1.436697	1.369881
H	2.266211	3.273484	–1.348054
H	4.346106	3.383325	–0.000534
O	–0.811378	–0.133946	0.684803
S	–1.836043	–0.041437	–0.468881
O	–1.411045	0.890498	–1.503637
O	–2.335334	–1.346250	–0.865386
C	–3.199920	0.803064	0.457399
F	–4.208716	1.010448	–0.380775
F	–2.779688	1.968711	0.933211
F	–3.613724	0.040950	1.460947

H	2.088601	0.222823	−1.902062
C	4.246194	−1.175227	1.014205
H	2.396641	−1.432818	2.071000
C	4.821383	−0.735841	−0.168592
H	4.568507	0.094833	−2.129868
H	4.874235	−1.559276	1.808627
N	6.286259	−0.805023	−0.309862
O	6.919237	−1.251034	0.632078
O	6.767000	−0.409694	−1.358167

IPh OTf− OTMP+

E(scf) = −1687.76424204 a.u.

I	−4.293595	0.339686	−0.981254
C	−3.227419	−0.047756	0.802606
C	−3.563864	−1.164099	1.564778
C	−2.178509	0.792495	1.165738
C	−2.826844	−1.441237	2.714705
H	−4.384976	−1.809333	1.271280
C	−1.444899	0.495532	2.313653
H	−1.897220	1.650170	0.563273
C	−1.766787	−0.617230	3.086428
H	−3.087379	−2.306676	3.317805
H	−0.602223	1.128620	2.572262
H	−1.191617	−0.841196	3.980034
O	0.152665	2.804445	0.059619
S	1.308358	1.924210	0.173933
O	1.639703	1.448085	1.525848
O	1.377741	0.846882	−0.846223
C	2.751999	2.977748	−0.275377
F	3.881574	2.244800	−0.246142
F	2.893151	3.984384	0.581572
F	2.619202	3.474121	−1.503585
O	1.316206	−1.171612	1.225259
C	0.844617	−2.183421	−0.821689
C	3.297846	−1.646932	0.112523
C	1.194920	−3.686592	−0.640268
C	3.522735	−3.182871	0.184356
C	2.683793	−3.991061	−0.804431
H	0.595800	−4.234231	−1.374936
H	0.856846	−4.004246	0.354567
H	4.592904	−3.347798	0.021897
H	3.296855	−3.510362	1.207099

HO(TMP)$_2$ cation 2

E(scf) = −892.391259698 a.u.

O	0.049595	0.115415	−0.520093
C	−1.868193	1.141493	0.565322
C	−1.930216	−1.065535	−0.755230
C	−3.176121	0.703544	1.259279
C	−3.239049	−1.425209	−0.029684
C	−4.046173	−0.209284	0.405460
H	−3.721803	1.611703	1.537947
H	−2.916395	0.184546	2.190790
H	−3.827507	−2.064714	−0.696089
H	−2.988224	−2.025904	0.854106
H	−4.918334	−0.530172	0.982969
H	−4.438711	0.327583	−0.465118
N	−1.187174	−0.138188	0.174123
C	−2.148035	2.155306	−0.560748
H	−2.249431	3.154672	−0.126193
H	−1.334195	2.187636	−1.286643
H	−3.073754	1.951200	−1.098355
C	−1.030679	1.864868	1.637824
H	−1.667399	2.585901	2.158175
H	−0.646764	1.173909	2.395308
H	−0.215539	2.464807	1.209671
C	−2.199848	−0.486740	−2.158795
H	−2.523577	−1.297658	−2.817755
H	−2.982132	0.270054	−2.177357
H	−1.292266	−0.056602	−2.592556
C	−1.132678	−2.359579	−0.961532
H	−0.862242	−2.846958	−0.022622
H	−1.758279	−3.062692	−1.518502
H	−0.231491	−2.186758	−1.554420
C	2.182951	1.016000	−0.726174

H	2.854049	−5.057601	−0.625448
H	3.006153	−3.799306	−1.833032
N	1.780028	−1.522275	0.190660
C	1.057911	−1.717792	−2.265951
H	0.194344	−2.075587	−2.833588
H	1.082403	−0.629832	−2.317832
H	1.947894	−2.135056	−2.734391
C	−0.581915	−1.875321	−0.391482
H	−1.260460	−2.413557	−1.058517
H	−0.796240	−2.174161	0.636431
H	−0.772761	−0.804639	−0.489354
C	3.867710	−1.022658	−1.165821
H	4.939980	−0.886824	−1.002218
H	3.747393	−1.652290	−2.046266
H	3.416110	−0.046667	−1.345247
C	3.881087	−0.952712	1.339004
H	3.512072	−1.384508	2.271481
H	4.966487	−1.080972	1.303678
H	3.640056	0.112568	1.334656

TMPO-*n*Bu_H cation 1

E(scf) = –641.799405639 a.u.

O	−0.637245	−0.000038	0.371807
C	1.341247	1.338453	0.035409
C	1.341316	−1.338434	0.035416
C	2.714751	1.251153	−0.647464
C	2.714815	−1.251059	−0.647459
C	3.510405	0.000064	−0.286001
H	3.264001	2.158048	−0.375634
H	2.576708	1.291274	−1.737390
H	3.264106	−2.157931	−0.375633
H	2.576767	−1.291189	−1.737385

C	1.745509	−0.857159	1.118449
C	2.713159	−0.104721	−1.626159
C	2.320700	−1.874388	0.130921
C	3.319208	−1.282631	−0.864258
H	3.455987	0.342840	−2.294636
H	1.893166	−0.460597	−2.260023
H	2.798275	−2.660984	0.724233
H	1.497285	−2.348291	−0.408548
H	3.610462	−2.056643	−1.579553
H	4.242150	−0.981684	−0.357041
N	1.199924	0.351627	0.299484
C	0.646129	−1.470577	1.992566
H	0.323474	−0.776896	2.774675
H	−0.230022	−1.786830	1.443570
H	1.089730	−2.339688	2.487179
C	2.807196	−0.332780	2.103240
H	2.500939	0.600407	2.588575
H	2.909825	−1.079389	2.894155
H	3.793471	−0.196188	1.668555
C	1.401227	2.047929	−1.548834
H	0.642872	1.597444	−2.185766
H	0.933507	2.805584	−0.911308
H	2.118466	2.562188	−2.193701
C	3.289926	1.779411	0.014629
H	3.730762	2.482807	−0.695335
H	2.896689	2.372692	0.847036
H	4.099572	1.154193	0.380866
H	0.925337	1.071412	0.971546

TMPO-*n*Bu_H cation 2

E(scf) = –641.746061829 a.u.

O	0.613544	0.271668	0.380148
C	−1.170683	−1.317913	0.011021
C	−1.517164	1.270620	0.033782
C	−2.536990	−1.433755	−0.699253
C	−2.862664	1.039000	−0.684434
C	−3.502562	−0.305262	−0.353405
H	−2.957426	−2.410444	−0.439024
H	−2.359911	−1.437922	−1.781146
H	−3.523078	1.868080	−0.411129
H	−2.696287	1.104062	−1.766255

H	4.454093	0.000084	−0.838065
H	3.778328	0.000080	0.775557
N	0.621439	−0.000008	−0.253238
C	1.432036	1.594905	1.537959
H	1.671424	2.652238	1.679148
H	0.478386	1.400349	2.032285
H	2.214359	1.017882	2.029199
C	0.492417	2.429957	−0.619711
H	1.038683	3.374598	−0.556954
H	0.311747	2.234206	−1.683553
H	−0.463193	2.563895	−0.106875
C	1.432126	−1.594887	1.537964
H	1.671467	−2.652231	1.679154
H	2.214490	−1.017901	2.029185
H	0.478500	−1.400292	2.032322
C	0.492531	−2.429969	−0.619708
H	0.311790	−2.234187	−1.683533
H	1.038858	−3.374581	−0.557017
H	−0.463043	−2.563994	−0.106829
C	−1.753030	−0.000141	−0.549854
H	−1.704264	−0.895177	−1.181116
H	−1.704263	0.894746	−1.181326
C	−3.005406	−0.000035	0.303299
H	−2.993809	−0.882449	0.953777
H	−2.993702	0.882427	0.953712
C	−4.272955	0.000016	−0.557955
H	−4.271194	−0.879921	−1.213789
H	−4.271138	0.879988	−1.213741
C	−5.539595	0.000031	0.295974
H	−6.433196	0.000124	−0.333881
H	−5.581819	−0.885620	0.938777
H	−5.581721	0.885600	0.938898
H	0.461568	−0.000024	−1.268304

H	−4.423285	−0.426249	−0.930972
H	−3.797107	−0.351279	0.700954
N	−0.700636	0.064038	−0.303996
C	−1.299353	−1.657926	1.508341
H	−1.481607	−2.731472	1.605210
H	−0.387736	−1.463900	2.094755
H	−2.117920	−1.135888	2.000856
C	−0.199996	−2.312458	−0.642034
H	−0.634699	−3.314894	−0.602722
H	−0.027010	−2.059329	−1.691476
H	0.765522	−2.373289	−0.123258
C	−1.715229	1.542483	1.532340
H	−2.121222	2.550922	1.651488
H	−2.415877	0.857848	2.011001
H	−0.767405	1.513919	2.078807
C	−0.815271	2.474756	−0.605358
H	−0.560225	2.268435	−1.649118
H	−1.486099	3.338246	−0.584226
H	0.089806	2.756962	−0.057112
C	1.798421	0.297775	−0.529997
H	1.741123	1.283749	−0.987543
H	1.635206	−0.476413	−1.278028
C	3.036710	0.088728	0.308675
H	3.096571	0.859343	1.085852
H	2.989698	−0.890850	0.806711
C	4.295883	0.138708	−0.570634
H	4.349343	1.112964	−1.070530
H	4.219477	−0.619952	−1.358793
C	5.565699	−0.092036	0.246117
H	6.447543	−0.052196	−0.398236
H	5.682185	0.672171	1.021510
H	5.551196	−1.072335	0.733714
H	0.724525	−0.388220	1.094534

4. Copies of ^1H and ^{13}C of compounds

1,1'-oxybis(2,2,6,6-tetramethylpiperidine)

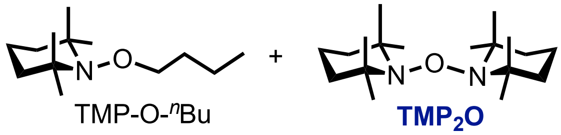

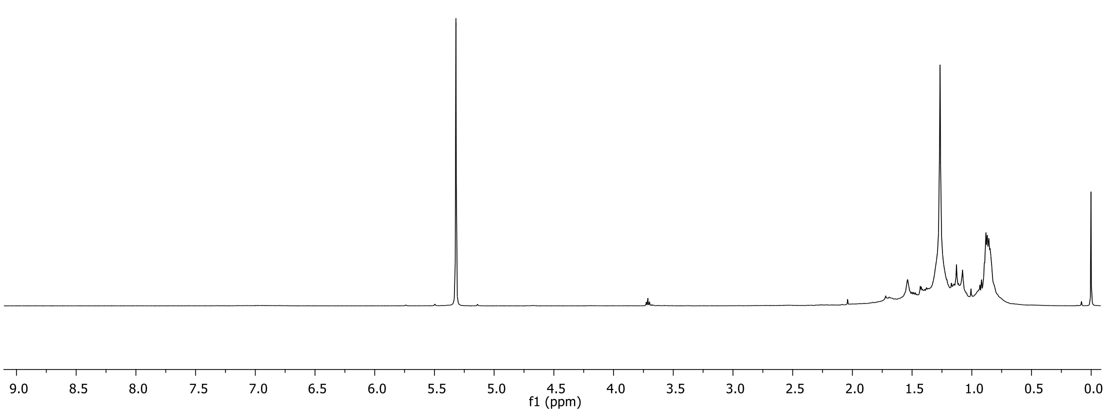

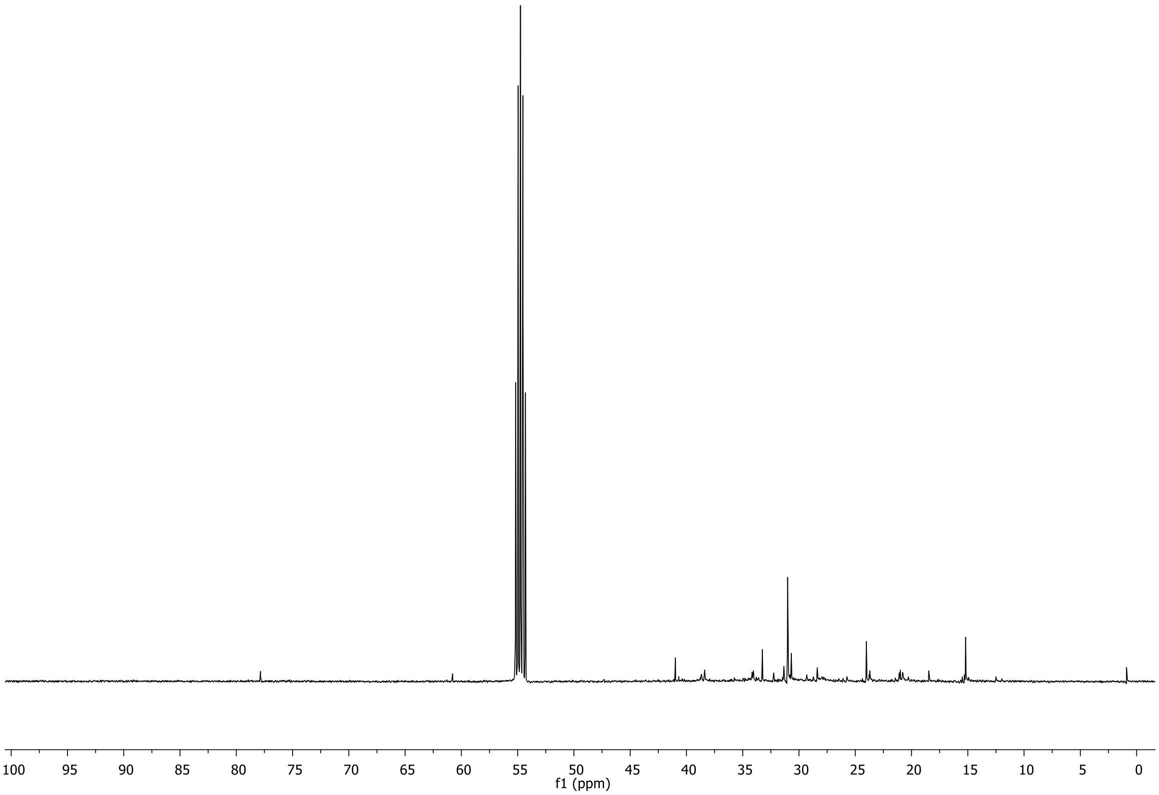

2-phenoxy-1-phenylnaphthalene (2a)

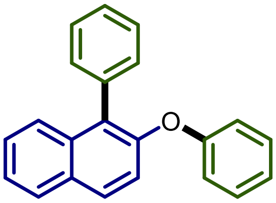

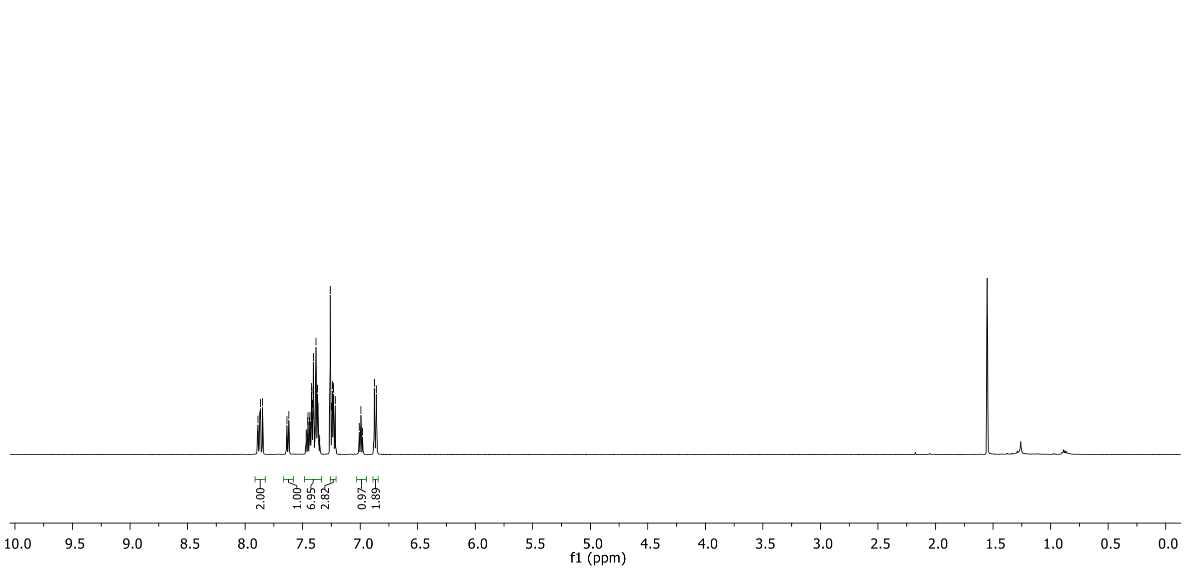

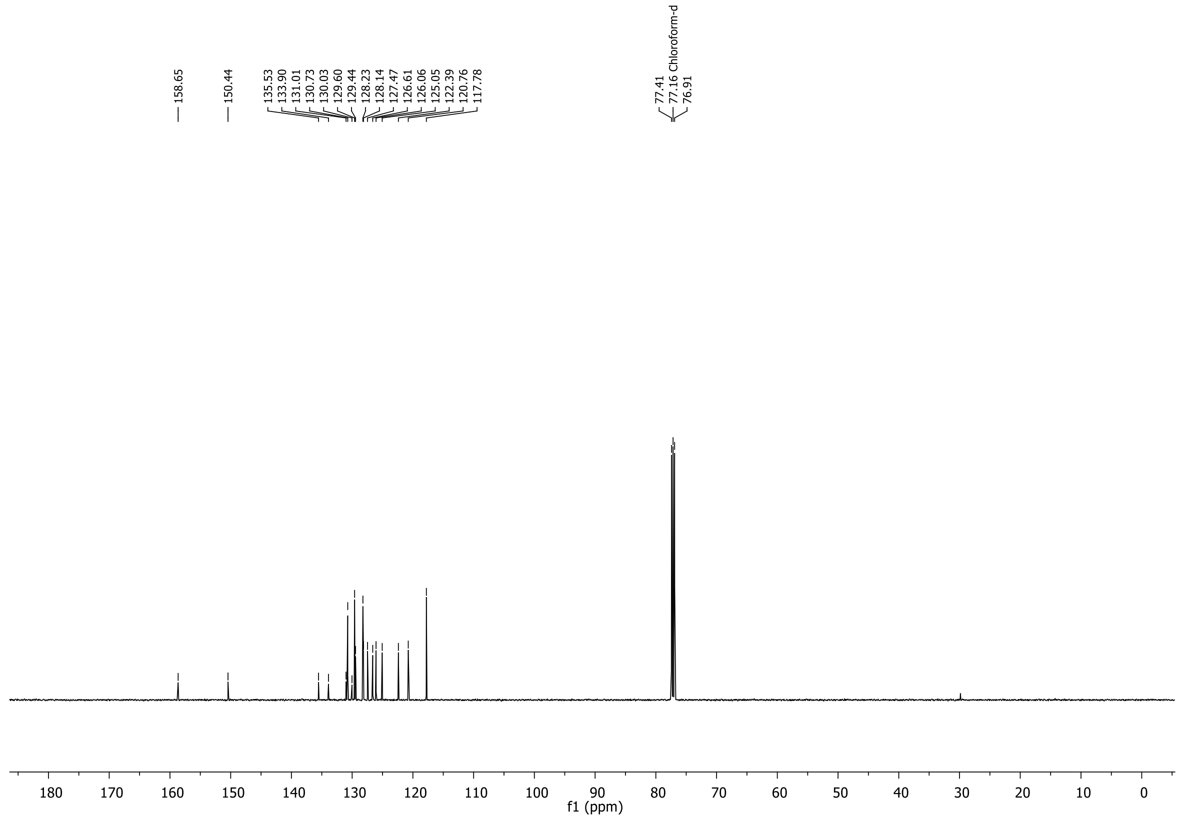

1-phenoxy-2-phenylnaphthalene (2b)

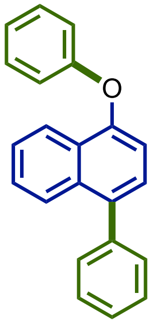

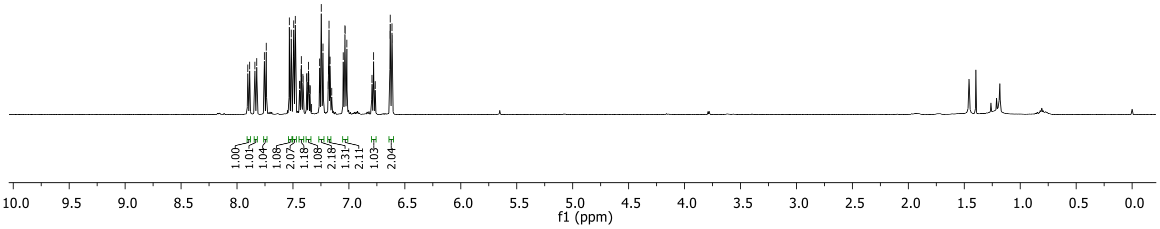

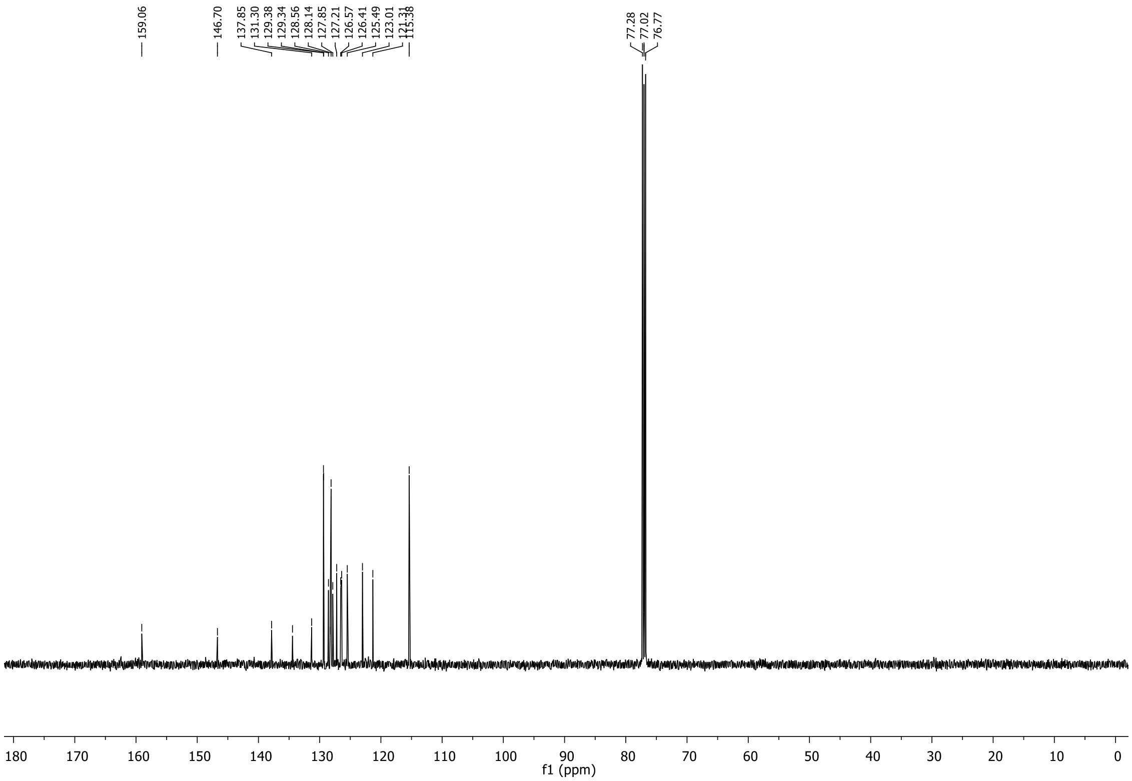

4-bromo-1-phenoxy-2-phenylnaphthalene (2c)

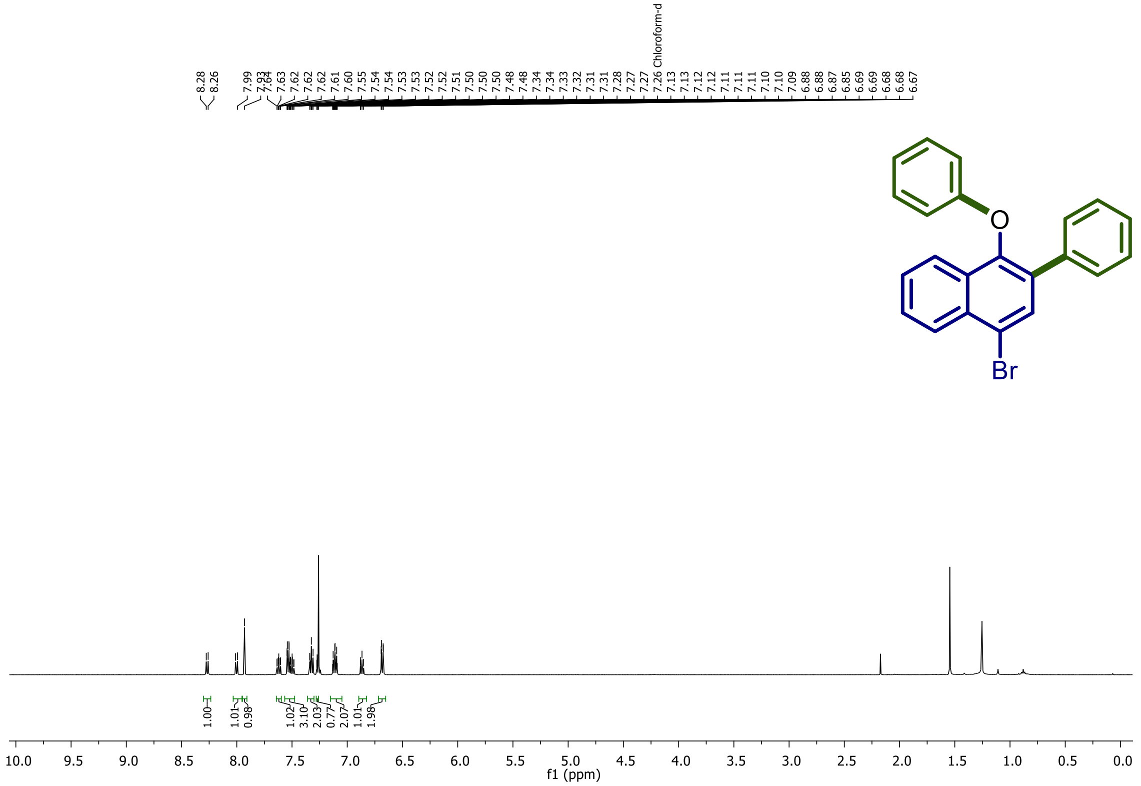

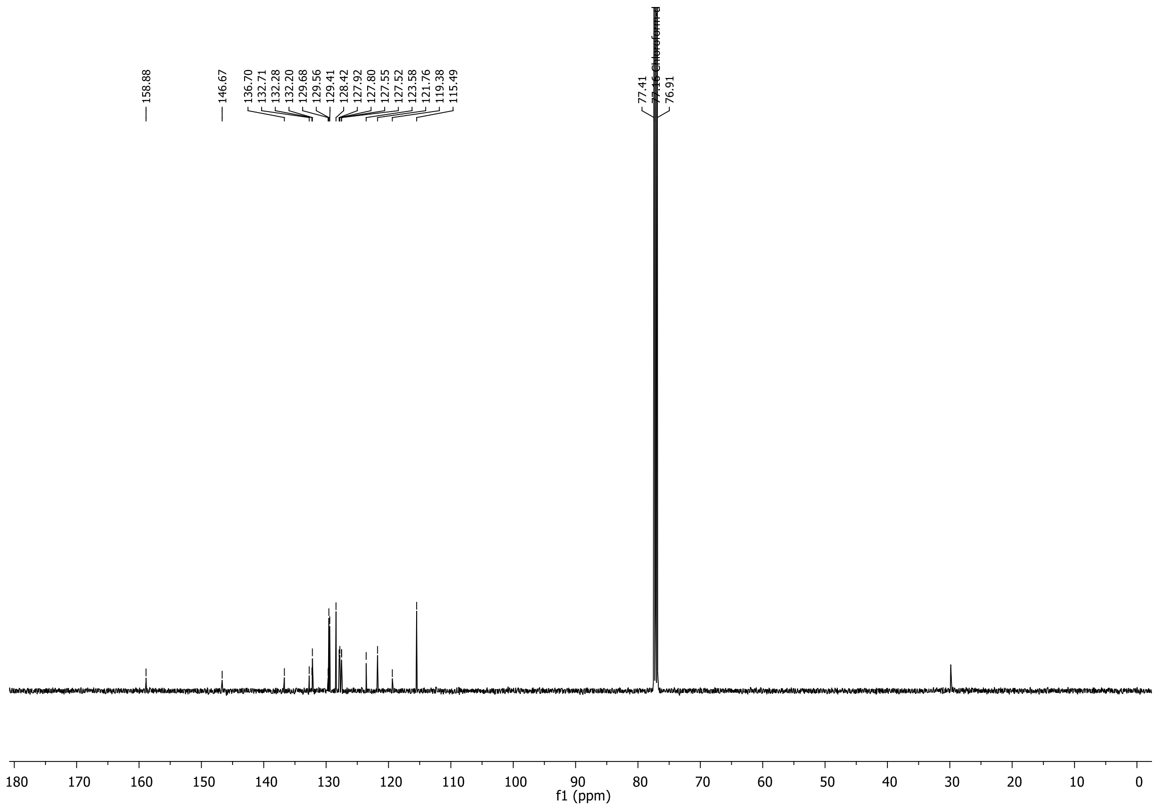

6-bromo-2-phenoxy-1-phenylnaphthalene (2d)

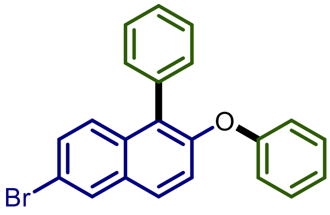

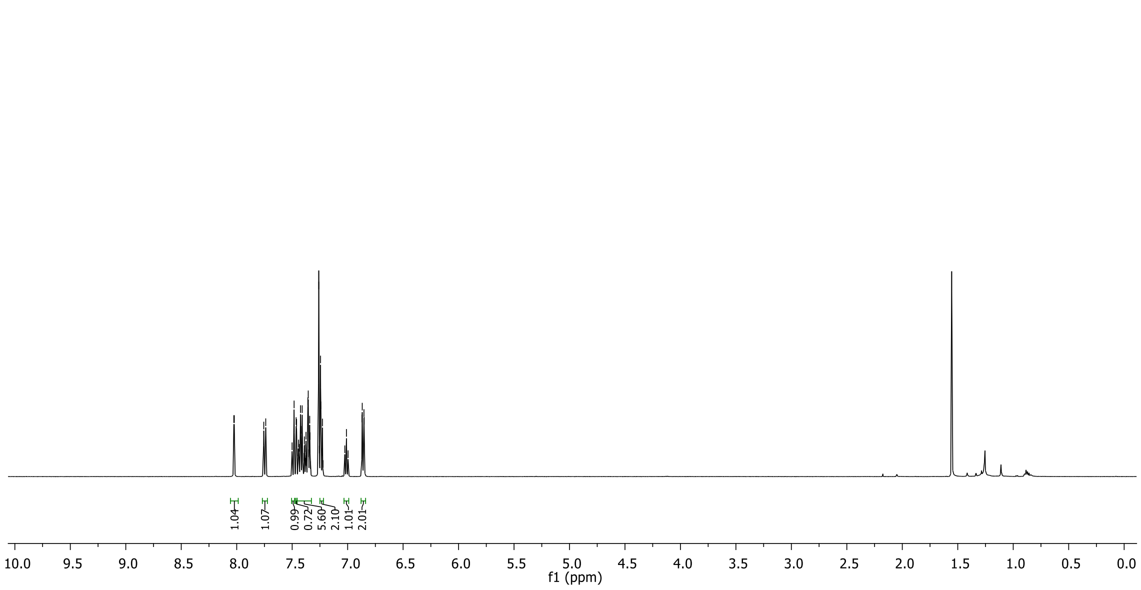

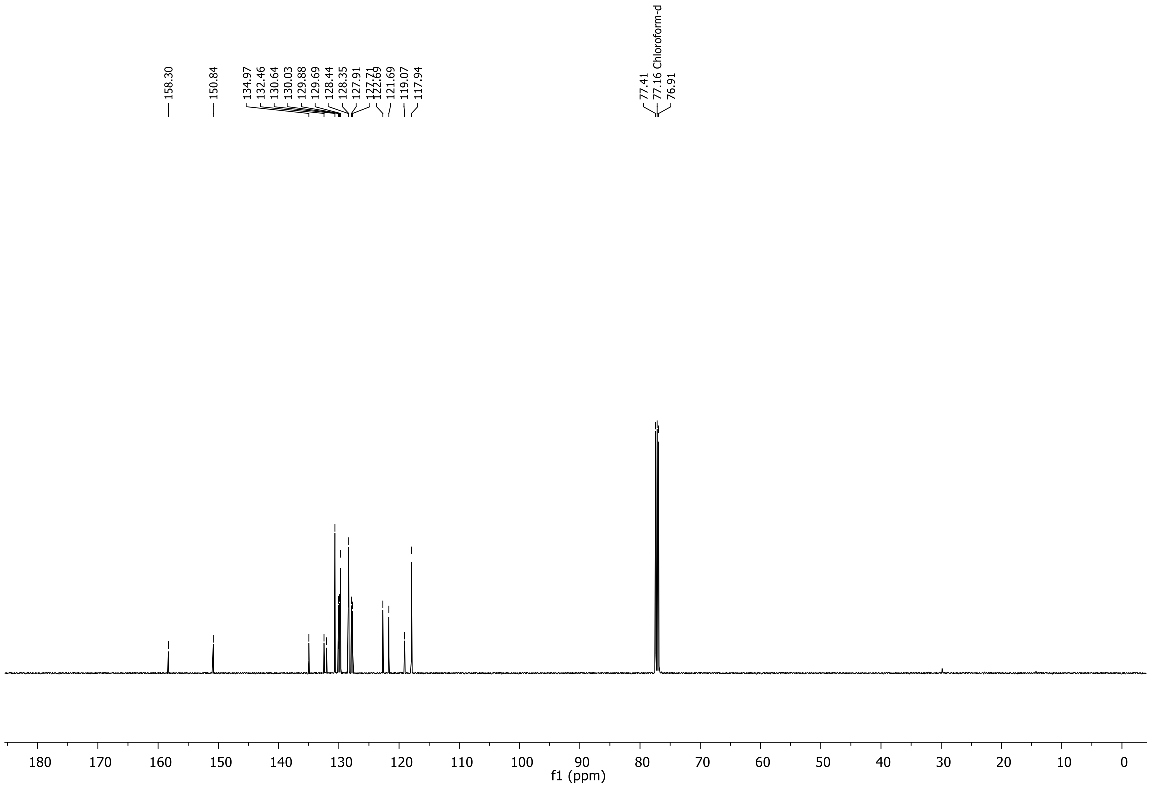

3-bromo-2-phenoxy-1-phenylnaphthalene (2e)

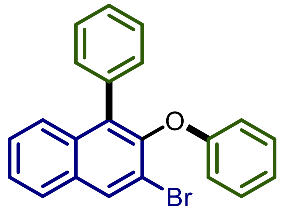

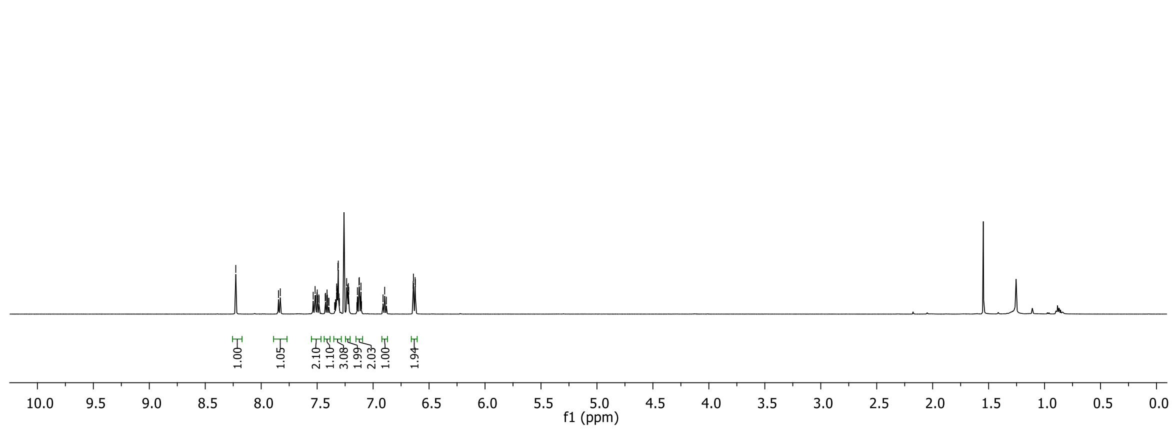

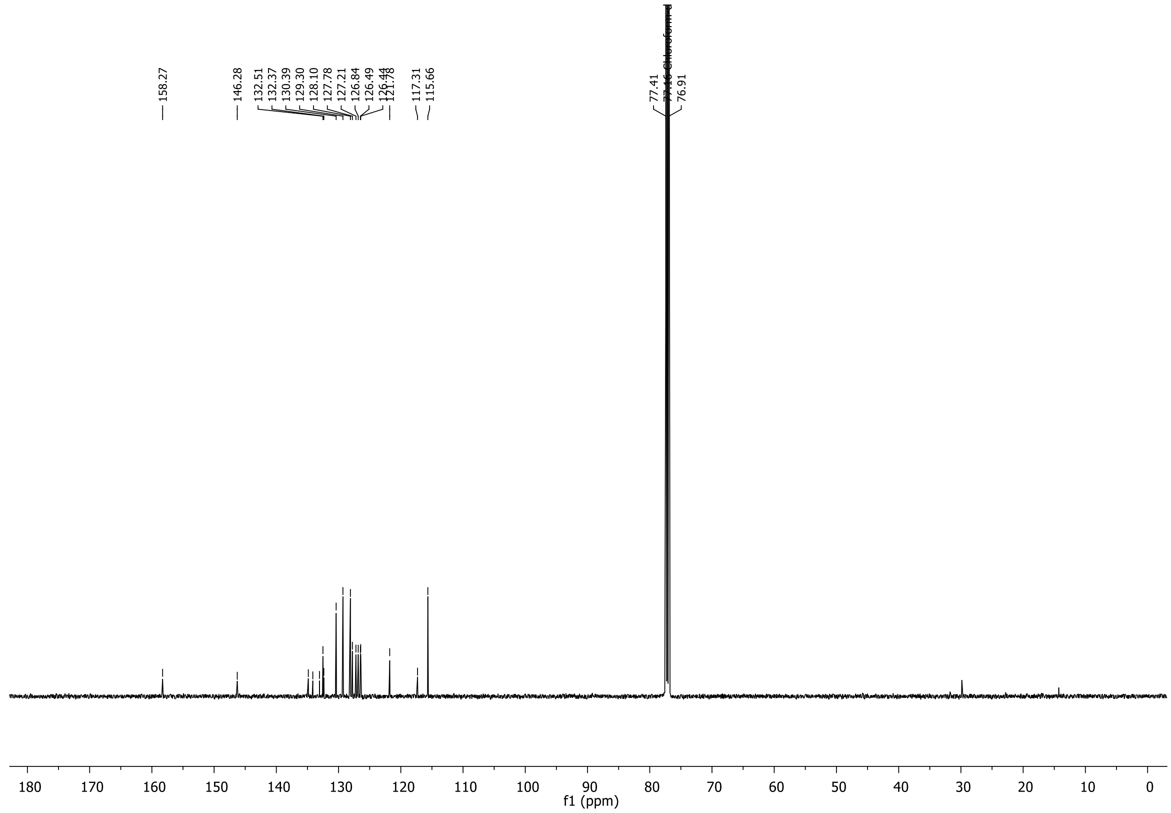

2-phenoxy-1-phenyl-6-(*p*-tolyl)naphthalene (2f)

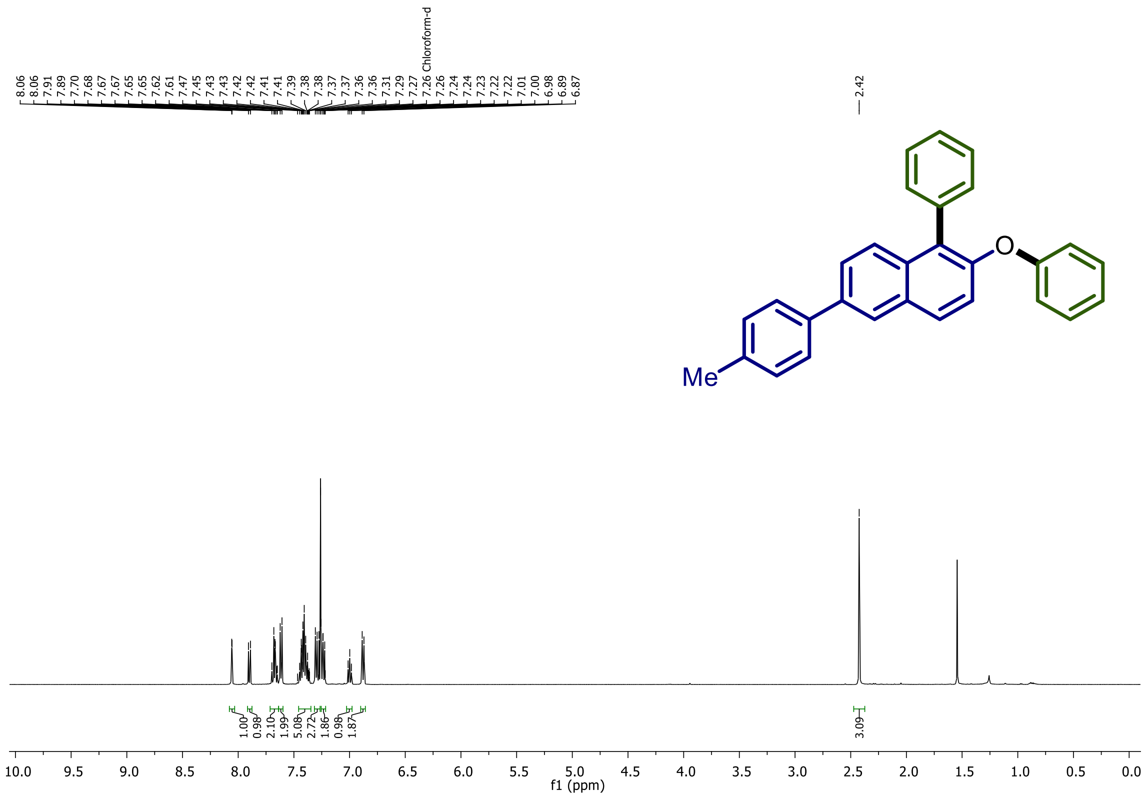

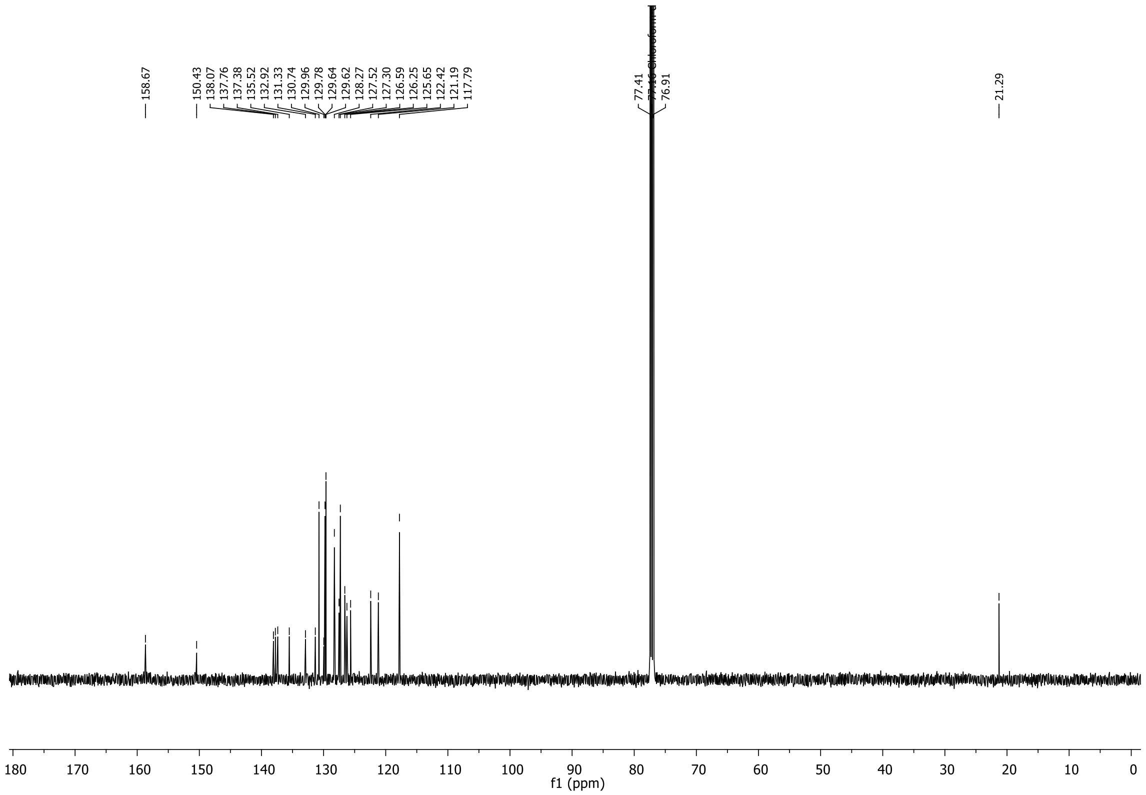

7-methoxy-2-phenoxy-1-phenylnaphthalene (2g)

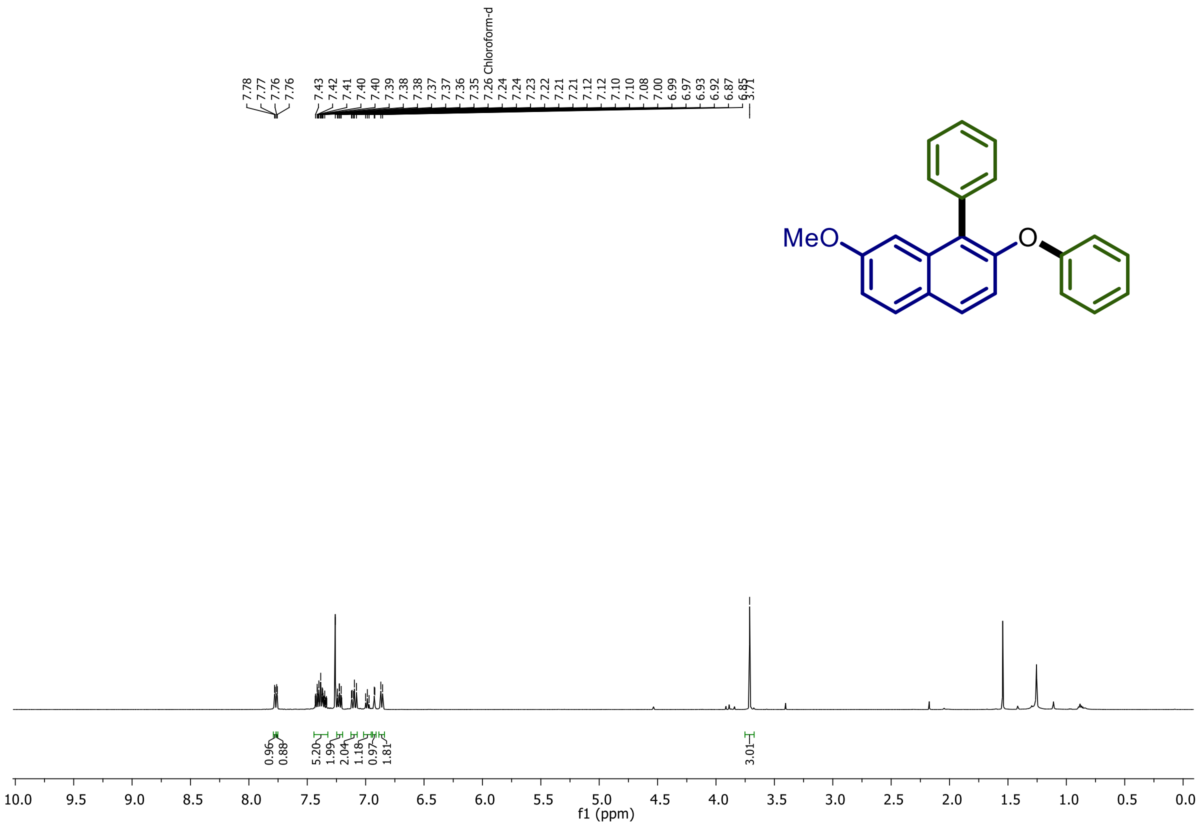

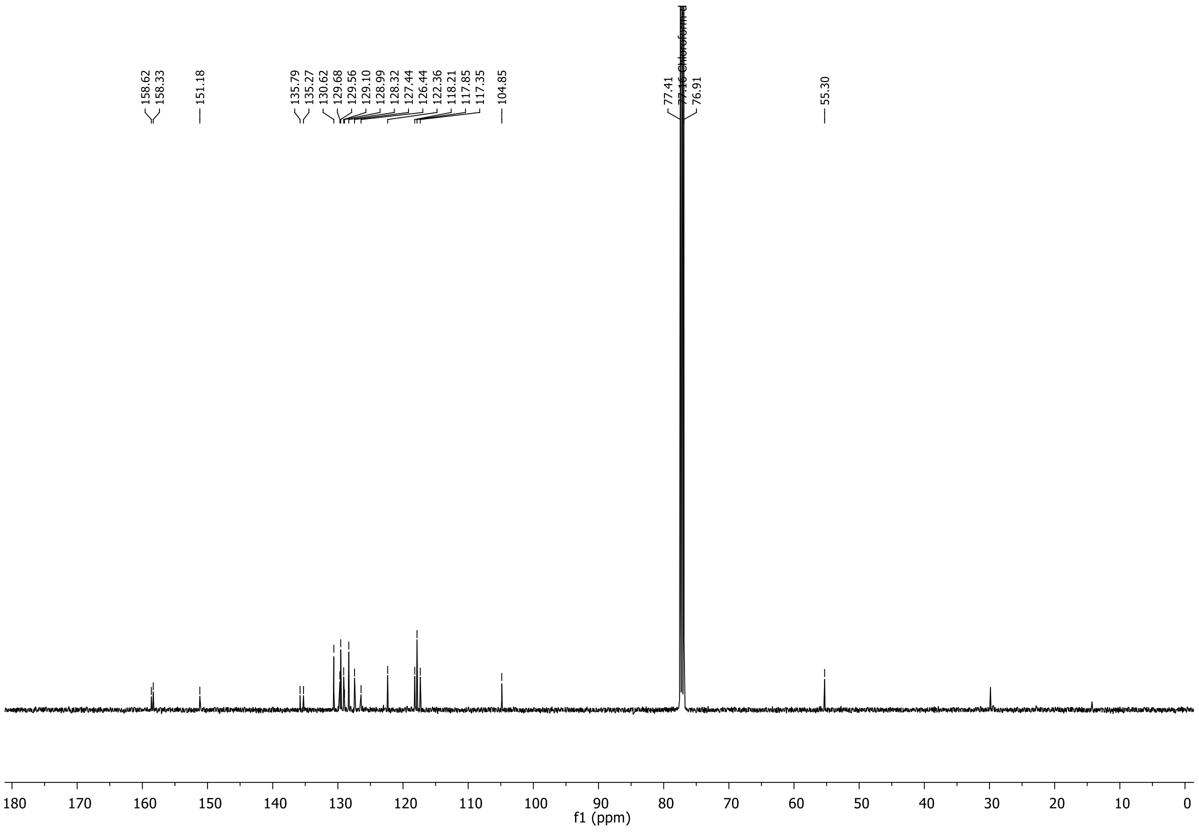

2-phenoxy-1,6-diphenylnaphthalene (2h)

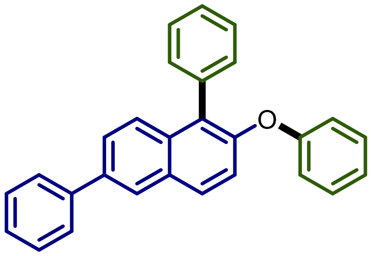

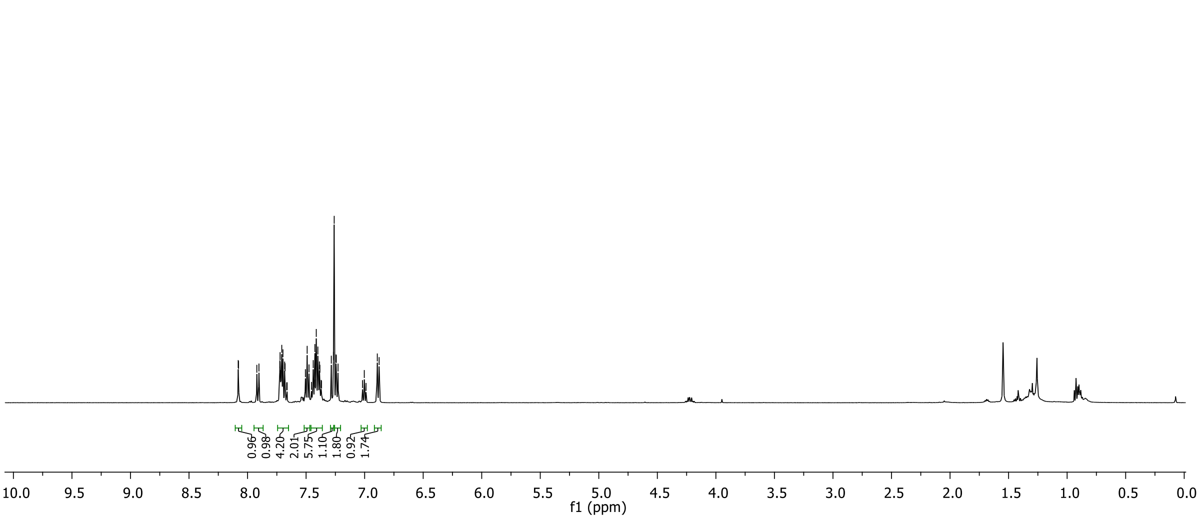

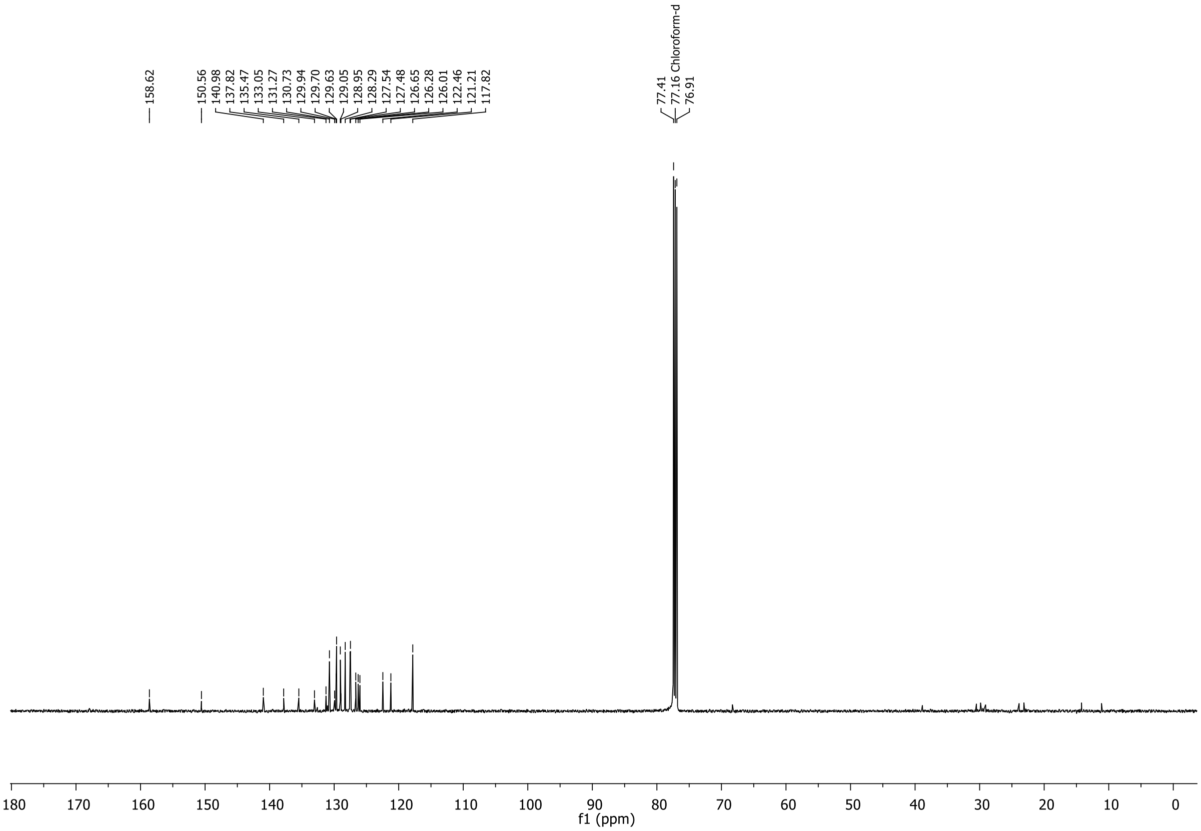

6-phenoxy-5-phenyl-2,2'-binaphthalene (2i)

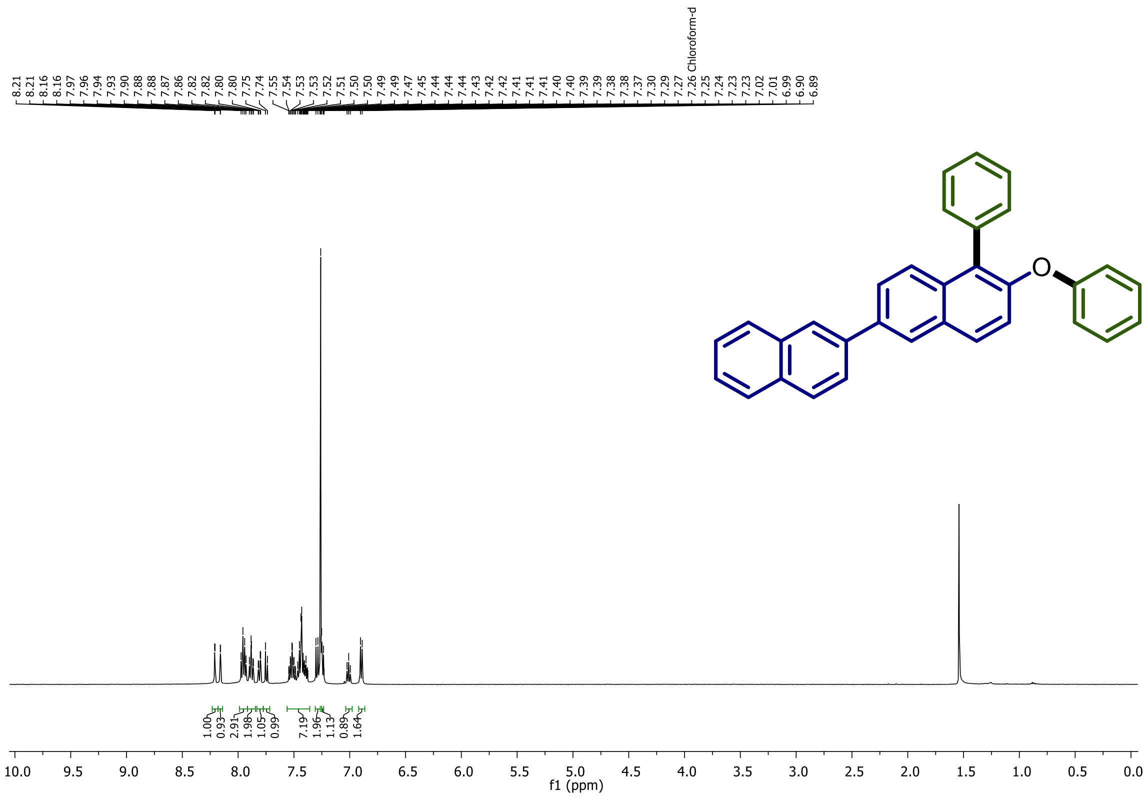

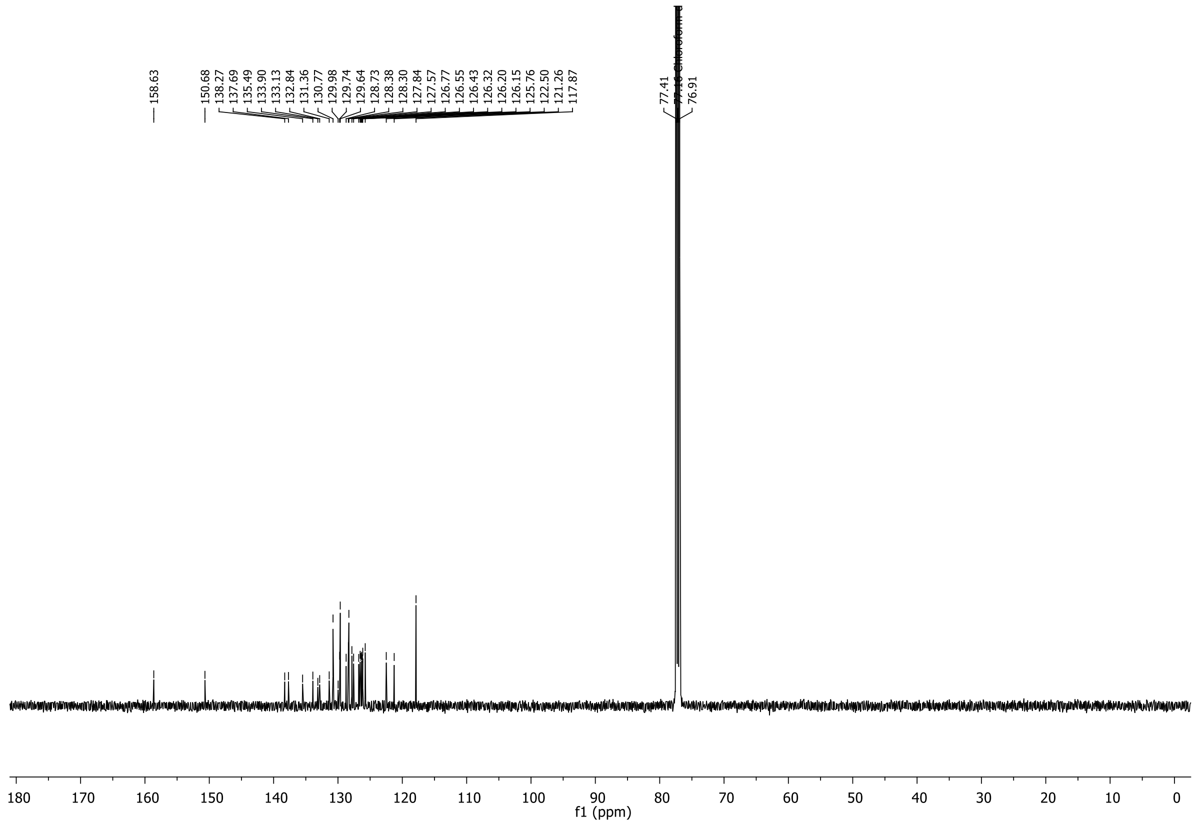

6-(4-fluorophenyl)-2-phenoxy-1-phenylnaphthalene (2j)

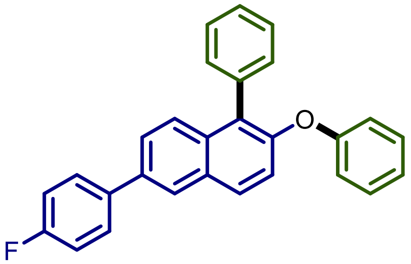

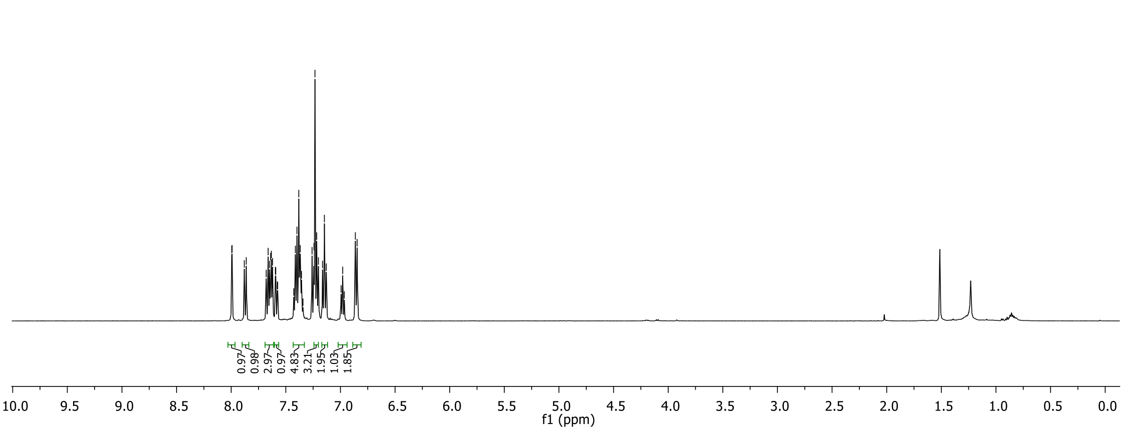

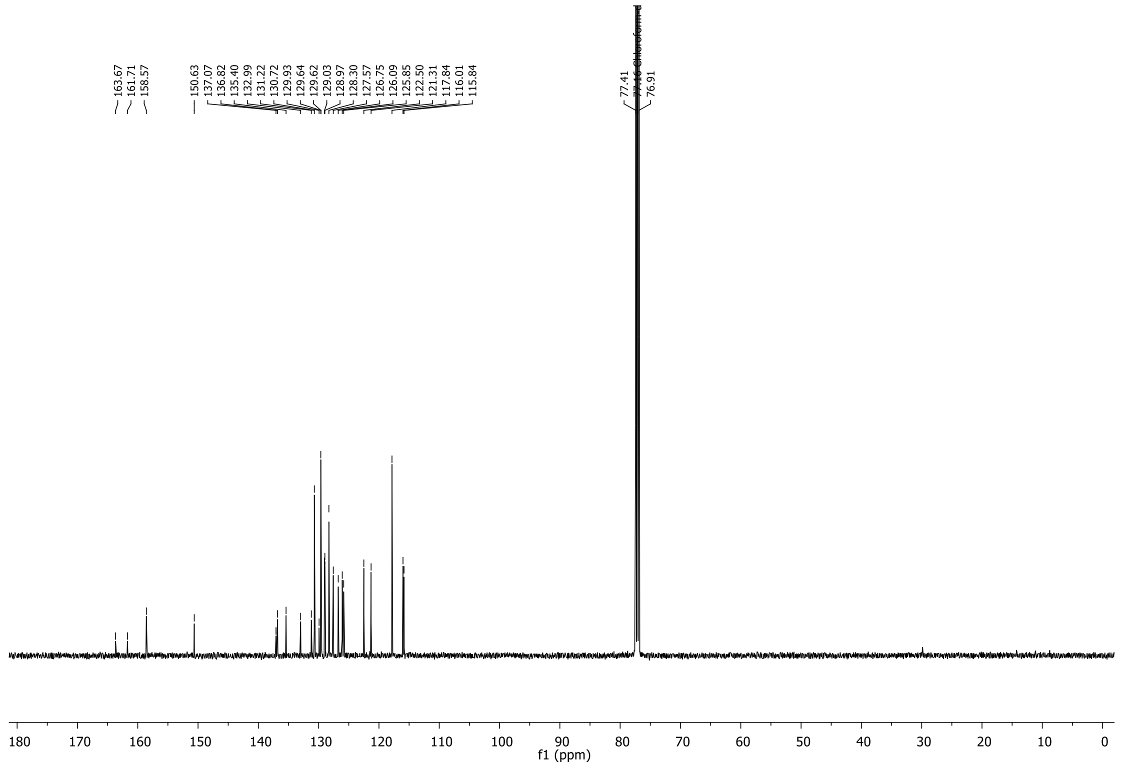

6-(4-chlorophenyl)-2-phenoxy-1-phenylnaphthalene (2k)

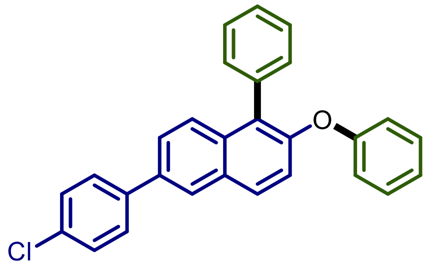

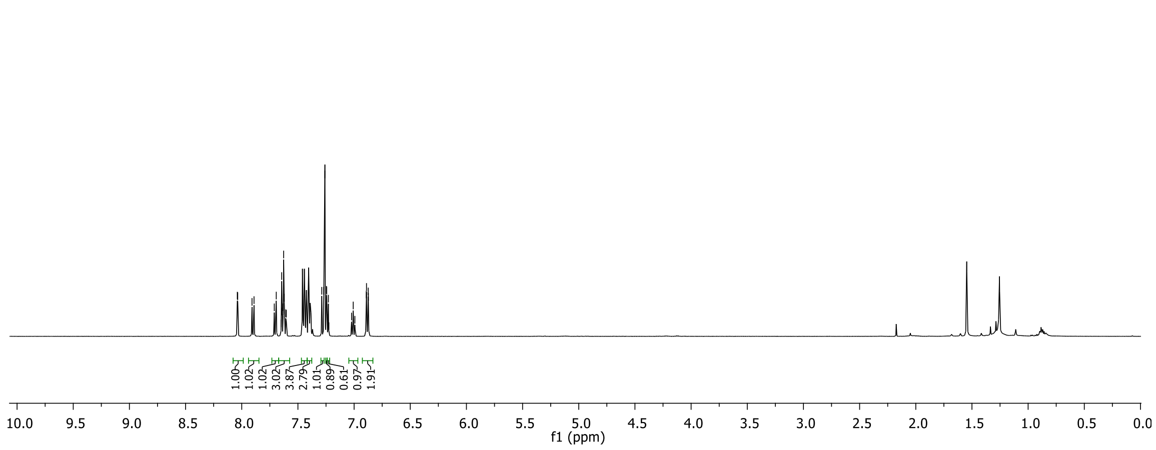

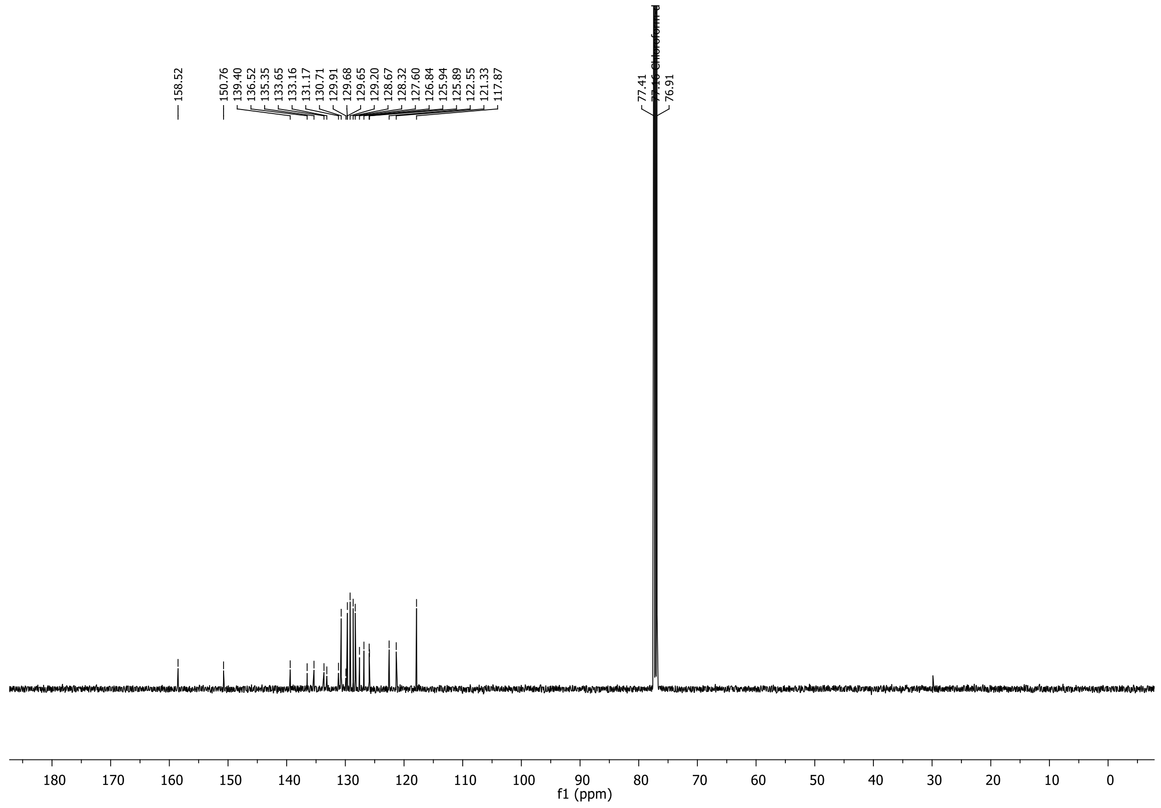

6-(3-chloro-4-fluorophenyl)-2-phenoxy-1-phenylnaphthalene (2l)

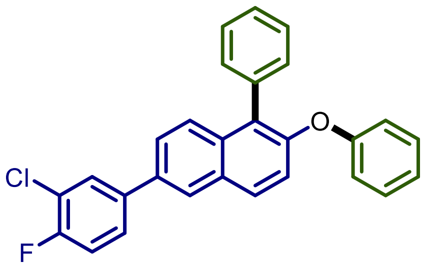

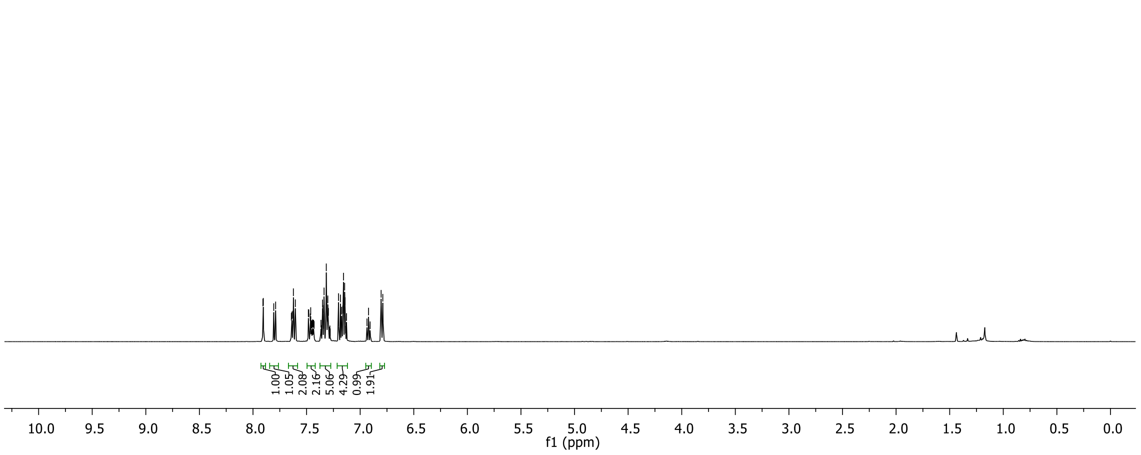

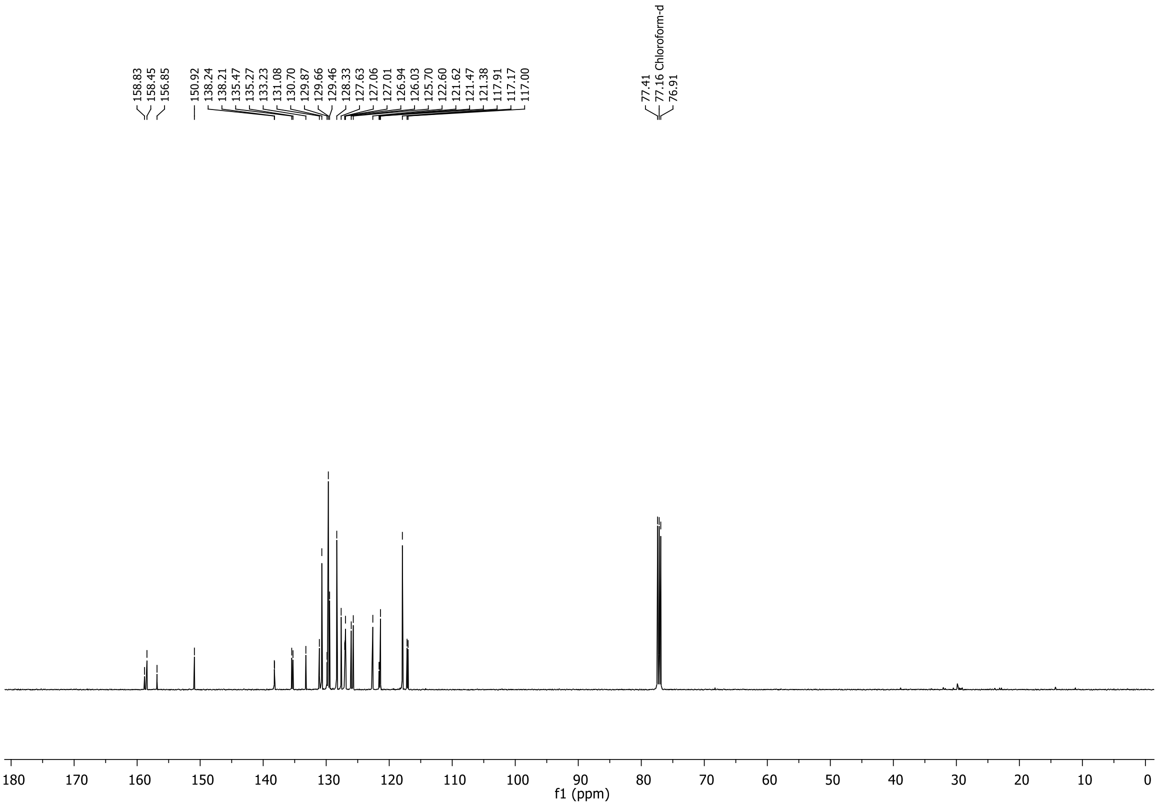

2-(4-chlorophenoxy)-1-(4-chlorophenyl)naphthalene (3a)

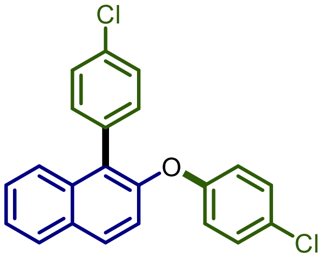

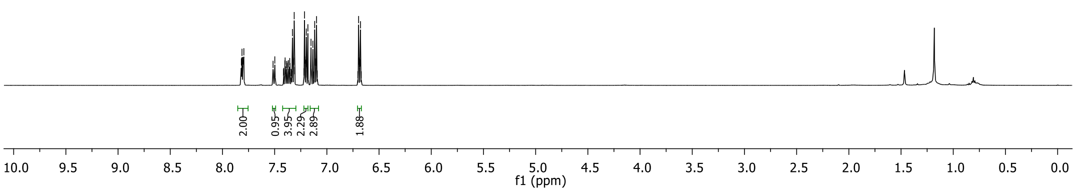

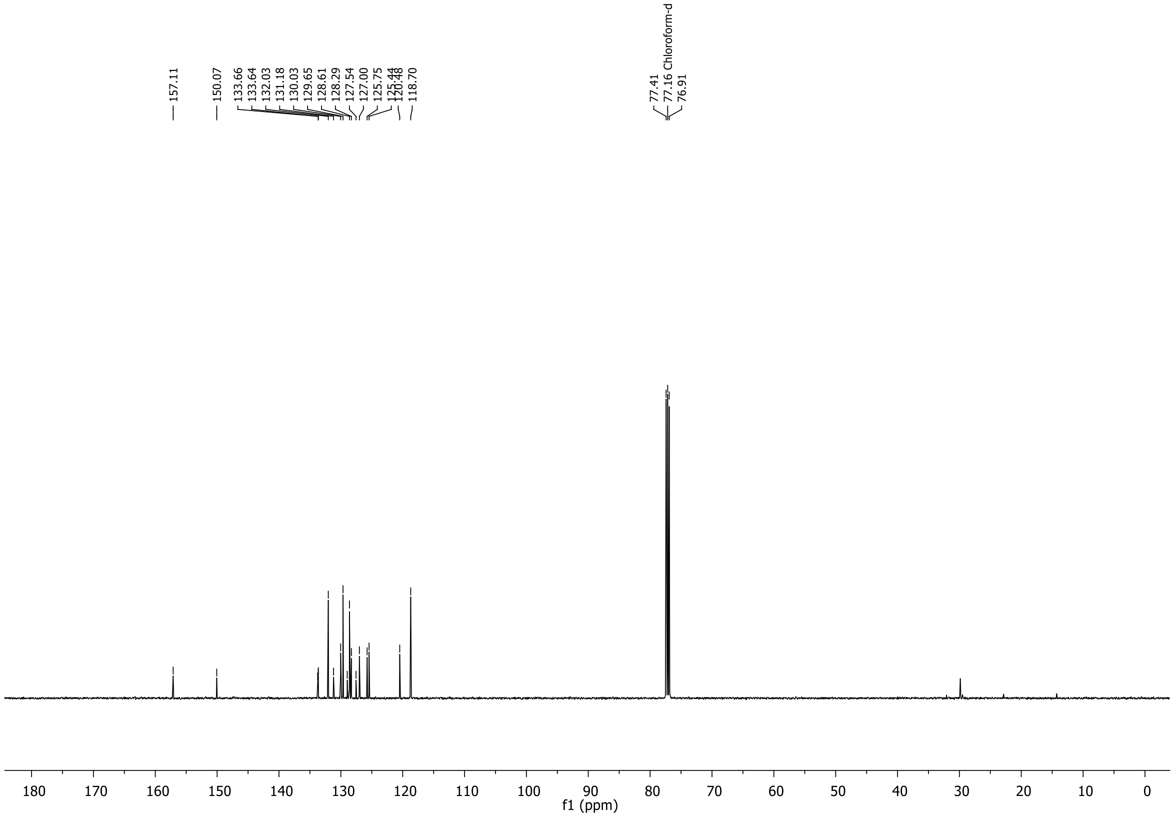

2-(4-nitrophenoxy)-1-(4-nitrophenyl)naphthalene (3b)

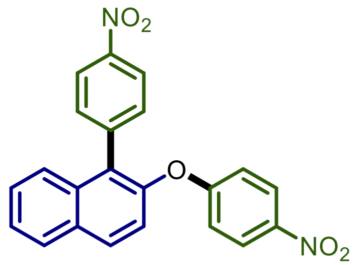

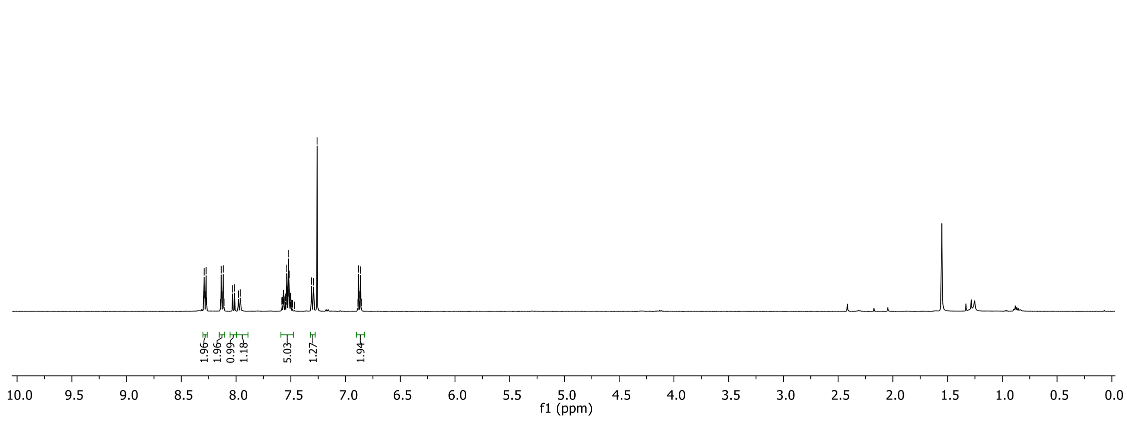

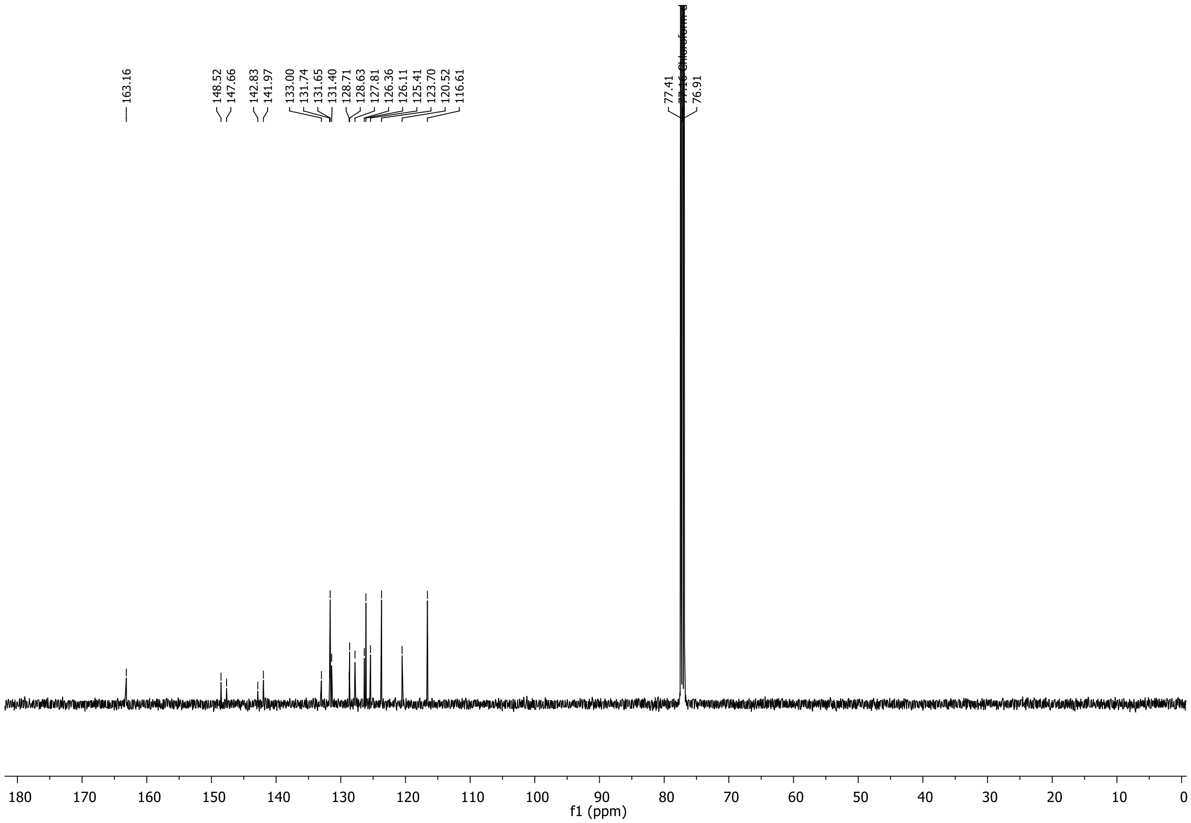

6-bromo-2-(4-chlorophenoxy)-1-(4-chlorophenyl)naphthalene (3c)

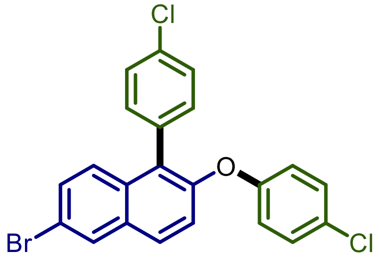

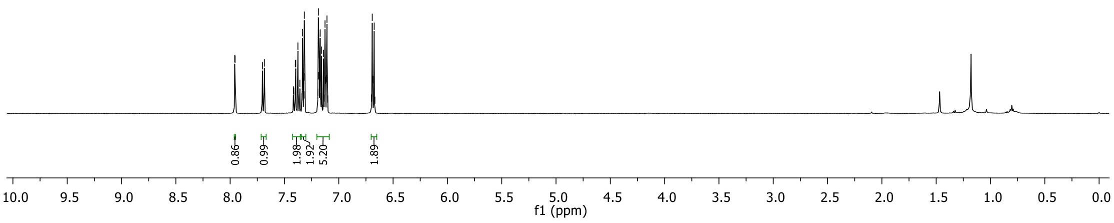

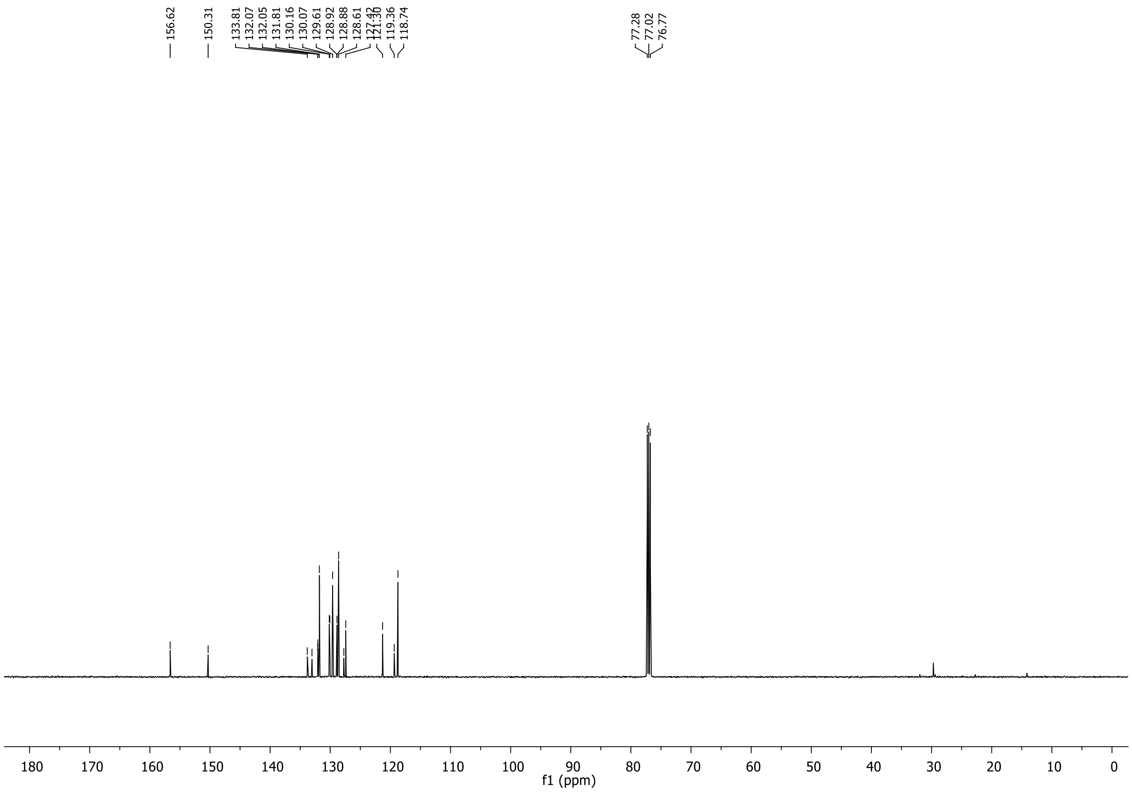

3-bromo-2-(4-chlorophenoxy)-1-(4-chlorophenyl)naphthalene (3d)

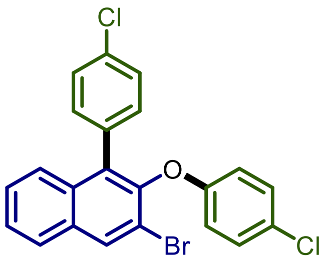

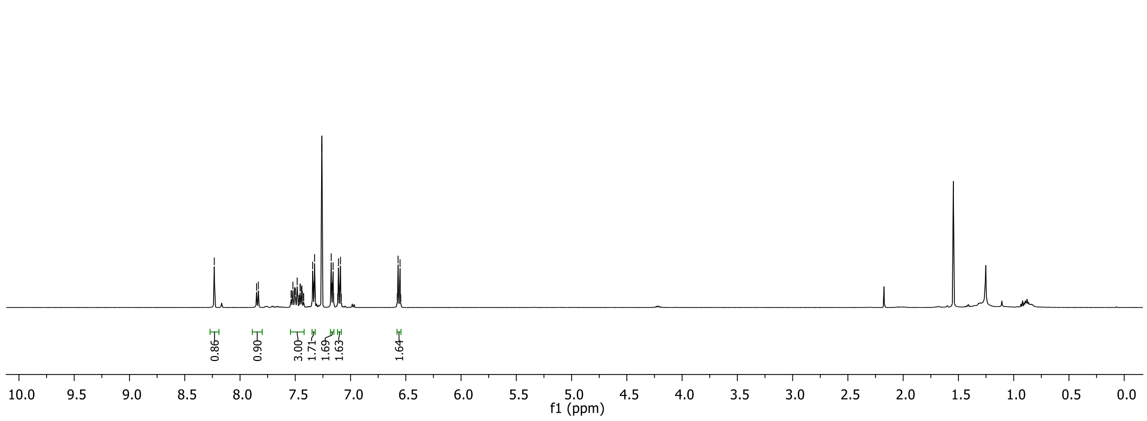

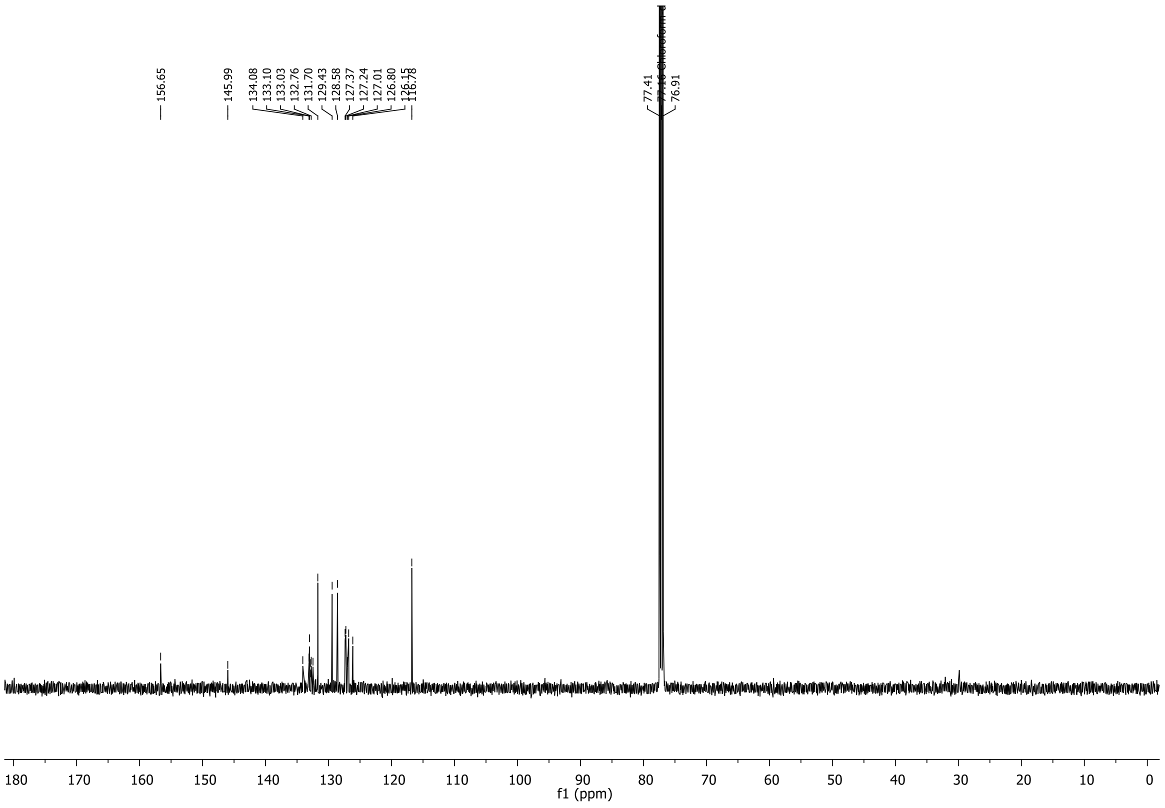

2-(4-chlorophenoxy)-1-(4-chlorophenyl)-6-phenylnaphthalene (3e)

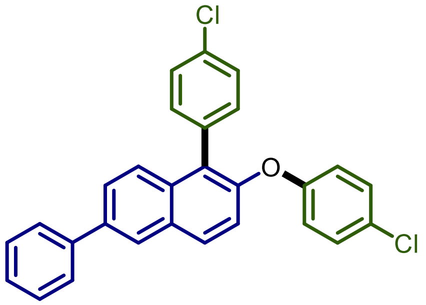

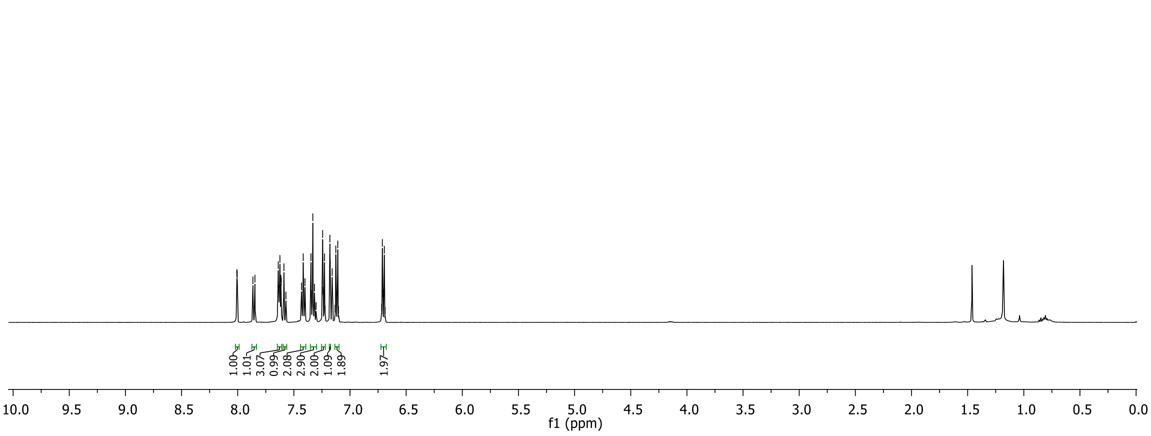

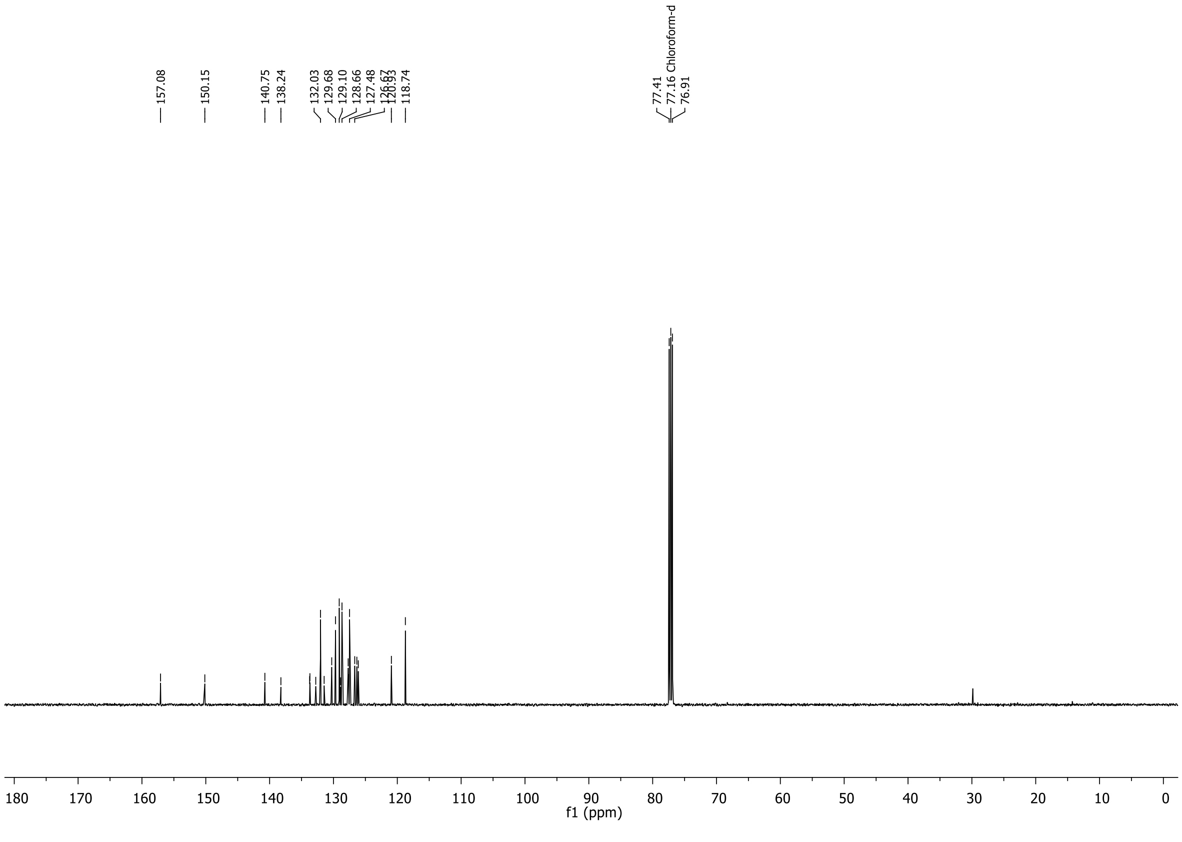

2-(4-chlorophenoxy)-1,6-bis(4-chlorophenyl)naphthalene (3f)

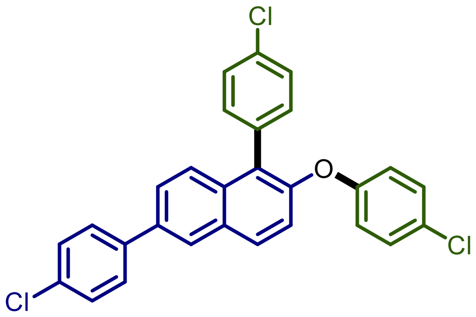

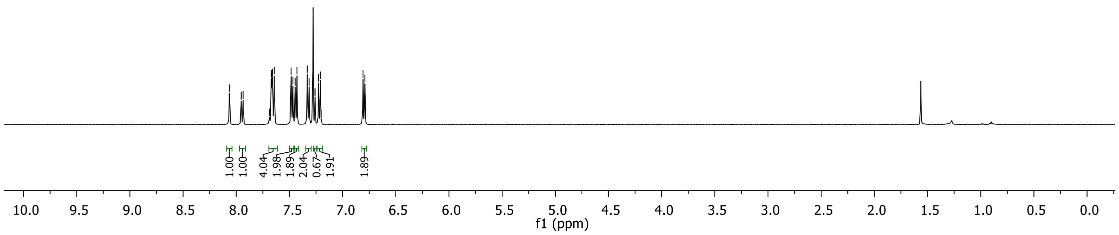

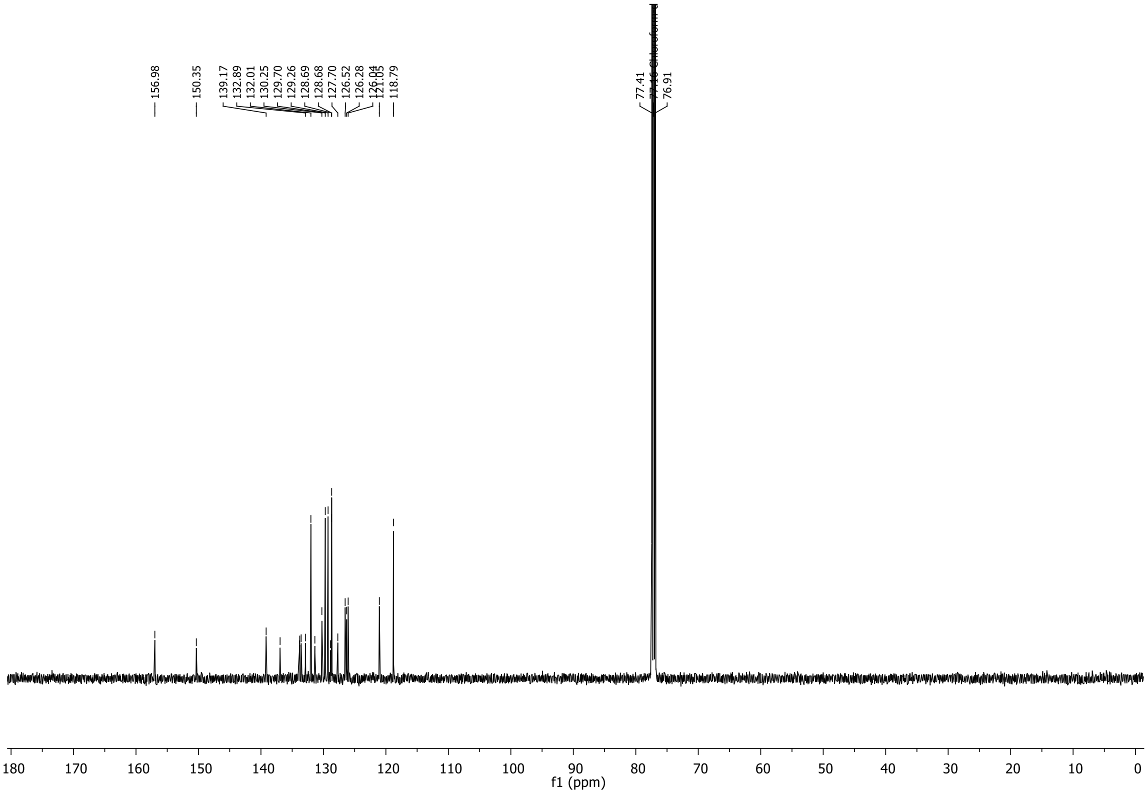

6-(3-chloro-4-fluorophenyl)-2-(4-chlorophenoxy)-1-(4-chlorophenyl)naphthalene (3g)

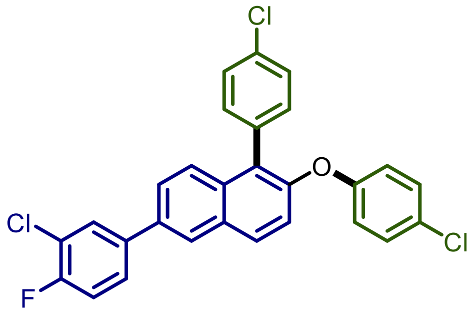

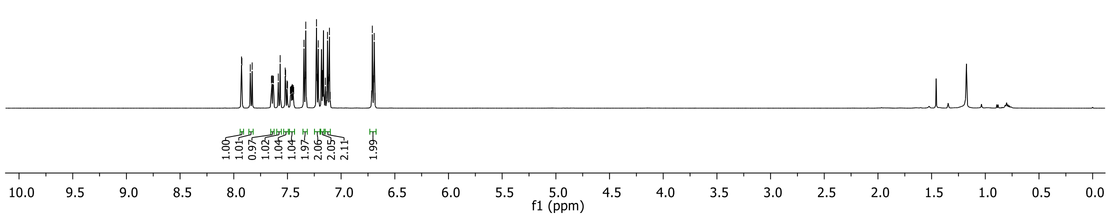

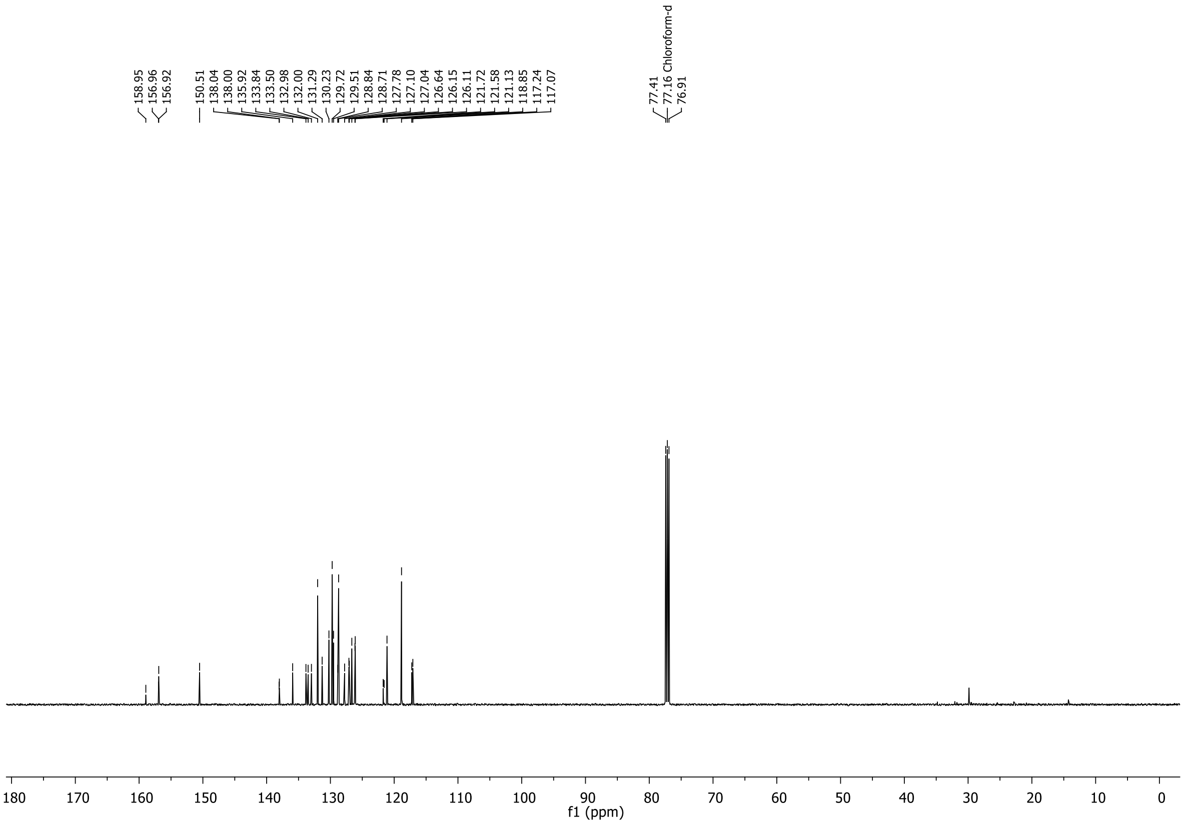

4-bromo-1-(4-chlorophenoxy)-2-(4-chlorophenyl)naphthalene (3h)

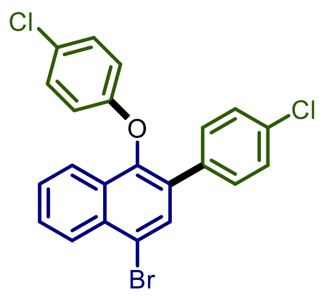

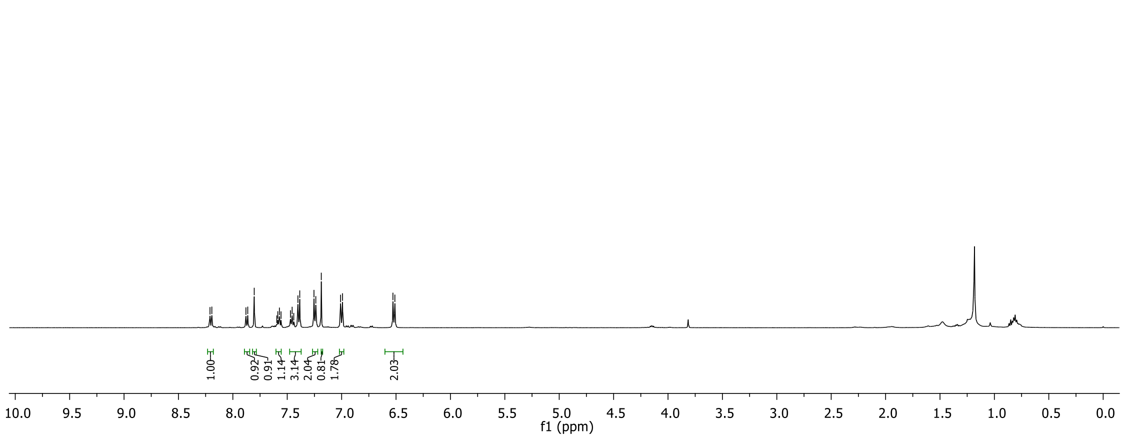

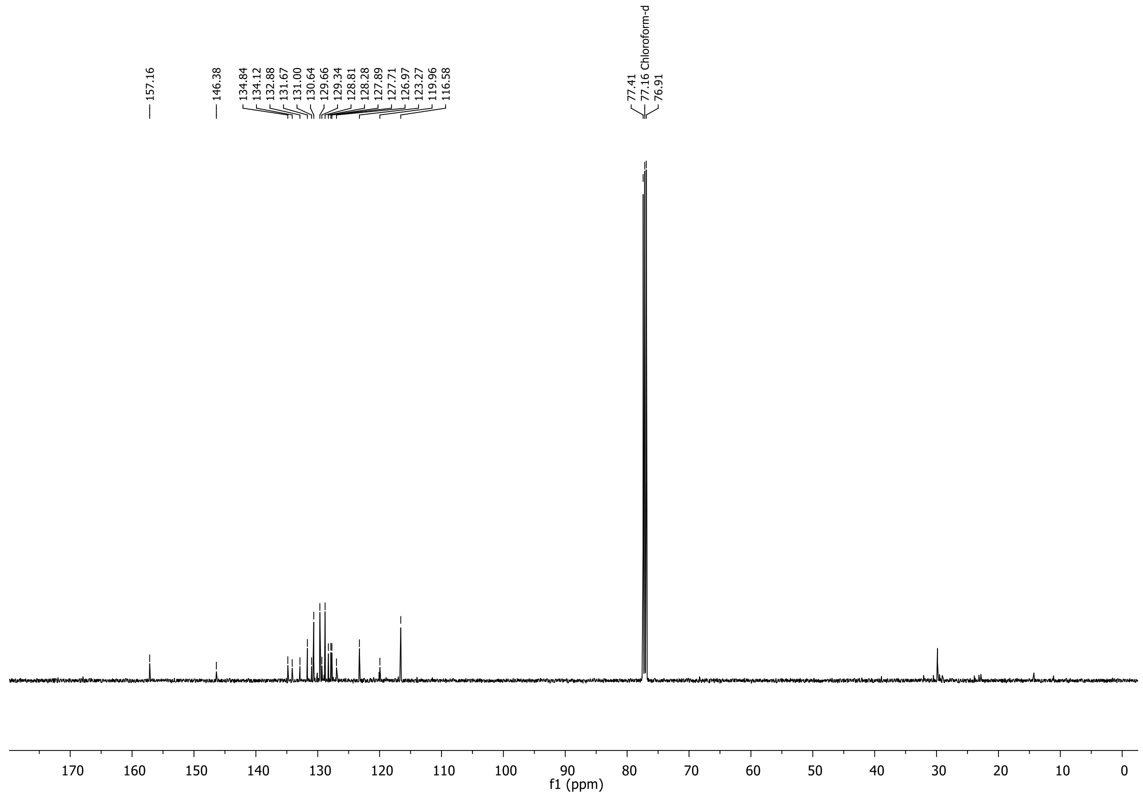

SPECTRA OF SYNTHETIC UTILITY

2-phenoxy-1,3-diphenylnaphthalene (4)

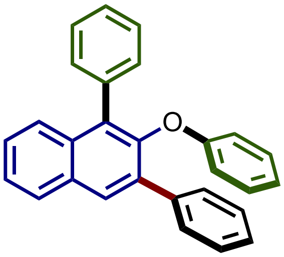

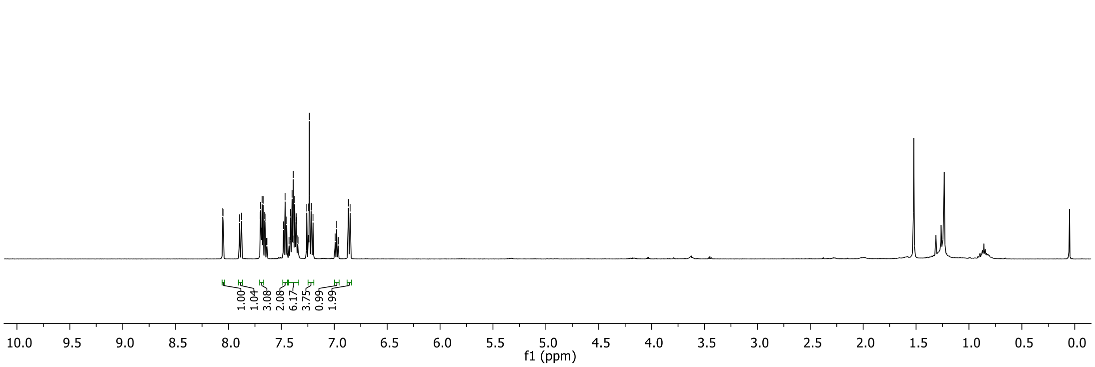

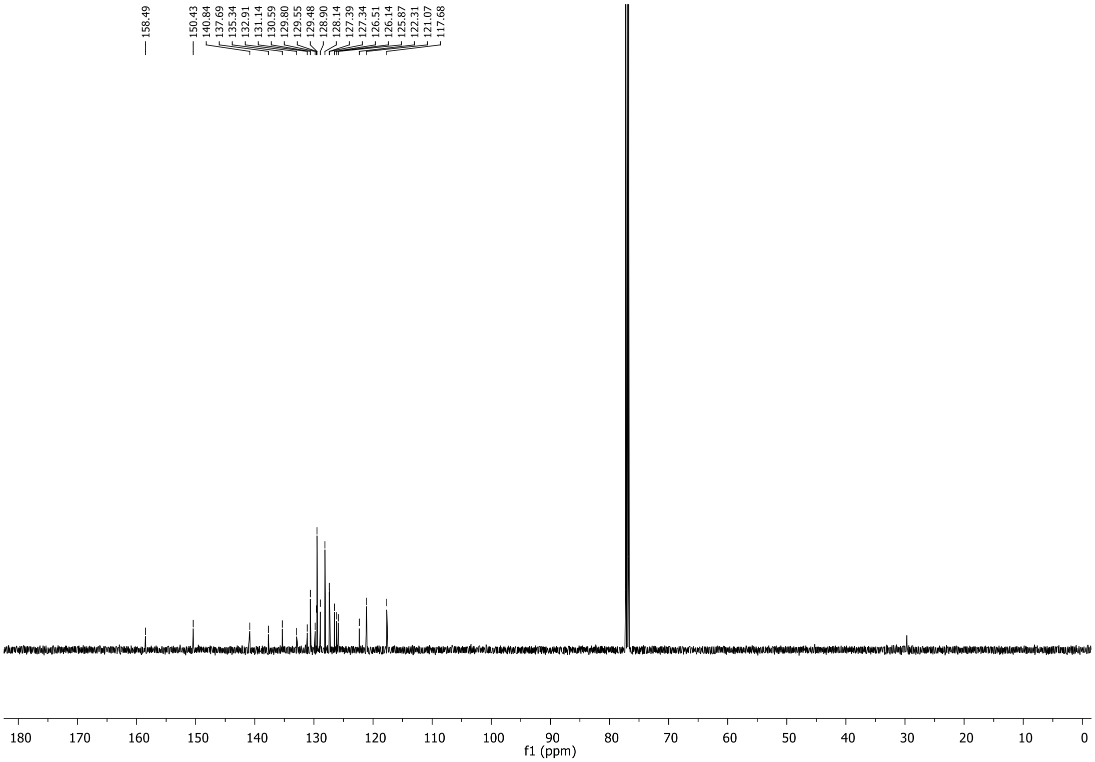

SPECTRA OF MECHANISTIC STUDIES

2-phenoxynaphthalene

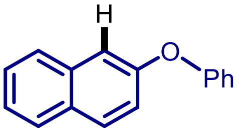

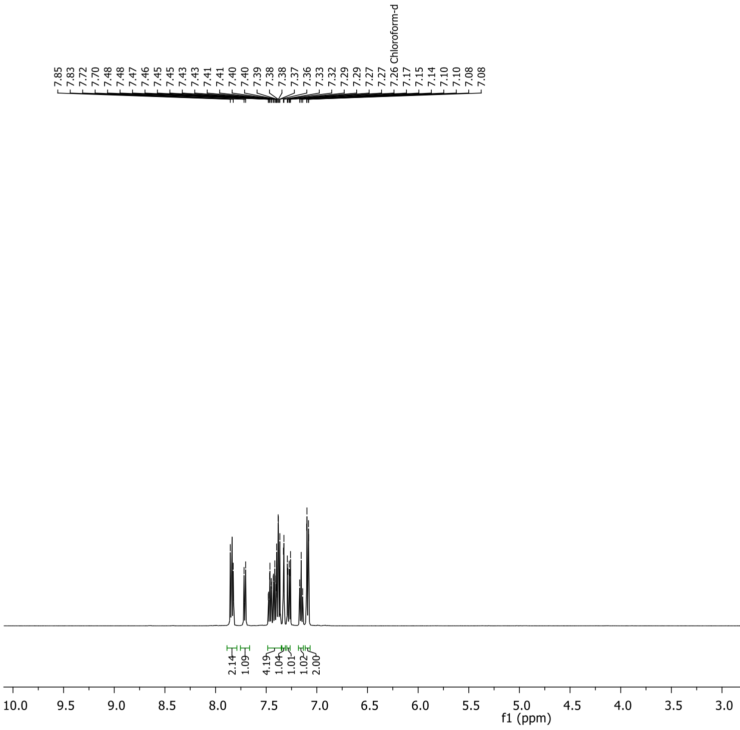

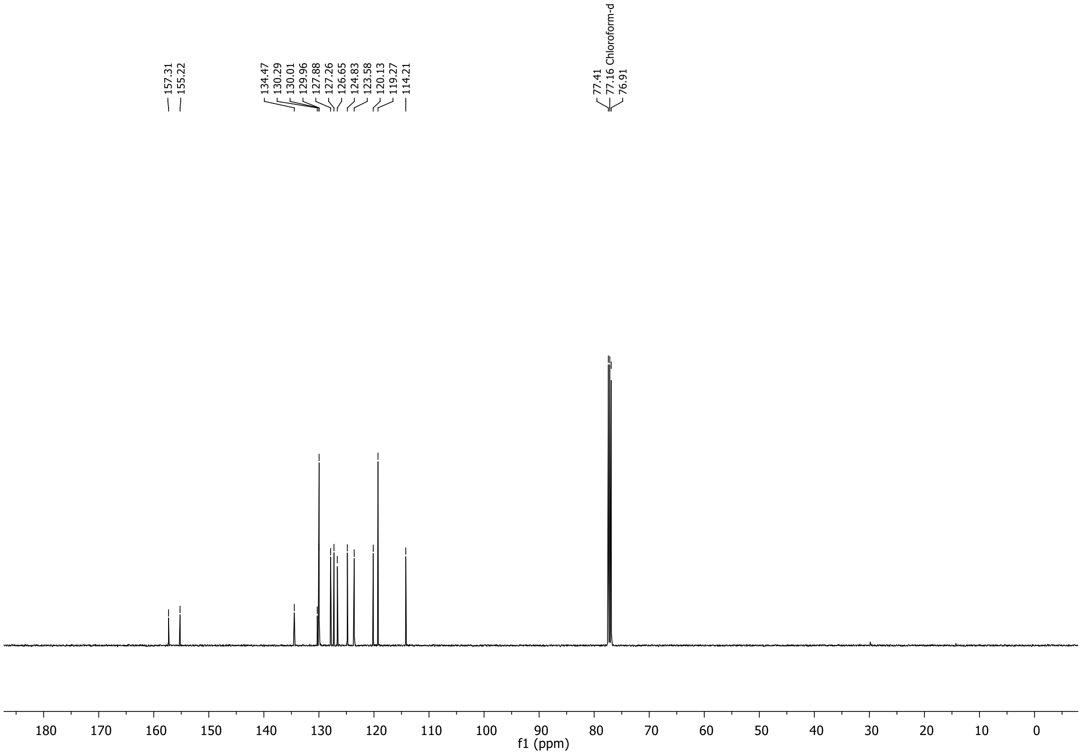

1-phenylnaphthalen-2-ol

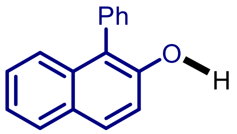

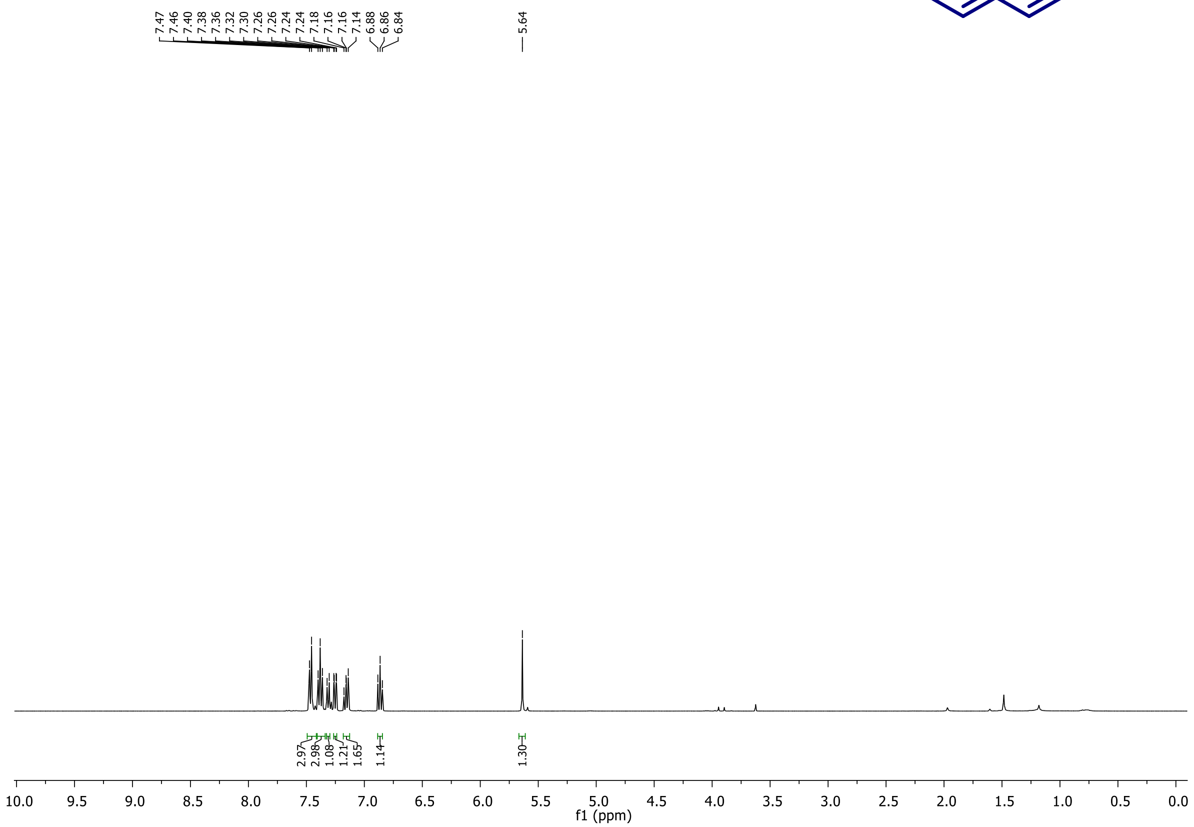

2-phenoxy-1-phenylnaphthalene

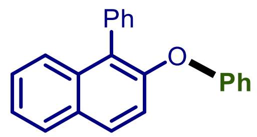

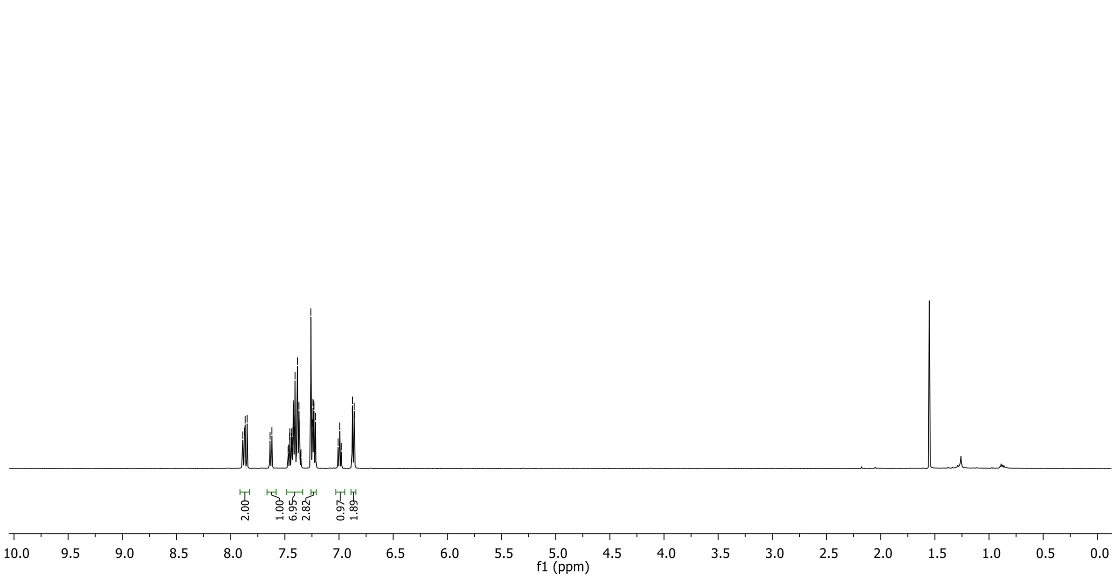

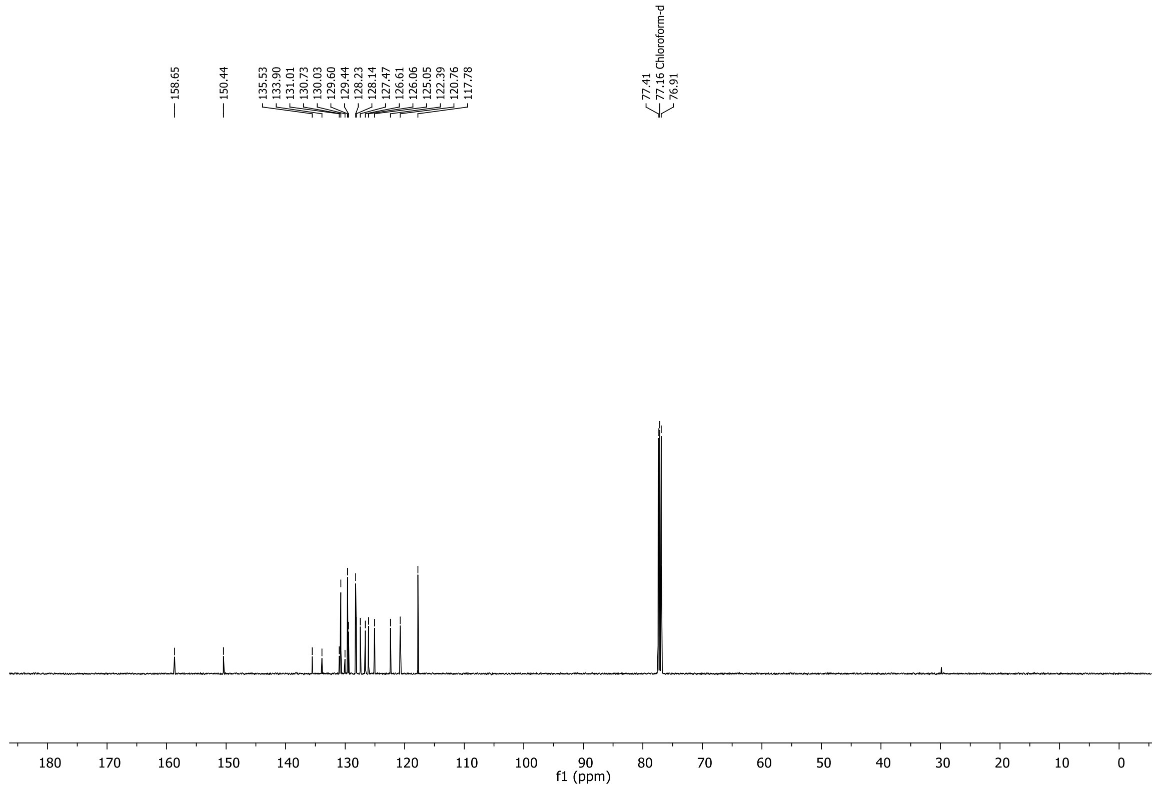

1-(2,2-diphenyl-1-(2,4,6-trinitrophenyl)hydrazinyl)-2,2,6,6-tetramethylpiperidine

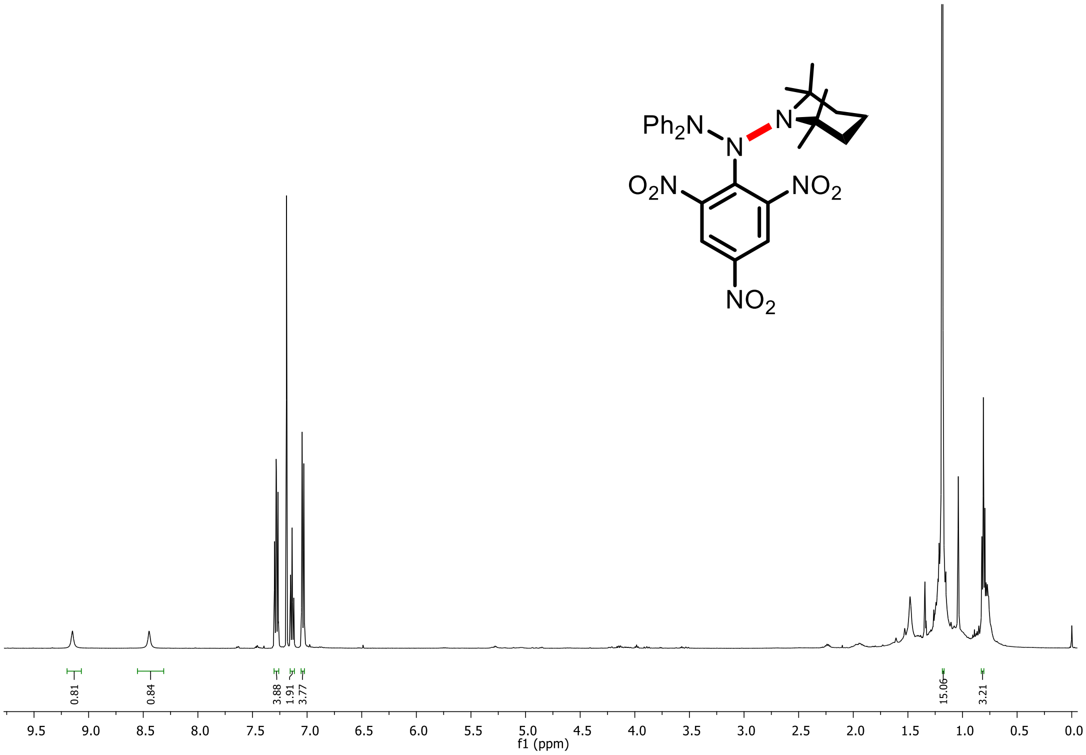

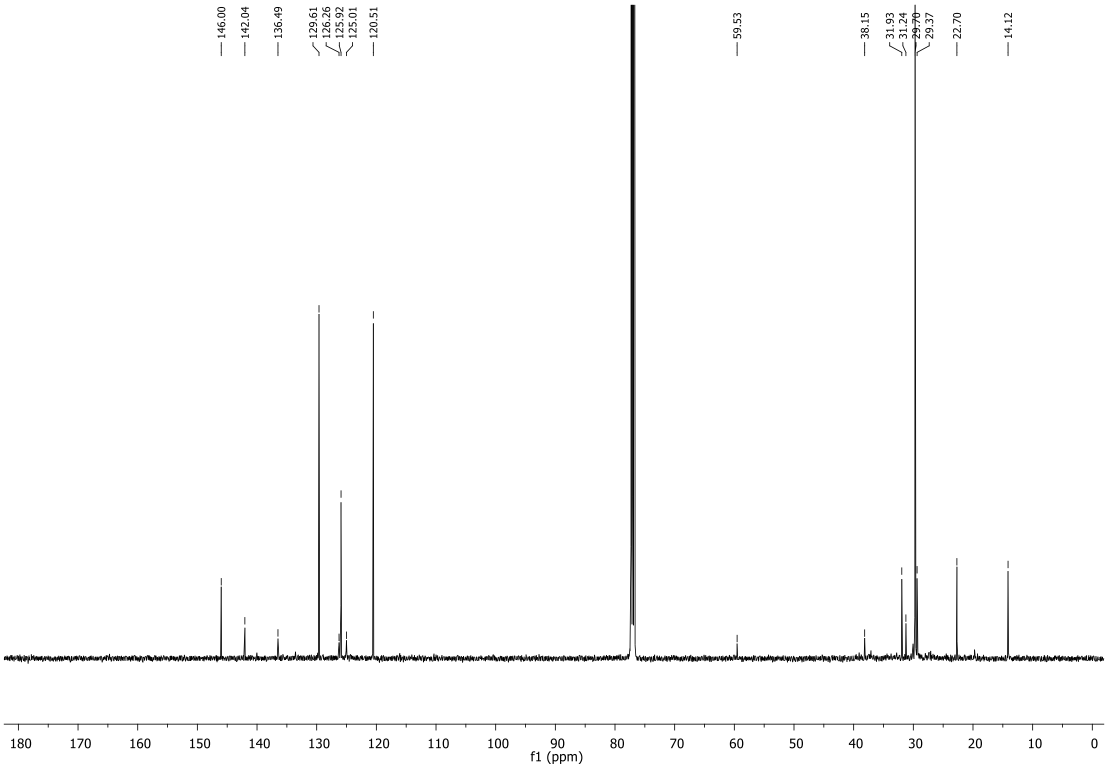

Triphenylamine

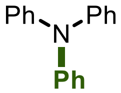

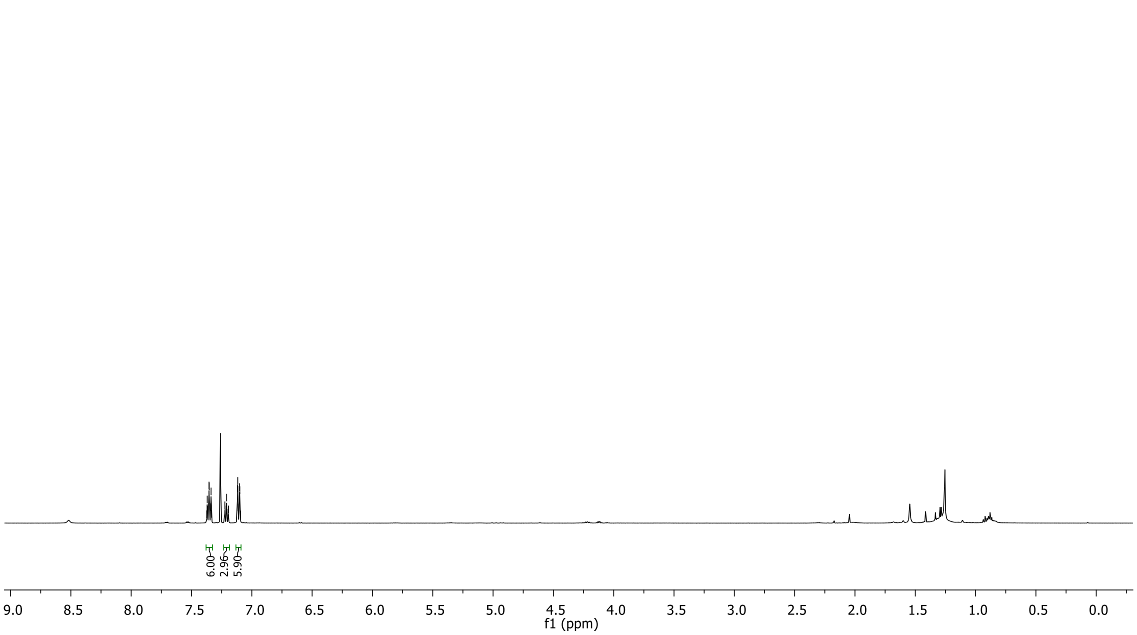

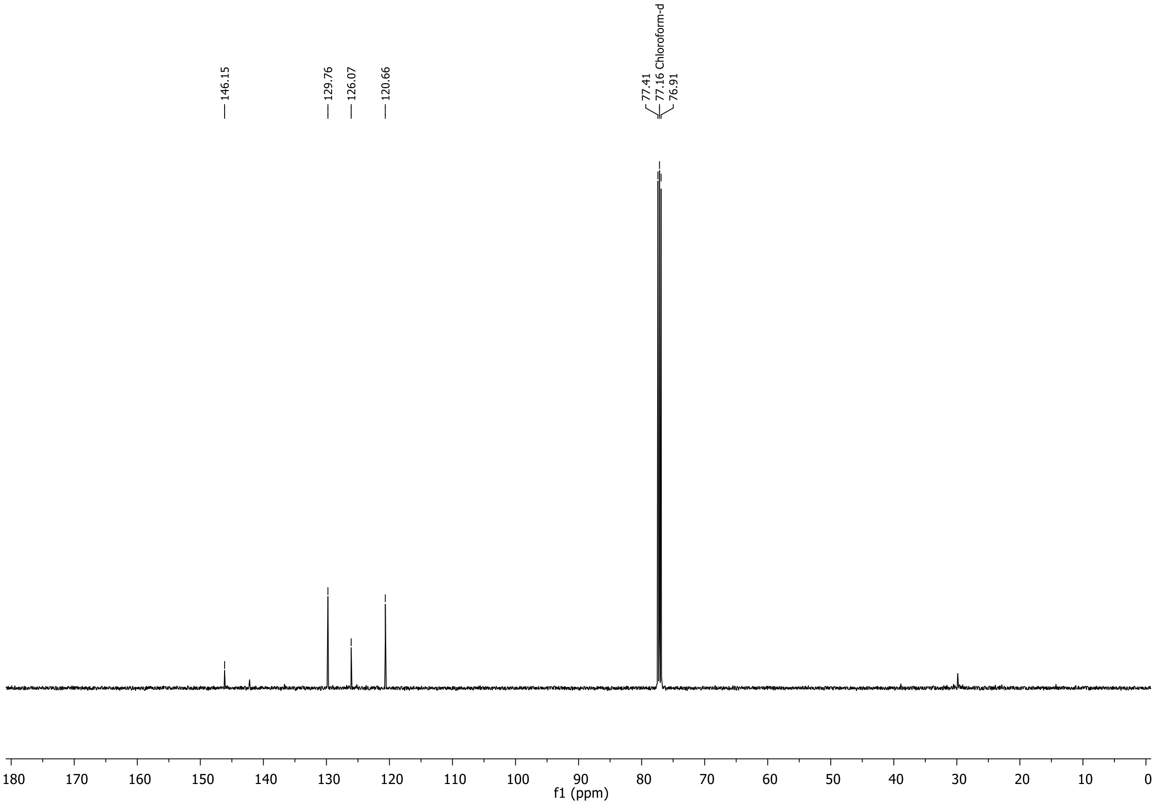

Triphenylamine

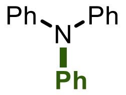

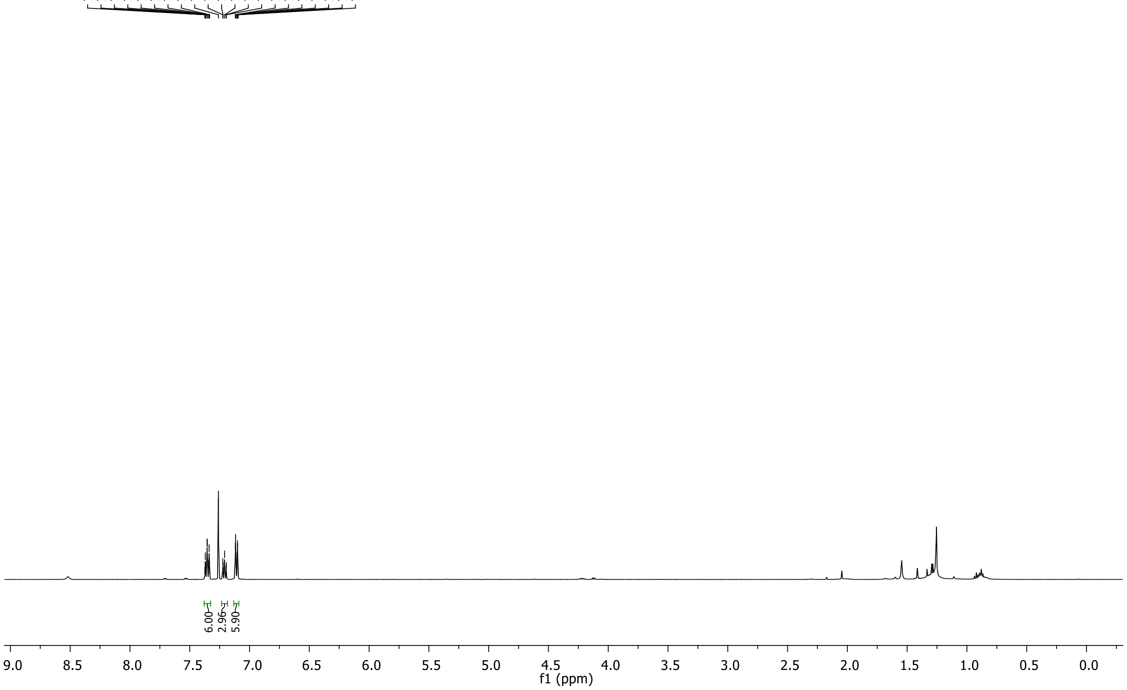

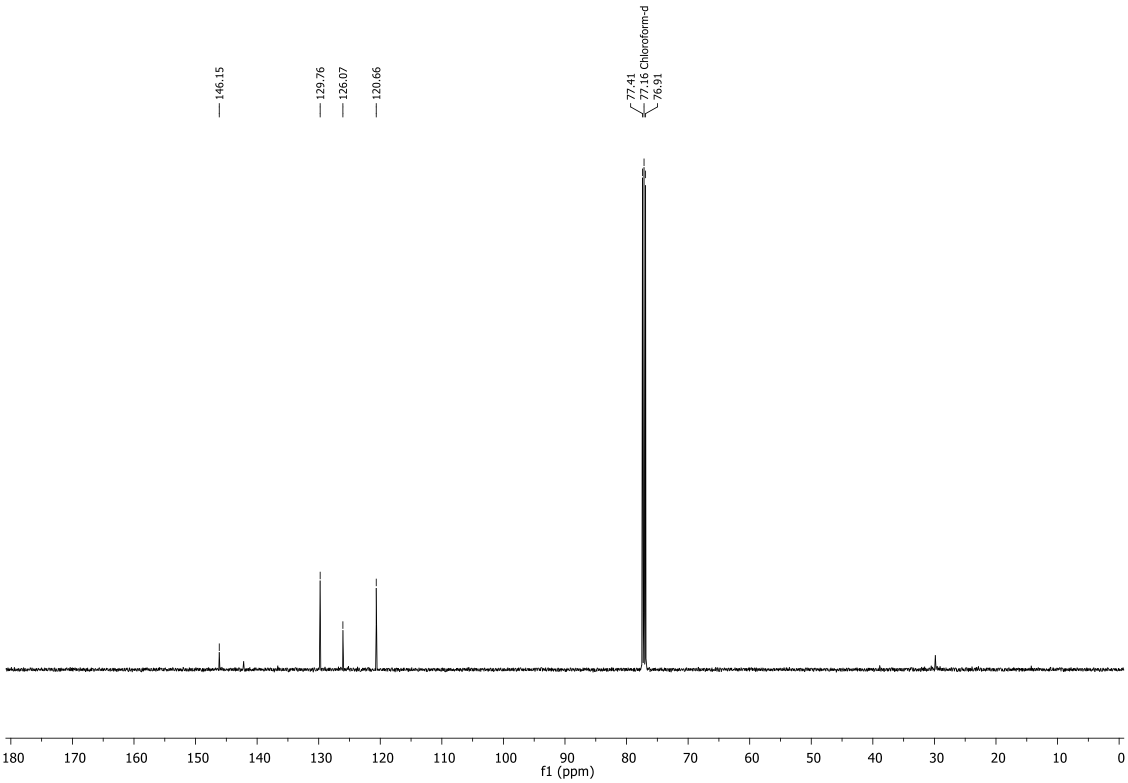

Dibenzo[*b,d*]furan (8)

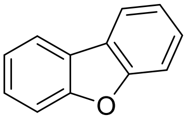

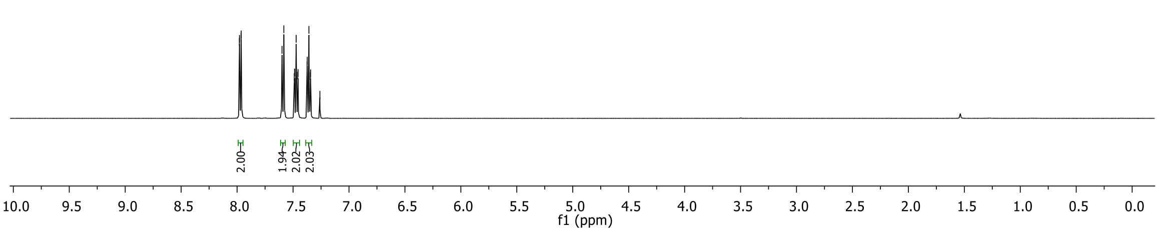

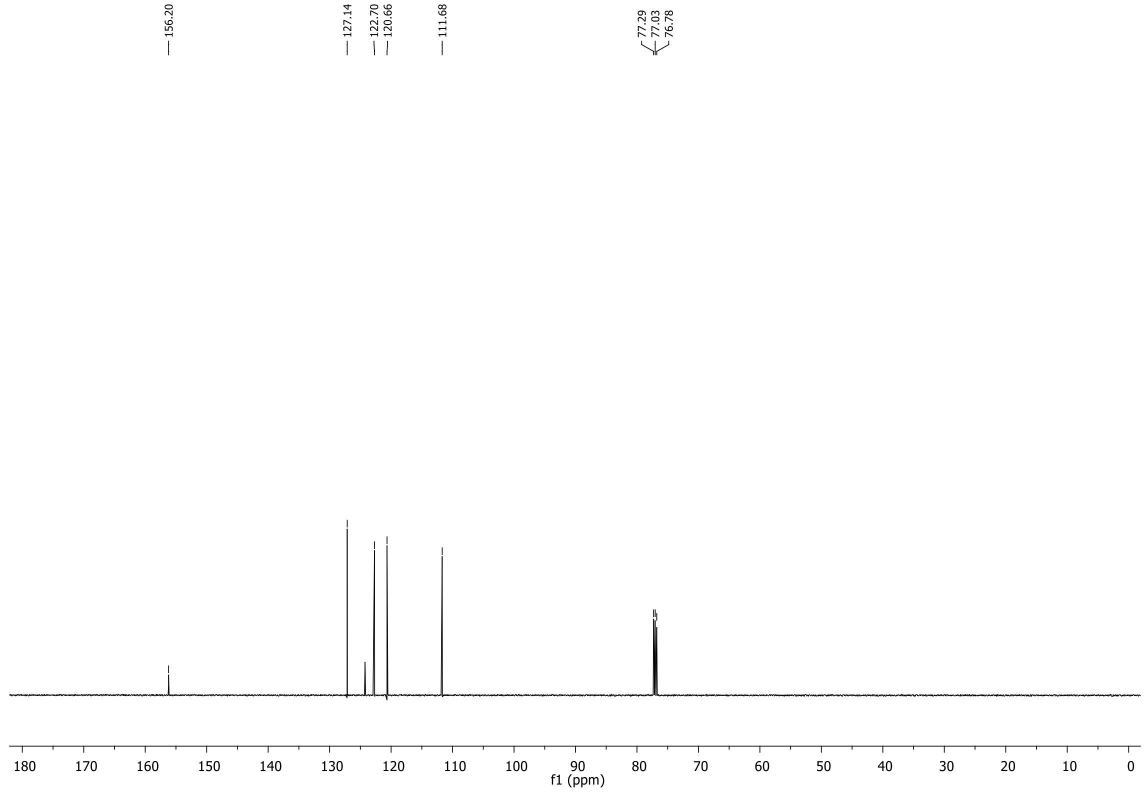

9-tosyl-*9H*-carbazole (9)

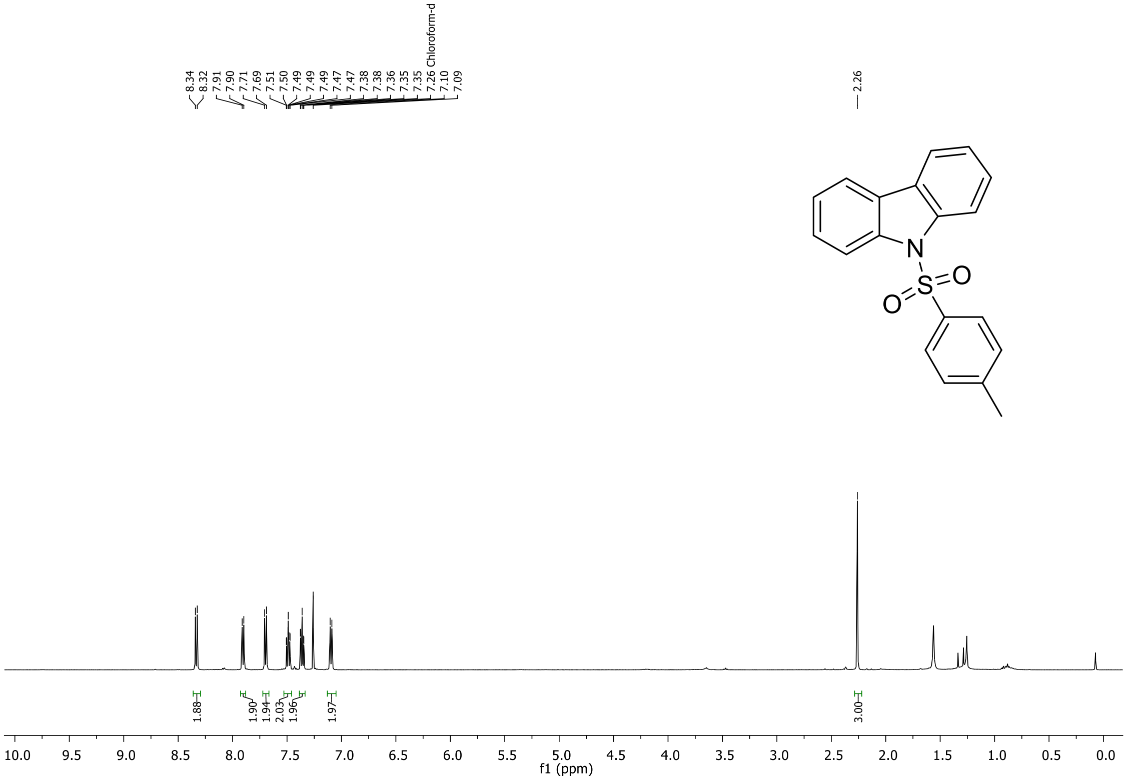

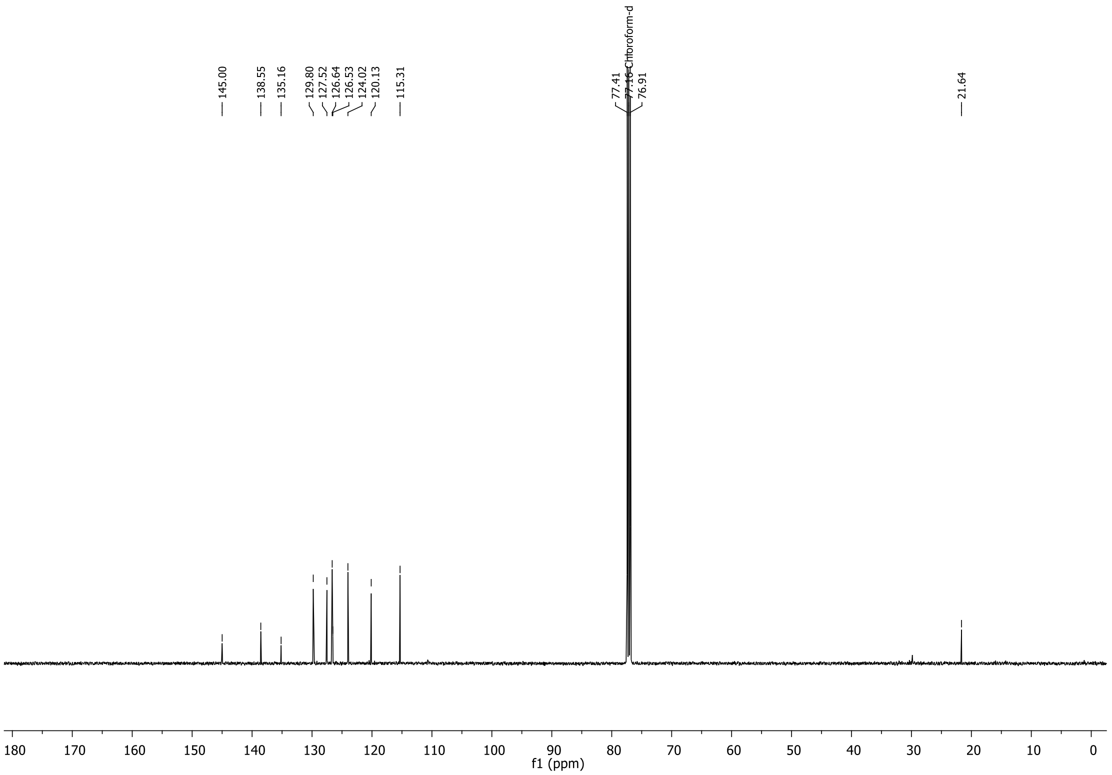

N-([1,1'-biphenyl]-2-yl)-4-methylbenzenesulfonamide (7)

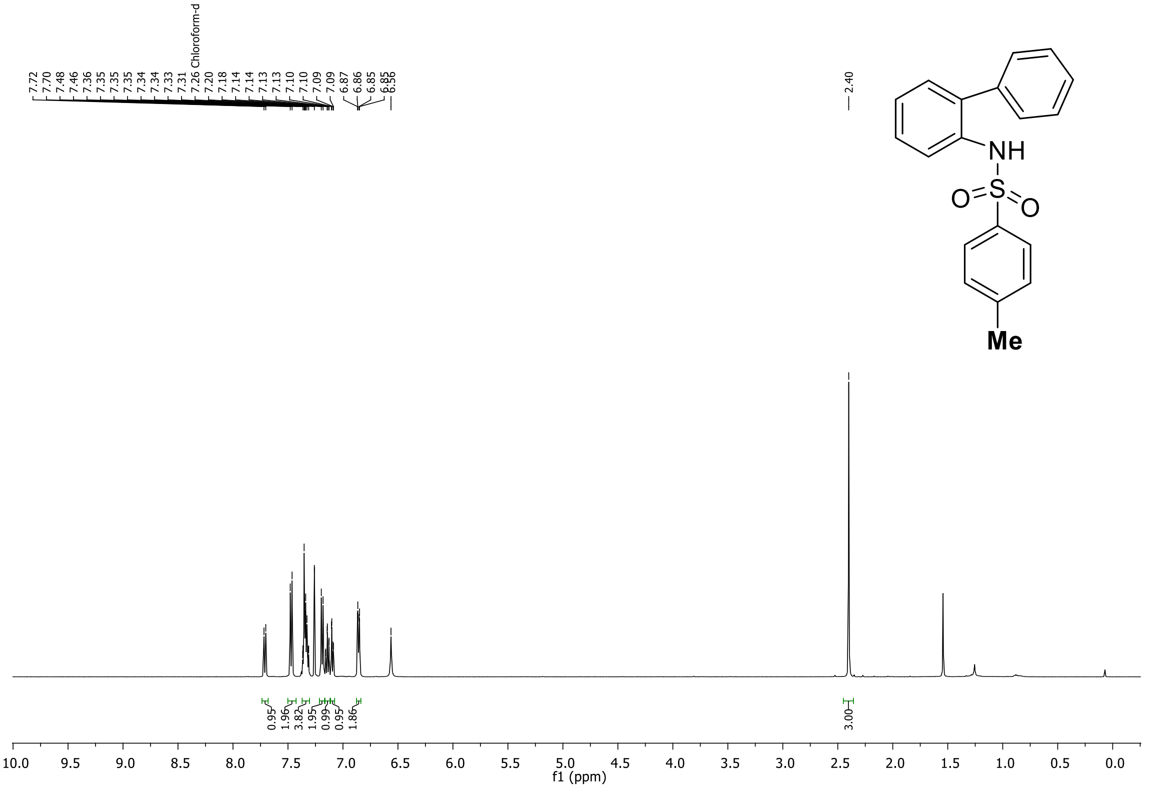

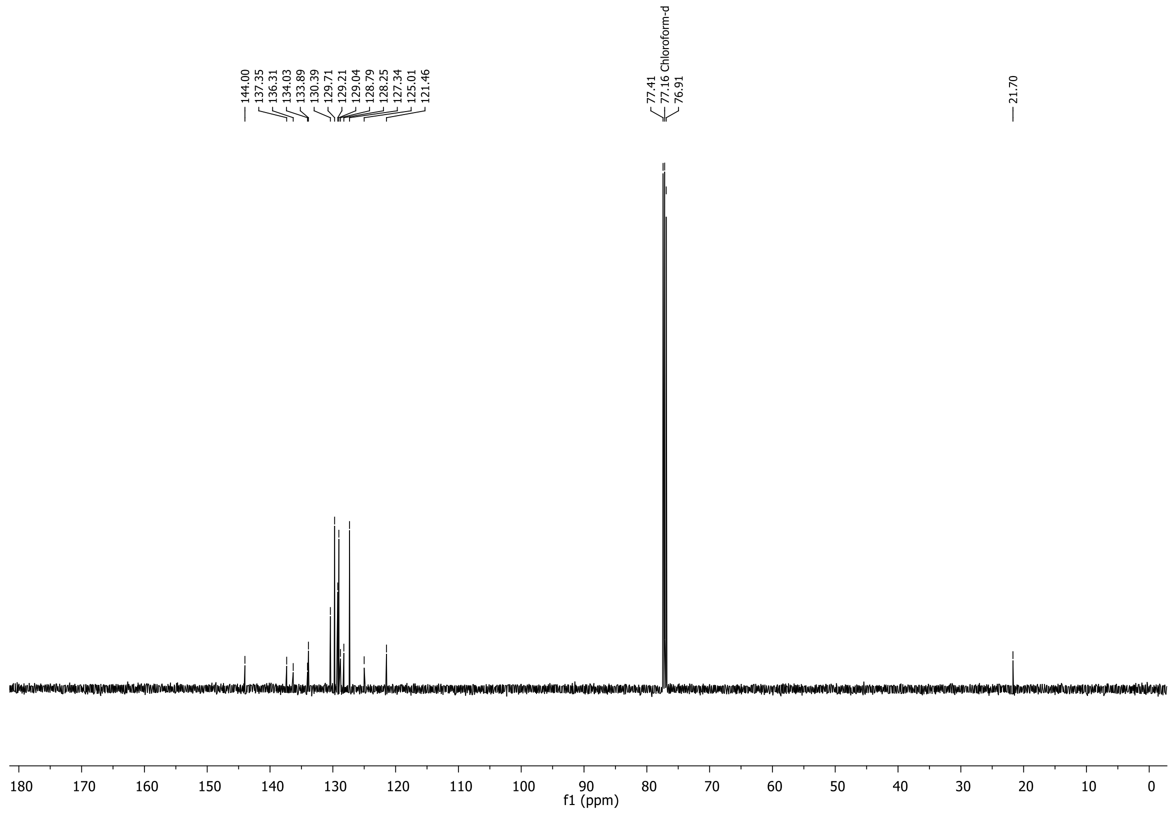

5. References

1. Ozanne-Beaudenon, A.; Quideau S. *Angew. Chem. Int. Ed.* **2005,** 44, 7065 –7069
2. Zhijian, L.; Larock, R. C. *Org. Lett.* **2004**, *6,* 3739-3741.
3. Zhijian, L.; Larock, R. C. *Tetrahedron.* **2007**, *63,* 347-355.
4. Mishra, K.; Biswas S. *J. Org. Chem.* **2016,** 81, 2355−2363.
5. Qian X.; Han J.; Wang, L. *Tetrahedron Letters*. **2016,** 57, 607–610.
6. Johnson, C.; Ansari, M.; Coop, A. *ACS Omega.* **2018,** 10886−10890.
7. M. J. Frisch, G. W. Trucks, H. B. Schlegel, G. E. Scuseria, M. A. Robb, J. R. Cheeseman, G. Scalmani, V. Barone, B. Mennucci, G. A. Petersson, H. Nakatsuji, M. Caricato, X. Li, H. P. Hratchian, A. F. Izmaylov, J. Bloino, G. Zheng, J. L. Sonnenberg, M. Hada, M. Ehara, K. Toyota, R. Fukuda, J. Hasegawa, M. Ishida, T. Nakajima, Y. Honda, O. Kitao, H. Nakai, T. Vreven, J. A. Montgomery, Jr., J. E. Peralta, F. Ogliaro, M. Bearpark, J. J. Heyd, E. Brothers, K. N. Kudin, V. N. Staroverov, R. Kobayashi, J. Normand, K. Raghavachari, A. Rendell, J. C. Burant, S. S. Iyengar, J. Tomasi, M. Cossi, N. Rega, J. M. Millam, M. Klene, J. E. Knox, J. B. Cross, V. Bakken, C. Adamo, J. Jaramillo, R. Gomperts, R. E. Stratmann, O. Yazyev, A. J. Austin, R. Cammi, C. Pomelli, J. W. Ochterski, R. L. Martin, K. Morokuma, V. G. Zakrzewski, G. A. Voth, P. Salvador, J. J. Dannenberg, S. Dapprich, A. D. Daniels, Ö. Farkas, J. B. Foresman, J. V. Ortiz, J. Cioslowski, and D. J. Fox, Gaussian, Inc., Wallingford CT, 2009. Gaussian 09, Revision C.01.
8. J.–D. Chai, M. Head-Gordon, Phys. Chem. Chem. Phys. 2008, 10, 6615.
9. L. E. Roy, P. J. Hay, R. L. Martin, J. Chem. Theory Comput. 2008, 4, 1029-1031.
10. (a) A. V. Marenich, C. J. Cramer, and D. G. Truhlar, J. Phys. Chem. B 2009, 113, 6378; (b) V. Barone and M. Cossi, J. Phys. Chem. A 1998, 102, 1995; (c) M. Cossi, V. Barone, B. Mennucci, and J. Tomasi, Chem. Phys. Lett. 1998, 286, 253; (d) V. Barone, M. Cossi, and J. Tomasi, J. Comp. Chem. 1998, 19, 404; (e) J. Tomasi, B. Mennucci, and R. Cammi, Chem. Rev. 2005, 105, 2999.
